# Horizontal gene transfer drives the evolution of Rh50 permeases in prokaryotes

**DOI:** 10.1186/s12862-016-0850-6

**Published:** 2017-01-03

**Authors:** Giorgio Matassi

**Affiliations:** Dipartimento di Scienze Agro-alimentari, Ambientali e Animali (DI4A), Università di Udine, Via delle Scienze, 206-33100 Udine, Italy

**Keywords:** HGT, RH50 ammonia permeases, Methanogens, Anammox, Ammonia-oxidizing bacteria, Oxygen minimum zones OMZ

## Abstract

**Background:**

Rh50 proteins belong to the family of ammonia permeases together with their Amt/MEP homologs. Ammonia permeases increase the permeability of NH_3_/NH_4_^+^ across cell membranes and are believed to be involved in excretion of toxic ammonia and in the maintenance of pH homeostasis. RH50 genes are widespread in eukaryotes but absent in land plants and fungi, and remarkably rare in prokaryotes. The evolutionary history of RH50 genes in prokaryotes is just beginning to be unveiled.

**Results:**

Here, a molecular phylogenetic approach suggests horizontal gene transfer (HGT) as a primary force driving the evolution and spread of RH50 among prokaryotes. In addition, the taxonomic distribution of the RH50 gene among prokaryotes turned out to be very narrow; a single-copy RH50 is present in the genome of only a small proportion of Bacteria, and, first evidence to date, in only three methanogens among Euryarchaea. The coexistence of RH50 and AMT in prokaryotes seems also a rare event. Finally, phylogenetic analyses were used to reconstruct the HGT network along which prokaryotic RH50 evolution has taken place.

**Conclusions:**

The eukaryotic or bacterial “origin” of the RH50 gene remains unsolved. The RH50 prokaryotic HGT network suggests a preferential directionality of transfer from aerobic to anaerobic organisms. The observed HGT events between archaeal methanogens, anaerobic and aerobic ammonia-oxidizing bacteria suggest that syntrophic relationships play a major role in the structuring of the network, and point to oxygen minimum zones as an ecological niche that might be of crucial importance for HGT-driven evolution.

**Electronic supplementary material:**

The online version of this article (doi:10.1186/s12862-016-0850-6) contains supplementary material, which is available to authorized users.

## Background

Horizontal gene transfer (HGT), the process whereby genetic material is exchanged between unrelated species, has challenged our perception of evolution and the metaphors used to describe it; indeed the interplay between tree-shaped and reticulate-shaped processes provides a more realistic account of evolution, especially in prokaryotes [1, 2].

HGT is acting pervasively at the molecular level to shape the evolution in prokaryotes ([3, 4], for reviews). To date, the vast majority of the reported cases of transfer of genetic material concern exchanges within the three domains of life, inter-domain phenomena mainly involving transfers from bacteria to archaea and from prokaryotes to eukaryotes [5–7]. In contrast, DNA transfer from eukaryotes to prokaryotes is a rare event and is mainly restricted to symbiotic or parasitic relationships [5, 7–9]. Moreover, once the foreign genetic material has entered the new host via different mechanisms (conjugation, transformation, phage transduction, nanotubes), intra-genomic elements come into play, notably integrons and transposases ([10, 11] for reviews). The first three are the main mechanisms of HGT in prokaryotes ([3, 5] for reviews).

The protein family denoted Amt/MEP/Rh comprises ammonium transporters, methylamine permeases, and Rh permeases. The biochemical function of Amt proteins as NH_3_/NH_4_^+^ permeases is fairly well established in bacteria, fungi and plants [12], yet the substrate specificity of Rh50 permeases, be it NH_3_/NH_4_^+^, CO_2_ or both, is still debated [13, 14]. An appealing proposal suggested that Rh proteins might act as non-specific gas channels for neutral small molecules, NH_3_, CO_2_, and O_2_ [15]. Along the same reasoning, depending on the cellular and/or external environmental pressures, or on the tissue/organ/species involved, the Rh50 permease would be recruited to facilitate the transport of different gaseous substrates.

Rh50 and Amt proteins are distant homologs and are also functionally related - the latter being far from a trivial statement. Indeed, the experimental evidence was obtained by demonstrating that human Rh50 proteins could act as an ammonia channel when expressed in yeast [16]. The Rh50 protein was thus identified as the long-sought ammonia channel in humans, thereby revealing that ammonia gas not only diffuses across biological membranes, as previously thought, but also needs a channel to facilitate its movement. Rh50 was also functionally characterized as an ammonia/um permease in the ammonia-oxidizing bacterium *Nitrosomonas europaea* [17], *Anopheles gambiae* [18], mice [19] and fish ([20] for a review).

The biological role of Rh50 and Amt channels (also misleadingly called “transporters”) is starting to emerge. Most of the experimental evidence indicates that they increase the permeability of NH_3_/NH_4_^+^ across cell membranes. This is crucial in organismal physiology, as it allows the maintenance of both pH and ammonium homeostasis, in the latter case avoiding the toxic effect of high ammonium concentrations. Moreover, their role in organismal development has also been reported, and knock-out/knock-down mutants were shown to affect embryonic development in the amoeba *Dictyostelium discoideum* [21] and the nematode *Caenorhabditis elegans* [22], and to be essential for larval brain development and function in the tunicate *Ciona intestinalis* [23].

Amt proteins are classified in two main families, Amt1 and Amt2 [24, 25]. Amt1-type proteins are specific to eukaryotes, whereas Amt2 are mainly found in prokaryotes. Yet the Amt2 family comprises also MEP proteins from fungi (absent from the Amt1 type), as well as from other eukaryotes, namely choanoflagellida, amoebozoa, euglenozoa, stramenopiles, land plants and green algae.

While AMT1 genes likely arose from an AMT2-like ancestor followed by vertical descent, phylogenetic analyses suggest that all the eukaryotic lineages in the AMT2 family originated from HGTs events [25–27].

The presence of AMT genes in prokaryotes is ubiquitous, yet most interestingly they are missing in vertebrates in which RH50 is present instead. RH50 genes (vertebrate paralogs being also named RHAG, RHBG, RHCG) code for 50 kDa proteins, hence their name; they are found, in single or multiple copies, in all eukaryotic genomes searched so far, with the notable exceptions of land plants and fungi. In the vertebrate lineage, a duplication event from an RH50-like ancestor gave origin to the fast-evolving RH30 genes (coding for 30 kDa proteins), whose human homologs carry the Rh blood-group antigens at the surface of red-cells [28]. The evolution of RH30 genes will not be dealt with here.

Most interestingly, AMT and RH50 genes coexist (single or multicopy) in a range of eukaryotes, namely green-algae, dictyostelids, choanoflagellates as well as in metazoans such as cnidarians, nematodes, insects, cephalochordates and tunicates. Yet, RH50 genes are extremely rare in prokaryotes; and in the only case of bacterial gene studied so far, it has been shown that the ammonia-oxidizing bacterium *N. europaea* acquired the RH50 gene via HGT [17].

While the impact of HGT on the evolution of the AMT families has been described in previous studies [25–27], the role of HGT in the evolution of RH50 in prokaryotes has not been investigated. Therefore, in the present work, I have reconstructed the potential trajectories along which RH50 has been evolving in prokaryotes, and correlated them with ecological and metabolic niches of the organisms coding for the permease. The whole of those trajectories are defined as the RH50 HGT network.

Here I present the analysis of four datasets supporting the role of HGT as the major driver in the evolution of RH50 genes in prokaryotes: (i) the analysis of phyletic patterns (i.e., taxonomic distribution), (ii) the molecular phylogeny of Rh50 proteins, (iii) the analysis of 121 chromosomal neighbouring genes of RH50 in 31 genomes, and the molecular phylogenies of 91 of them, (iv) the molecular phylogeny of the Amt homologs.

## Results

### The phyletic pattern of the RH50 genes in prokaryotes provides evidence of HGT

Out of 3,853 prokaryote genomes only 34 were found to code for a single copy of the RH50 gene, among which those of 3 euryarchaeal methanogens (Additional file 1: Table S1). In addition, three paralogs are present in the parabasalian eukaryote *Trichomonas vaginalis* (Table 1; 30 prokaryotes are shown; refer to Methods). The number of AMT genes coded in each genome ranged from none to seven (Table 1).

**Table 1:** Phyletic patterns of RH50 and AMT genes

Taxonomy^a^	Species	Status^b^	RH50	AMT
Euryarchaeota/Methanomicrobia/Methanomassiliicoccales	*Methanomassiliicoccus luminyensis B10*	S/C	1	1
	*Ca.* Methanomassiliicoccus intestinalis Issoire-Mx1	Chr	1	0
Euryarchaeota/Methanomicrobia/Methanosarcinales	*Methanosalsum zhilinae* WeN5 DSM 4017	Chr	1	1
Bacteria/Planctomycetes	*Ca.* Kuenenia stuttgartiensis	C	1	4
	*Planctomycetaceae bacterium* KSU-1	S/C	1	7
	*Ca.* Brocadia anammoxidans (WQC04)^c^	nd	1	5
Bacteria/Proteobacteria/Betaproteobacteria	*Nitrosomonas europaea* ATCC 19718	Chr	1	0
	*Nitrosomonas sp.* Is79A3	Chr	1	0
	*Nitrosomonas sp.* AL212	Chr	1	0
	*Nitrosospira multiformis* ATCC 25196	Chr	1	0
	*Nitrosospira briensis C-128*	PD	1	0
	*Nitrosospira sp.* APG3	S/C	1	0
Bacteria/Proteobacteria/Deltaproteobacteria	*Geobacter sp. M21*	Chr	1	2
Bacteria/Firmicutes/Clostridia/Clostridiales/Clostridiaceae	*Clostridium papyrosolvens* DSM 2782	S/C	1	3
	*Clostridium papyrosolvens C7*	S/C	1	2
	*Clostridium cellulovorans 743B*	Chr	1	2
	*Clostridium carboxidivorans P7*	S/C	1	1
	*Clostridium viride* DSM6836	PD	1	0
	*Clostridium sp.* BNL1100	Chr	1	2
	*Clostridium scatologenes* ATCC 25775	PD	1	3
Bacteria/Firmicutes/Clostridia/Clostridiales/Eubacteriaceae	*Eubacterium acidaminophilum* al-2, DSM 3953	C	1	0
Bacteria/Firmicutes/Clostridia/Clostridiales/Peptococcaceae	*Dehalobacter sp. 11DCA*	Chr	1	2
	*Desulfotomaculum acetoxidans* DSM 771	Chr	1	3
Bacteria/Firmicutes/Clostridia/Clostridiales/Peptostreptococcaceae	*Clostridium litorale W6*	PD	1	0
Bacteria/Firmicutes/Clostridia/Clostridiales/Ruminococcaceae	*Acetivibrio cellulolyticus CD2*	S/C	1	3
	*Bacteroides cellulosolvens* DSM 2933	PD	1	4
Bacteria/Firmicutes/Clostridia/Halanaerobiales	*Acetohalobium arabaticum* DSM 5501	Chr	1	1
Bacteria/Firmicutes/Clostridia/Clostridiales/Family XIII *incertae sedis*	*Anaerovorax odorimutans* DSM 5092	PD	1	0
Bacteria/Actinobacteria	*Citricoccus sp.* CH26A	S/C	1	1
Bacteria/Fibrobacteres-Acidobacteria group	*Candidatus* Koribacter versatilis Ellin345	Chr	1	2
Eukaryota/Parabasalia	*Trichomonas vaginalis G3*	S/C	3	0

^a^NCBI taxonomy, except for Methanomassiliicoccales [34]

^b^Genome status. NCBI (Chr = Chromosome; S/C = Scaffolds/Contigs). IMG (C = Complete; PD = Permanent Draft)

^c^The Brocadia genome (NCBI taxid:174632) was removed from the JGI-IMG database on March 2014

Next, the proportion of genomes coding for RH50 with respect to the number of currently sequenced genomes in the corresponding Phylum was assessed. The disproportion of RH50-coding genomes is flagrant. This was found among Bacteria (Additional file 1: Table S1) and among Archaea (Additional file 1: Table S2). Moreover, out of 28 Planctomycetes genomes, RH50 genes were present only in five anaerobic-ammonia oxidizing bacteria (anammox) and out of 820 Actinobacterial genomes, only in *Citricoccus sp.* CH26A (Additional file 1: Tables S1 and S3).

### The Rh50 phylogeny discloses several HGT trajectories in prokaryotes

Provided enough taxon-sampling is available, phylogenetic analysis remains the most powerful method to detect the likely occurrence of a HGT event [5, 29].

The number of sequenced RH50 genes in eukaryotes is currently of several hundred (see Background). The Rh50 dataset analysed here consisted of 90 taxa. Because of the unexpected position of *T. vaginalis* (see below), the taxon sampling of early diverging microbial eukaryotes was expanded by blast-searching the NCGR’s Marine Microbial Eukaryote Transcriptome Sequencing Project dataset (http://marinemicroeukaryotes.org).

ProtTest identified LG + Γ4 + F as the best evolutionary model fitting the data, according to all the statistics implemented. However, a cross-validation procedure favoured CATGTR over LG (likelihood mean score difference = 76.16 ± 26.72). The Rh50 phylogeny under the LG + Γ4 model was almost identical to the one inferred with CATGTR + Γ4, therefore an artefact due to long-branch attraction cannot account for the observed topology. For clarity of presentation and discussion, three clades in the tree are defined: the eukaryotic clade (henceforth Rh50_euk), the prokaryotic clades (Rh50_prok) and its sub-clade Rh50_prok_meth (named after methanogens).

**Fig. 1:**
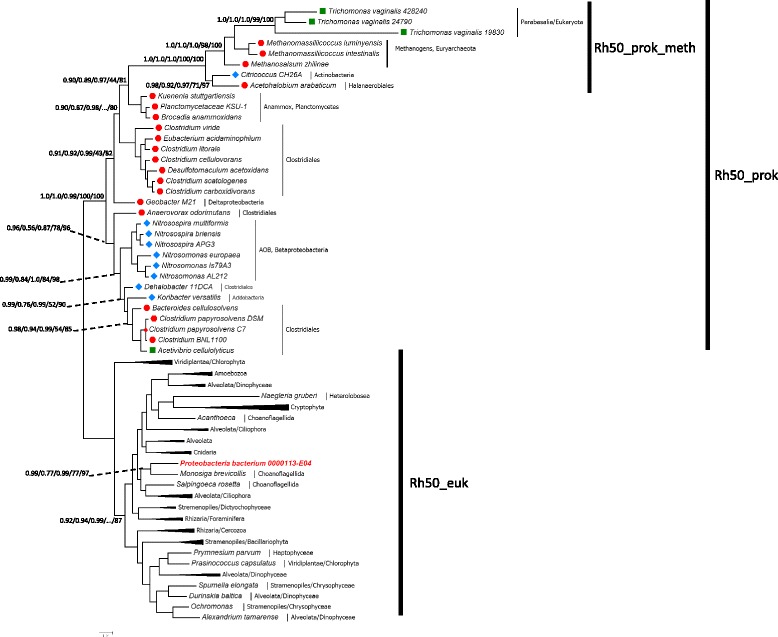
Unrooted phylogeny of Rh50 proteins (90 taxa, 252 sites). Bayesian majority-rule consensus of 1,076 trees obtained under the LG + Γ4 mixture model in PhyloBayes. Branch-support values given at nodes are PP1/PP2/aBayes/RBS/UFBoot. PP1 = Bayesian Posterior Probabilities (LG + Γ4; PhyloBayes), PP2 = Bayesian Posterior Probabilities (CATGTR + Γ4; PhyloBayes); aBayes = Maximum likelihood Bayesian-like aLTR transformation (LG + Γ4 + F; PhyML); RBS = Maximum likelihood rapid-bootstrap support (LG + Γ4 + F; RAxML); UFBoot = Maximum likelihood ultrafast-bootstrap approximation (LG + Γ4 + F; IQ-TREE). Some clades are condensed for clarity of presentation (for full tree see Additional file 2: Figure S1). Species oxygen requirements are denoted as follows: anaerobic (red dot), facultative (green square), aerobic (blue diamond). *Proteobacteria bacterium* JGI 0000113-E04 is highlighted in red. The genome of *Proteobacteria bacterium* JGI 0000113-E04 is deposited at the IMG-JGI (GOLD project ID = Gp0024568). Several lines of evidence suggest that part of the scaffolds making up this genome belong to contaminants. The RH50 homolog (Locus tag = D341DRAFT_01802) is present at one end of scaffold00071.71. Its nearest neighbour on the scaffold (D341DRAFT_01804), which codes for a beta-galactosidase/beta-glucuronidase, is again sister to its *Monosiga* homolog in a Bayesian phylogeny (Additional file 2: Figure S2). The *P. bacterium* genome is issued from uncultured single-cell sequencing, and no genomic DNA is available, thus experimental tests cannot be performed. The scale-bar indicates the estimated number of substitutions per site

As for the Rh50_euk clade (full tree in Additional file 2: Figure S1), two results are noteworthy. First, the parabasalian *T. vaginalis* (Excavata) does not cluster with other microbial eukaryotes and in particular with the heterolobosean *Neagleria gruberi* (Excavata). Second, the Rh50_euk clade includes a sequence denoted *Proteobacteria bacterium*. Several lines of evidence indicate that the *P. bacterium* RH50 is a “contaminant” belonging a sister species to the choanoflagellate *Monosiga brevicollis* (Fig. 1 legend).

The Rh50_prok clade comprises 30 prokaryotes and the eukaryote *T. vaginalis*. The aerobic ammonia-oxidizing bacteria (AOB) *Nitrosomonas* and *Nitrosospira* (Betaproteobacteria) form a monophyletic group, while the Clostridiales (Firmicutes) do not (Fig. 1). The Acidobacteria *Ca.* K. versatilis is nested within the Clostridiales with significant branch support. *Geobacter M21* (Deltaproteobacteria) is consistently more closely related to the clade comprising Clostridiales, anammox and Rh50_prok_meth, while *Anaerovorax odorimutans* (Clostridiales) is sister to the clade including AOB and other Clostridiales.

Compositional bias, found in a number of sequences, did not seem to generate any artefact (see Methods). Incidentally, the compositional deviation in the aerobe *Citricoccus* is intriguing; whether this has functional implications would need to be tested experimentally.

The Rh50_prok_meth clade comprises 8 sequences in 6 species, namely three euryarchaeal methanogens, *T. vaginalis* (three paralogs), the Halanaerobiales clostridium *Acetohalobium arabaticum* and the Actinobacteria *Citricoccus*. The parabasalian *T. vaginalis* clustered within the archaeal methanogens in both BI and ML phylogenies (under all evolutionary models); the long branch of *T. vaginalis*_19830 was always placed at the same position in the tree irrespective of the phylogenetic method used (not shown).

The Rh50_prok_meth clade is also characterized by a long-branch. The site heterogeneous CATGTR model is less sensitive to long-branch attraction [30]. PhyloBayes BI under the CATGTR+G4 model recovered the sister relationship between Rh50_prok_meth and anammox, whereas the LG+G4 model did not (not shown). Two competing topologies for this clade were found: either as basal to the Rh50_prok clade or as sister to the anammox. Tree topology tests supported the sister relationship between Rh50_prok_meth and anammox (Additional file 2: Figure S4).

In summary, the phylogeny of the Rh50 proteins has established that the Rh50_prok_meth clade is sister to the anammox Planctomycetes and disclosed potential scenarios for the HGT of RH50 amongst prokaryotes.

### The phylogenies of RH50 chromosomal neighbours support multiple HGT scenarios in prokaryotes

Several sister-taxa relationships in the Rh50 phylogeny suggest the existence of potential HGT trajectories in the Rh50_prok clade (Fig. 1): anammox are sister to the RH_prok_meth clade, *A. arabaticum* to methanogens, *A. arabaticum* to *Citricoccus*. The analysis of chromosomal neighbourhoods was possible thanks to the invaluable “ortholog neighbourhood viewer” tool implemented at the IMG-JGI. Hundred and twenty-one chromosomal neighbours of the 33 RH50 genes (30 bacterial plus 3 *T. vaginalis* paralogs) in 31 genomes were analysed (Additional file 1: Table S4). To detect potential HGT events, out of 121 neighbours, 14 proteins (in 12 datasets) showed evidence of HGT events (Tables 2 and Additional file 1: Table S4). All phylogenies are shown in (Additional file 3: Trees T01-T12).

**Table 2:** Phylogenetic evidence of HGT in neighbouring genes

Species	IMG Locus_tag ^a^	IMG product name	Tree label ^b^	# taxa	# sites	Adjacent taxa in tree
*Methanomassiliicoccus luminyensis B10*	*missing (Mlum65)*	NADP oxidoreductase, coenzyme F420-dependent	T01	85	146	Alpha-, Deltaproteobacteria
*Ca.* Kuenenia stuttgartiensis	kustc0379	Unknown protein	T02	47	368	div. BRC1 bacterium; Gammaproteobacteria
*"*	kustc0382	Glutamate formimidoyltransferase	T03	85	484	Marine euryarchaeotes
*"*	kustc0383	Glutaredoxin-like protein	T04	39	74	Firmicutes
*"*	kustc0384	Permease of the major facilitator superfamily	T05	61	389	Deltaproteobacteria
*Planctomycetaceae bacterium* KSU-1	*missing (KSU_979)*	Indole-3-glycerol phosphate synthase	T06	74	238	Acidobacteria
*"*	*missing (KSU_981)*	Conserved hypothetical protein	T07	63	88	Deltaproteobacteria
*Ca.* Brocadia anammoxidans WQC04	*missing (Brocad1)*	Indole-3-glycerol phosphate synthase	see T06			
*"*	*missing (Brocad3)*	Predicted membrane protein	see T07			
*Nitrosomonas europaea* ATCC 19718	NE0446	3-demethylubiquinone-9-3-methyltransferase	T08	76	156	Gammaproteobacteria
*"*	NE0447	3-methyladenine DNA glycosylase I	T09	59	189	Gammaproteobacteria
*"*	NE0449	Aspartate and glutamate racemase	T10	45	238	Gammaproteobacteria
*Nitrosomonas sp.* AL212	NAL212_0966	Hypothetical protein	T11	51	141	Gammaproteobacteria
*Geobacter sp. M21*	GM21_1429	Diguanylate cyclase	T12	55	156	Gammaproteobacteria

Phylogenetic Bayesian inference was carried out in PhyloBayes, ML inference in RAxML and PhyML. Number of taxa and sites in the alignment are given. All BI analyses were run to convergence (maxdiff < 0.1 and eff. size >100). ML in RAxML used “-f a” option with 1,000 rapid-bootstrap replicates. ML in PHyML used SPR tree-space search strategy with 5 random starts + BioNJ. Prottest best-fitting model was LG + Г4 + F for T05, T07, T09, T10, datasets, LG + Г4 for all the others. See Additional file 1: Table S4 for full data

^a^When Integrated Microbial Genomes (IMG) locus tag was missing, an arbitrary one was chosen

^b^All trees in Additional file 3

The analysis of HGT in neighbouring genes did not lend support to any of the HGT trajectories revealed by the Rh50 phylogeny (Fig. 1), yet it did uncover alternative trajectories; this result is of interest as to the mode of evolution of RH50 in prokaryotes (see Discussion).

### The Amt phylogeny supports some of the HGT trajectories found in the Rh50 phylogeny

Additional evidence to test the HGT scenarios suggested by the phylogenetic analyses of RH50 genes and their chromosomal neighbours might be provided by the evolutionary history of AMT genes present in those prokaryotic genomes coding for RH50. The rationale behind such reasoning is that Rh50 and Amt belong to the same protein family and are also functionally related. Indeed, Rh50 has been shown to replace functionally Amt in *N. europaea* [17], and the two proteins may have complementary functions in the mosquito *Anopheles gambiae* [18]. This argues in favour of a linked evolutionary history whereby the functional interplay between the two proteins may be correlated to RH50/AMT duplication-transfer-loss events in different organisms.

The 31 genomes coding for RH50 genes also code for 49 AMTs (red diamonds in Additional file 2: Figure S3); notably, AMTs are absent in 12 genomes coding for RH50 (Table 1). The Amt phylogeny reveals the existence of several HGT events, many of which have been described in previous studies [25–27] and will not be dealt with here. Instead, the Amt phylogeny lends support to the potential HGT trajectories disclosed by the Rh50 phylogeny (Additional file 2: Figure S3 and Fig. 1). The topologies of different clades suggest a potential directional HGT from methanogens to anammox. More details are given in the legend of Additional file 2: Figure S3.

### Tree reconciliation analysis

**Fig. 2:**
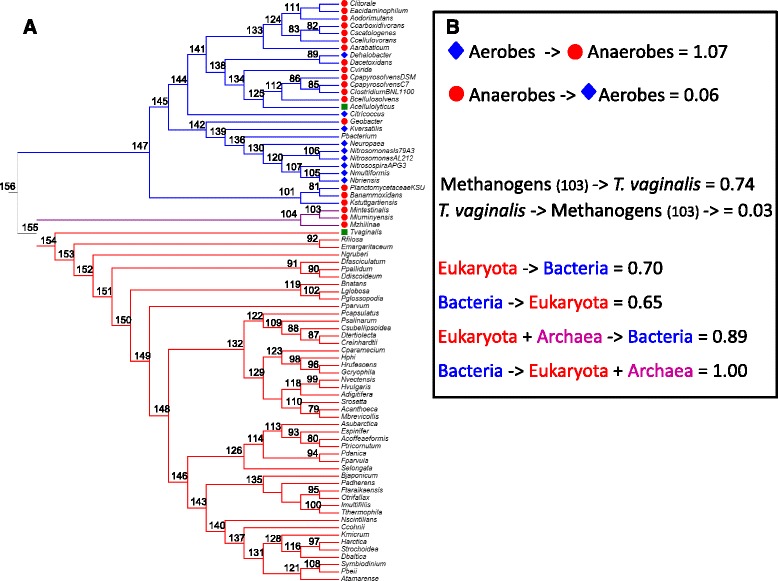
Tree reconciliation analysis. **a** Species tree cladogram. The root was arbitrarily placed between Bacteria and Eukaryota + Archaea. Branches are numbered according to the.uTs ALE file. **b** Sum of the observed frequencies of transfer from branches (numbered in **a**) leading to Eukaryota, Archaea and Bacteria occurring in 100 gene phylogenies sampled from a gene tree space of 39,504 trees. See Methods for further details. Aerobes were found to act as HGT donors to Anaerobes more often than the other way round. Branch 103, leading to two euryarchaeal methanogens (*M. intestinalis, M. luminyensis*), is found to be the preferential donor in the HGT involving *T. vaginalis*. Colour codes as in Fig. 1

The presence of RH50 genes in a minor fraction of bacterial and archaeal genomes, both at the Domain, Phylum and Genus levels, could be accounted for by a large number of independent gene-losses. However, this scenario is classically regarded as unlikely. The alternative and most parsimonious explanation is that prokaryotes have acquired RH50 genes via HGT from a eukaryotic donor. Here, the main purpose of tree reconciliation analysis was to shed light on the potential “origin” of RH50 genes, i.e. on the directionality of gene transfer between Bacteria on one side and Eukaryota or Eukaryota + Archaea on the other. No firm conclusion can be drawn (Fig. 2b, Additional file 4: Tables S6–S9).

Instead, the analyses suggest that (i) branch 103, leading to two euryarchaeal methanogens (*M. intestinalis, M. luminyensis*) is found to be the preferential donor in the HGT involving *T. vaginalis* in 0.74 of the gene trees (versus 0.03 in the opposite direction; see also Fig. 1); and (ii) the facultative *Acetivibrio cellulolyticus* might have been the HGT recipient from anaerobic Clostridiales (branch 86, Fig. 2a; Additional file 4: Table S5).

## Discussion

The evolutionary history of RH50 genes in prokaryotes is just beginning to be unveiled. To date, no documented evidence was available for the presence of RH50 genes in archaea, and only the Rh50 protein from the ammonia-oxidizing bacterium *N. europaea* (NeRh50) has been characterized as an ammonium permease [17].

In the present study, I show that HGT is the driving force in the evolution and spread of RH50 genes in prokaryotes. While the absolute number of genomes coding for RH50 is expected to rise as more will be sequenced, their phylogenetic distribution is likely to remain heavily skewed. An educated guess predicts that more representatives among the Firmicutes will be found. Another significant finding of this study is that RH50 and AMT genes coexist in a small number of prokaryotes (see below).

Given the available taxon sampling of bacterial and archaeal genomes, the results presented here suggest that HGT acted to spread RH50 yet among a restricted number of phyla and species; this formed a HGT exchange network whose main trajectories, as well as their relationship to ecological and metabolic niches, I have tried to elucidate here.

### RH50 and AMT evolution in prokaryotes: possible scenarios

AMT genes are ubiquitous in both bacterial and archaeal genomes in single- or multiple-copies (Tables 1 and Additional file 1: Tables S2–S3). Remarkably, when AMT is absent, in the vast majority of cases those genomes code for RH50 (Table 1; see also below). Given the pervasiveness of AMT genes in prokaryotes and the corresponding rarity of RH50, the likely conservation of their biochemical function as ammonium permeases and the evidence for non-orthologous displacement of Amt by Rh50 in *N. europaea* [17], the most parsimonious hypothesis is that a duplication event from an AMT ancestor is at the origin of the RH50 gene.

With the caveat of genome sequencing accuracy, the molecular phylogenies of Rh50 proteins and their Amt homologs and their phyletic patterns suggest three scenarios for the evolution of the AMT/RH50 gene family in prokaryotes analysed here. In the first scenario, genomes code only for AMT; this occurs in most Bacteria (e.g., 1–4 copies/genome in Planctomycetes, (Additional file 1: Table S3) and Archaea (e.g., 1–3 copies in methanogens, Additional file 1: Table S2). In the second scenario, RH50 (always in single-copy) and AMT (1–7 copies) coexist in 2 methanogens (*M. luminyensis* and *M. zhilinae*) and 17 Bacteria (Tables 1, Additional file 1: Tables S2–S3). This is likely to be a rare event, and it may be speculated that such coexistence might have adaptive and/or ecological relevance (see Conclusions). In the third scenario, also apparently rare, genomes code only for RH50 (AMT is absent). This is the case of 11 prokaryotic genomes: the euryarchaeal methanogen *Ca.* M. intestinalis, 6 ammonia-oxidizing bacteria (AOBs), and 4 Firmicutes (Table 1).

### “Origin” of the RH50 gene

Figure 1 shows that both eukaryotes and prokaryotes are largely monophyletic. If HGT had occurred between the two realms, the Rh50 phylogeny would support at best only a single HGT event. Alternatively, if prokaryotes had obtained their RH50 repeatedly from different eukaryote donor(s), they would be nested at different places within eukaryotes, which is not the case. An alternative scenario, the RH50 originating in prokaryotes, followed by extensive gene-loss in the majority of prokaryote branches seems less parsimonious. It might be argued that RH50 has been retained only in the few prokaryote lineages that “needed” it. This “adaptive” hypothesis may indeed hold in the case in anammox [31] and in AOBs. Yet, it is clearly contradicted by the phyletic distribution in archaeal methanogens (only 3 out of 39 genomes encode RH50), for all methanogens use ammonium as source and/or by-product (see below). The same reasoning applies to the phyletic distributions in the *Geobacter* genus (1 out of 8 genomes encode RH50; see above), in Actinobacteria (only *Citricoccous*/820), and Acidobacteria (only *Ca.* K. versatilis/14) (Additional file 1: Table S1).

Although the phylogenetic analyses are not conclusive to discriminate between a prokaryotic or eukaryotic origin of RH50, the results on the phylogenetic distribution of RH50 leaves open the possibility of a eukaryote donor (this scenario being neither proved nor disproved by the tree reconciliation analysis; Fig. 2b). Indeed, the frequency distribution of RH50 is proportionally exceedingly rare both within bacteria and archaea - 31 bacterial genomes code for a single-copy RH50 gene (27 are shown in Table 1), which remarkably is also present in 3 euryarchaeal methanogens (Tables 1 and Additional file 1: Table S2). Moreover, the RH50 phyletic patterns are also strikingly uneven among prokaryotes with respect to their corresponding Phylum and/or even Genus. Only 5 anammox among 28 Planctomycete genomes encode the RH50 gene (Additional file 1: Table S3), only six AOBs among 301 Betaproteobacteria, and only *Citricoccus* out of 820 Actinobacteria (Additional file 1: Table S1). Most notably, RH50 is present in the genome of *Geobacter* M21 but it is absent in seven other *Geobacter* genomes. Lastly, RH50 is found in 3 methanogens out of 215 euryarchaea and 39 euryarchaeal methanogens; this being the first documented evidence of RH50 in archaeal genomes.

### The case of *Trichomonas vaginalis*

Aiming at disentangling an entire HGT network would be illusory, for at best we can hope to find remnants or footprints of the HGT events which have taken place during evolutionary time, such events being characterised by different degrees of stability in the host genome. For example, the chromosomal regions upstream of the RH50 gene differed in *Clostridium papyrosolvens* C7 and *C. papyrosolvens* DSM2782 (not shown). Phylogenetic (tree inference and reconciliation) and phyletic pattern analyses identified several potential trajectories in the RH50 HGT network, one of which involves the parabasalian *Trichomonas vaginalis*.

The genome of *T. vaginalis* codes for three RH50 paralogs but lacks AMT genes. Among the nine Excavata genomes known to date, RH50 genes are found only in *T. vaginalis* and *Naegleria gruberi*. In the RH50 phylogeny (Fig. 1), while *N. gruberi* clusters with eukaryotes as expected, *T. vaginalis* is sister to methanogens. The strongly supported positioning of *T. vaginalis* in the phylogeny (Fig. 1) and the tree reconciliation analysis (Fig. 2b) support the acquisition of RH50 from a methanogen donor. Additional lines of evidence support this conclusion. In the genome of *T. vaginalis*, out of about 26,000 genes, only 65 have no introns [32], among which the RH50 paralogs. Hundred and fifty-two potential cases of prokaryote-to-eukaryote HGT candidates were detected in the genome of *T. vaginalis* [32], and two cases of HGT from methanogen donors were identified (see Additional file 3: trees TN095 and TN165 in [33]). In both of these genome-wide studies, the HGT of the RH50 gene was not detected. Finally, methanogens and *T. vaginalis* are in physical proximity in human mucosae [33].

The interesting cases of HGT involving methanogens, anammox, and *Acetohalobium arabaticum* are discussed in greater detail in the next two sections.

### RH50 in methanogens as an adaptation to the methylotrophic pathway

Methanogenesis is a form of anaerobic respiration and is carried out by euryarchaeal methanogens; they are a primary source of biogenic methane release in the atmosphere and are found in terrestrial, marine and freshwater sediments but also in the gastrointestinal tracts of mammals and insects. All methanogens are strictly anaerobic and belong to seven euryarchaeal orders, namely Methanococcales, Methanobacteriales, Methanopyrales, Methanomicrobiales, Methanocellales, Methanosarcinales, and Methanomassiliicoccales [34]. Methanogen orders show different specificities with respect to their substrate for methanogenesis (Additional file 1: Table S2). To generate methane, three main pathways are used: (i) hydrogenotrophic (from H_2_ reduction of CO_2_ or formate), (ii) acetoclastic (from acetate cleavage) and (iii) methylotrophic (from C1 compounds such as methanol and methylamines). Moreover, in general methanogens grow in syntrophic associations with fermentative bacteria producing methanogenic substrates [35].

The phyletic pattern analysis of RH50 and AMT genes in methanogens shows that only 3 out of 39 methanogen genomes code for RH50, *Methanomassiliicoccus luminyensis*, *Ca.* M. intestinalis (Methanomassiliicoccales) and *M. zhilinae* (Methanosarcinales), while all of them (save *Ca.* M. intestinalis) code for AMTs (Additional file 1: Table S2). In the genome of these three species, RH50 genes are neighbours of methyltransferases, key players in the methylotrophic pathway. Thus, it may be speculated that RH50 and methyltransferases may be co-regulated in the same operon to adapt to different growth conditions. This hypothesis is corroborated by the finding that in *Methanosarcina mazei* genes specific to the methylotrophic pathways are co-regulated in a substrate-dependent manner [36] and two AMT genes (together with nitrogenase and glutamine synthetase genes) are up-regulated under nitrogen-limiting conditions [37]. In the case of *Ca.* M. intestinalis, Rh50 protein may have functionally replaced the missing Amt, as in the case of the AOB *N. europaea* [17].

In methylotrophic methanogens, ammonium is known to play two roles: beneficial (as a required substrate) and detrimental (as a toxic compound). In the methylotrophic pathway, ammonium is released during demethylation of monomethylamine. Interestingly, genes involved in methanogenesis are in close chromosomal association with ammonia permeases - both RH50 and AMT in *M. luminyensis;* RH50 in *Ca.* M. intestinalis RH50 (lacks AMT), but not with AMT in *Ca.* M. alvus (lacks RH50) (not shown). Borrel and co-workers observed this association in the three Methanomassiliicoccales, and suggested that “dedicated transporters” may be involved in the export of ammonium [34]. Moreover, methylotrophic methanogens can use ammonia as nitrogen source for amino acids synthesis [38]; its potential sources being a direct uptake from the environment by ammonia permeases (likely Rh50 and/or Amt) and the intracellular ammonium being released during demethylation of monomethylamine.

In a wide range of pH, NH_3_/NH_4_^+^ were reported to inhibit methanogenesis [39], NH_3_ being more toxic than NH_4_^+^ ([40] and references therein). It may be hypothesised that ammonia permeases (Rh50 and/or Amt) might be required to maintain ammonia homeostasis. A telling example is provided by *M. zhilinae* (*alias Methanohalophilus zhilinae*). Its optimal growth occurs at pH 9.2 and 45 °C [41], conditions in which the NH_3_/NH_4_^+^ equilibrium is shifted toward NH_3_. It is possible that in *M. zhilinae* the excretion of toxic NH_3_ could be facilitated by Rh50 and/or Amt, similar to the role suggested for the Rh50 proteins in the tilapia fish living in the alkaline waters (pH ~ 10) of Lake Magadi [42].

In conclusion, there may be an adaptive correlation between RH50 acquisitions via HGT (and its maintenance) and the use of the methylotrophic pathway in the hosting organisms. Indeed, some of the products of the methylotrophic pathway, CH_4_, CO_2_, and NH_3_, could be substrates for a non-specific Rh50 gas permease, if an increase in diffusion rate through the cell membrane(s) were needed (see Introduction). The donor species (eukaryote or prokaryote) in the HGT event that allowed the three methanogens to acquire RH50 remains unknown, yet their sister group relationship with the acetogenic *Acetohalobium arabaticum* in the Rh50 tree (Fig. 1) may suggest a possible scenario.

### Syntrophic associations likely favoured HGT: the cases of *A. arabaticum* and anammox

*Acetohalobium arabaticum* belongs to the Firmicute order of Halaneorobiales. The Rh50 phylogeny indicates a potential HGT involving *A. arabaticum* and the Actinobacteria *Citricoccus*, both being sister to the methanogens (Fig. 1). *A. arabaticum* is a fermentative methylotrophic anaerobe that produces acetate, mono-, di- and trimethylamines [43], which are substrates in methanogenic pathways. Among bacteria, only *A. arabaticum* encodes the three mono-, di-, and trimethylamine transferases [44]. The gene cassette required to biosynthesize and decode UAG codons as pyrrolysine (Pyl) is encoded in the genomes of 35 prokaryotes, namely 16 Euryarchaeota (including *M. zhilinae*, *M. luminyensis*, and *Ca.* M. intestinalis), 16 Firmicutes (including *A. arabaticum* and *D. acetoxidans*) and 3 Deltaproteobacteria (not shown). Some of them were described previously [44]. *A. arabaticum* likely acquired both pyrrolysyl-tRNA synthetase and methylamime transferases genes via HGT from euryarchaeal methanogens [44]. *A. arabaticum* may live in syntrophy with methanogenic euryarchaea, their physical proximity having likely facilitated HGT events.

In the anaerobic ammonia oxidizing reaction, equimolar amounts of ammonium and nitrite are converted into molecular N_2_ gas. Three other aerobic steps in the biological nitrification process are performed by ammonia-oxidizing bacteria (AOB) and archaea (AOA) and nitrite-oxidizing bacteria (NOB). Incidentally, neither AOA nor NOB encode for RH50 genes (not shown). Likely due to their syntrophic relationships (see also below), ecological niches are known to be shared between NOB and AOA, NOB and AOB [45], as well as between anammox and methanogens [46] and between anammox and AOBs [47]. Another peculiar feature of anammox organisms is the presence of a membrane-bound compartment in the cell, the anammoxosome, in which the anammox reaction is believed to take place [48]. The membrane of the anammoxosome contains ladderane lipids which confer high density and low permeability to the membrane thereby preventing passive diffusion of small molecular intermediates during their slow life cycle [49]. The Rh50 permease is expected to enhance NH_3_ flux across dense membranes [14]; therefore, the acquisition of an ammonia permease via HGT, possibly from a methanogen donor, may be regarded as adaptively advantageous to anammox. Along the same line, for ammonium is a limiting factor for anammox bacteria in oxygen minimum zones, it has been suggested that the expression of high-affinity ammonium transporters (Amt) might provide a selective advantage to them [31].

### HGT-driven evolution may take place at boundary layers

HGT is known to occur more frequently among closely related species. Likewise, organisms sharing the same ecological niche are more prone to HGT, yet inter-habitat events do occur ([11] for a review), and barriers to HGT, such as sequence divergence and genomic GC content, can be bypassed [50]. In addition, the importance of environmental selection and the existence of ecologically determined gene-transfer networks enabling sharing of niche-adaptive genes has been proposed [51].

Here, I show that the RH50 HGT network is characterised by the fact that it crosses oxygen boundaries (classically regarded as a barrier), by the rarity of genetic transfers, and by an extremely narrow taxonomic distribution of HGT events among prokaryotes.

A case in point concerns the aerobe *Ca.* K. versatilis; in the Rh50 tree it clusters within one of the two clades of Clostridiales, which comprise typically anaerobic species (Fig. 1). Other remarkable examples of potential cross-barrier point in the RH50 HGT network are (i) the convincing trend that favours HGT of RH50 from aerobe to anaerobe organisms, in particular from the branch 139 to *Anaerovorax odorimutans*, and from *Citricoccus* to branch 104 of methanogens (Fig. 2b; Additional file 4: Table S10), and (ii) the exchange between the facultative *Acetivibrio cellulolyticus* and other anaerobic Clostridiales (branch 86, Fig. 2a, Additional file 4: Table S5).

Physical and metabolic interactions between the species forming the RH50 HGT network have been reported, also in the form of syntrophic associations (see above). Interestingly, from the interior to the exterior layers of a sludge granule of a bioreactor, the microbial community is composed of methanogens, anammox and AOB (see Fig. 4 in [46]) and share an ecological niche known as oxygen minimum zone [33, 51]. Moreover, two putative Amt proteins, expressed at the cell membrane of the anammox *Scalindua profunda*, might be involved in ammonium scavenging [31], which in turn could provide N_2_ source to AOBs and/or methanogens.

To sum up, the main trajectories of the RH50 HGT network point to a still much unexplored ecological niche where HGT might be enhanced, namely the oxic-anoxic boundary layers, such as the oxygen minimum zones (OMZ). Indeed, the HGT of periplasmic nitrite oxidoreductase genes between anammox and the aerobic nitrite-oxidizing bacteria *Nitrospina* has been reported to occur in marine OMZ [44].

## Conclusions

The phylogenetic analyses presented here, and data from the literature, identify several potential trajectories in the RH50 HGT evolutionary network in prokaryotes, a striking feature of which is that it seems to cross oxygen boundaries. The most “informative” nodes of the network are methanogens, *Acetohalobium arabaticum*, anaerobic and aerobic ammonia-oxidizing bacteria (anammox and AOB). Their relationships suggest that (i) syntrophic relationships play a major role in the development of the network, and (ii) oxygen minimum zones -and boundary layers in general — might be an ecological niche of crucial importance for HGT-driven evolution.

The present findings pave the way to two types of experimental investigations. The presence of RH50 in such a restricted spectrum of archaea is puzzling. RH50 is found in both *M. luminyensis* and *Ca.* M. intestinalis but the latter lacks AMT; both share the same niche in animal digestive tracts. Detailed functional and structural studies of these two Rh50 proteins might reveal the nature and origin of adaptive changes. Similar insights might be gained by comparative structure/function analysis of the Rh50 and Amt proteins from organisms in which they coexist, namely anammox, and the methanogens *M. luminyensis* and *M. zhilinae*. Finally, genome and transcriptome comparisons between organisms inhabiting oxygen minimum zones will clarify the role of this ecological niche in promoting HGT-driven evolution.

## Methods

### Datasets

Rh50 homologs in prokaryotes were identified by blastp-searching the Integrated Microbial Genomes resource (IMG at the Joint Genome Institute, JGI), and the NCBI GenBank non-redundant protein database (July 2014). In all prokaryote genomes coding for RH50 genes, AMT homologs were identified by blastp-searches against genome-specific databases at IMG-JGI and/or NCBI. A blastp search, with default settings, is sufficient to identify any homolog within both Rh50 and Amt families.

As for prokaryotic Rh50, of the 34 homologs identified, four were not retained, namely those present in “Candidatus Kuenenia stuttgartiensis” RU-1 and CH-1 isolates (being included in small scaffolds) and the two homologs in Dehalobacter sp. CF and sp. UNSWDHB (for they share 100% amino acid identity and the same chromosomal neighbourhood with Dehalobacter sp. 11DCA RH50; see below). Therefore, thirty Rh50 prokaryotic proteins were included in the dataset: 7 Proteobacteria (6 Beta- and 1 Deltaproteobacteria), 3 Plancomycetes, 1 Acidobacteria, 15 Firmicutes, 1 Actinobacteria and 3 Euryarchaeota (Table 1).

### Multiple sequence alignment (MSA) and evolutionary model selection

Protein transmembrane (TM) topologies were predicted using TMHMM [52] at http://www.cbs.dtu.dk/services/TMHMM/. MSA for Rh50 and other TM proteins coded by RH50 chromosomal neighbours was carried out in TM-Coffee [53]. Praline™ [54] was used for Amt proteins, because of the large dataset size, at http://www.ibi.vu.nl/programs/pralinewww/. MSA for non-TM proteins, and for those proteins with only one small TM (with respect to protein length) at the N-ter or C-ter end, was carried out in ClustalO [55] or Muscle [56], as implemented in SeaView v. 4 [57] (see Additional file 1: Table S4). The confidence of aligned residues was assessed using the TCS index [58]; only columns with TCS index ≥6 and ≥7 (on a 0–9 scale) were retained for Amt and Rh50 alignments, respectively. In the alignments of the 26 RH50-neighbours datasets, the TCS threshold was ≥6 (save for eight instances). All MSAs were further refined manually in SeaView. Alignments before and after trimming are provided in (Additional files 5, 6 and 7).

ProtTest v3.2 [59] was used to assess the best model fitting the data using a ML tree as starting topology and choice was based on a majority-rule consensus of the implemented statistics. Models with proportion of invariant sites were excluded as rate heterogeneity is accounted for by the gamma shape parameter. In order to compare the site-homogeneous LG to the site-heterogeneous CATGTR mixture model, a cross-validation procedure was carried out in PhyloBayes v. 3.3 [60]. The procedure is computationally intensive and briefly consists in randomly splitting the dataset in learning set (9/10^th^) and test set (1/10^th^). Model parameters are then estimated on the learning set for each model (11,000 cycles; the first 1,000 being discarded as “burnin”) and used to calculate the cross-validation log-likelihood scores of the test set, averaged over the ten replicates (refer to PhyloBayes manual).

### Molecular phylogeny

Both Maximum-likelihood (ML) and Bayesian inference (BI) methods were used. ML inference was performed in RAxML v. 8.0.19 [61], IQ-TREE v. 1.0.1 [62] and PhyML v3 (build 20120412) [63]. Except for the first ML analysis of Rh50-neighbouring genes datasets (see below), RAxML analyses used the “−f a” option with 1,000 bootstrap pseudo-replicates. IQ-TREE was run using 10,000 bootstrap replicates. Tree-topology searches in PhyML were conducted applying Subtree Pruning and Regrafting moves (starting from 5 random trees and BioNJ tree). Bayesian inference was carried out in PhyloBayes (under LG + Γ4 and CATGTR + Γ4 models). Two independent chains were run and their bipartitions were compared after discarding 20% of cycles as “burn-in” and sampling each 10th cycle. All analyses were run till convergence: maximal difference (maxdiff) observed between bipartition-frequencies of runs was always < 0.1, and minimum effective size always > 100 (refer to PhyloBayes manual).

Branch support values were: rapid bootstrap pseudo-replicates (RBS, [64]) in RAxML, ultrafast-bootstrap approximation (UFBoot, [65]) in IQ-TREE, aBayes and SH-aLRT [66] in PhyML, Bayesian posterior probabilities, PP, in PhyloBayes. Tree editing and annotation were performed in MEGA v. 6 [67]. As a cautionary note, in HGT studies, taxon sampling needs be expanded as much as possible, thereby leading to a low ratio between sites in the multiple alignment and number of taxa in single-gene phylogenies. This may translate into low branch-support values that may not reach the commonly accepted significance thresholds (i.e., ≥70% for ML non-parametric bootstrap and ≥0.95 for BI posterior probabilities).

Tree topology testing was carried out in CONSEL [68] and RAxML (ELW test, [69]). Per-site log-likelihoods were calculated in RAxML under the LG + Γ4 + F model.

It is well known that compositional heterogeneity may lead to biased phylogenetic inference [70]. The compositional homogeneity of the aligned residues in the Rh50_prok_clade was assessed using the statistic implemented in PhyloBayes as well as principal component analysis (not shown). Rh50 deviated compositionally in six taxa, *viz. T. vaginalis* 428240, *Planctomycetaceae KSU-1*, *Nitrosospira multiformis*, *Nitrosospira APG3*, *Geobacter*, and *Citricoccus*. The first four taxa showed the expected topology in the phylogeny, indicative of strong phylogenetic signal. The outlier positioning of *Geobacter* varied, depending on the aligned residues.

### Tree reconciliation analysis

Briefly, tree reconciliation analysis aims at reconstructing a gene phylogeny taking into account potential events of gene duplication, transfer and loss and tries to draw evolutionary scenarios using a “consensus” species tree as a reference [71]. The Amalgamated Likelihood Estimation (ALE) method, a gene tree-aware approach based on probabilistic models that include parameters for gene duplication, loss and transfer, was used for tree reconciliation [72]. The analysis was carried out in ALEml_undated v0.4 which handles undated species trees [71]. Species tree was based on small subunit rRNA sequences retrieved from SILVA [73] and aligned in SINA (gaps were excluded) [74]. Model selection and ML inference (under GTR + I + Γ4) were carried out in IQ-TREE. A gene tree space of 39,504 trees was derived from the two chains in PhyloBayes used in Fig. 1. Transfer frequency values (Fig. 2b, Additional file 4: Tables S5–S10) were obtained summing up the transfer frequencies between pair of branches (and they may obviously exceed 1), therefore they should not be confused with probabilities and should be interpreted as a trend in the data.

### Datasets and phylogenetic analyses of RH50 chromosomal neighbouring genes

Hundred and twenty-one chromosomal neighbours of the 33 RH50 genes in 31 genomes (3 paralogs in *T. vaginalis*) were analysed (for complete list see Additional file 1: Table S4). Of those, 91 proteins-coding genes were suited for phylogenetic analysis. Homologous protein datasets were assembled using the following procedure. For each homologous set, on average 200–300 sequences were gathered by pooling the pre-computed “Top IMG homolog Hits” (from JGI-IMG; [75]), the homologs identified by blastp searches against NCBI and Pfam protein databases and the pre-computed homologous set of the corresponding INTERPRO entry. This preliminary dataset was purged of redundancy using Cd-hit v. 4.6 [76] with either 90% or 95% cut-off. Then, a first ML analysis, under an arbitrary LG + Γ4 + F model, was carried out on the non-redundant datasets in RAxML (“-f a” option and 300 RBS replicates) and PhyML (“best NNI/SPR” tree-space searching strategy; aLRT branch-support). Moreover, to improve tree resolution and branch support, “rogue taxa” (i.e. unstable taxa in the phylogeny), were removed, where judged necessary, using the RogueNaRok algorithm [77]. Thirty-one proteins (in 26 datasets, for in some instances more than one neighbour was present in the same MSA) showed evidence of HGT and therefore their phylogenies were re-analysed by Bayesian and ML inferences as described above.

The genome of *Ca.* Brocadia anammoxidans WQC04 was withdrawn from the IMG database on March 2014, because of poor quality (though it can still be accessed at NCBI, taxid: 174632). However, given that in all phylogenies inferred here (i.e., Rh50, Amt and Rh50-neighbours) Brocadia clustered with other anammox, thereby reinforcing that clade, I considered those sequences reliable and therefore included them in the datasets.

## Additional files

Additional file 1: Table S1. Phyletic pattern of RH50 homologs in bacterial and archaeal genomes. **Table S2.** Taxonomic distribution of RH50 and AMT homologs in euryarchaeal methanogens. **Table S3.** Taxonomic distribution of RH50 and AMT homologs in Planctomycetes and *Geobacter* Genus. **Table S4.** The dataset of 121 RH50 chromosomal neighbours. (XLSX 127 kb)
Additional file 2: Figure S1. Full phylogeny of Rh50 proteins. **Figure S2.** Phylogeny of Proteobacteria bacterium. **Figure S3.** Full phylogeny of Amt proteins. **Figure S4.** Tree-topology test. (PDF 1490 kb)
Additional file 3: Supplementary Trees. Phylogenetic evidence for HGT in chromosomal neighbours of Rh50. (PDF 3393 kb)
Additional file 4: Table S5. HGT frequencies among prokaryotes. **Table S6.** HGT frequencies from Bacteria to Eukaryota HGT. **Table S7.** HGT frequencies from Eukaryota to Bacteria. **Table S8.** HGT frequencies from Bacteria to Eukaryota + Archaea. **Table S9.** HGT frequencies from Eukaryota + Archaea to Bacteria. **Table S10.** HGT frequencies between Aerobes and Anaerobes. (XLSX 20 kb)
Additional file 5: Rh50 protein dataset (90 sequences). (DOCX 53 kb)
Additional file 6: Multiple sequence alignment of Rh50 protein dataset (before trimming). (PHY 339 kb)
Additional file 7: Multiple sequence alignment of Rh50 protein dataset (after trimming). (PHY 38 kb)

## Abbreviations

BLAST: Basic local alignment search tool
NCBI: National center for biotechnology Information
