# Supplementary material for: Horizontal gene transfer drives the evolution of Rh50 permeases in prokaryotes
Source: BMC Evol Biol. 2017 Jan 3;17:2. doi: 10.1186/s12862-016-0850-6 (PMC5209957; doi:10.1186/s12862-016-0850-6)
Supplement: Additional file 2: Figure S1. — Full phylogeny of Rh50 proteins. Figure S2. Phylogeny of Proteobacteria bacterium. Figure S3. Full phylogeny of Amt proteins. Figure S4. Tree-topology test. (PDF 1490 kb) [file 12862_2016_850_MOESM2_ESM.pdf]

Legends of Supplementary Figures S1-S4

Fig. S1. Full phylogeny of Rh50 proteins (90 taxa, 252 sites). Bayesian majority-rule consensus of 1,076 trees obtained under the LG+Γ4 mixture model in PhyloBayes. Two independent chains were run for 13,765 cycles, until chains reached convergence (maxdiff=0.0900641, effective size>100), discarding 20% of cycles as "burn-in" and sampling each 10th cycle. Black dots at relevant nodes indicate that the corresponding branch was supported by Bayesian posterior probabilities ≥ 0.95. Species oxygen requirements are denoted as follows: anaerobic (red dot), facultative (green square), aerobic (blue diamond). *Proteobacteria bacterium* JGI 0000113-E04 is highlighted in red. The scale-bar indicates the estimated number of substitutions per site.

Fig. S2. Bayesian phylogeny of *Proteobacteria bacterium* JGI 0000113-E04 beta-galactosidase/beta-glucuronidase (D341DRAFT_01804). The phylogeny was inferred in PhyloBayes under the CATGTR +Γ4 model (maxdiff = 0.16515; eff. size > 100). Bayesian posterior probabilities ≥ 0.95 are indicated by dots at nodes. D341DRAFT_01804 is sister to the choanoflagellate *Monosiga* (red-dot node). The scale-bar indicates the estimated number of substitutions per site.

Fig. S3. Rooted phylogeny of Amt proteins (229 taxa, 316 sites). Maximum likelihood tree under the LG+Γ4+F model in RAxML. The Amt2 taxon sampling included representative sequences from previous studies on HGT in the AMT family [25-27]. ML inference showed that AMTs from RH50-coding genomes all belonged to the Amt2 family. Therefore, in the final dataset only 7 Amt1 sequences were retained, as outgroup, to root the Amt2 tree. MSA was carried out in Praline™ and only columns with TCS ≥6 were retained (see Methods). The best-fitting model in ProtTest was LG+Γ4+F. ML inference was performed in RAxML and IQ-TREE. BI (under LG+Γ4) did not reach convergence (even after 12,000 cycles) likely due to the insufficient number of sites in the MSA with respect to the number of sequences. Branch support values relevant for discussion are given and displayed in the following order: Bayesian posterior probabilities, RBS (1,000 bootstrap replicates in RAxML), and UFBoot (10,000 bootstrap replicates in IQ-TREE). The 49 AMTs present in RH50-coding genomes are denoted by red diamonds. Branches colour-codes are: methanogens and other Euryarchaeota (blue), anammox (red), Planctomycetes (orange), Firmicutes (purple). Anammox are grouped in four clades. In Clades A and B, there is evidence of HGT between methanogens, anammox and Firmicutes. *Proteobacteria bacterium* JGI 0000113-E04 (black dot). The scale-bar indicates the estimated number of substitutions per site.
The anammox AMTs form 4 clusters (anammox1-4). Anammox cluster3 is sister to other Planctomycetes. Instead, the topologies of the two anammox clusters are informative; anammox cluster1 is nested within

archaeota comprising also methanogens (Clade A), and anammox cluster2 is nested within methanogens (Clade B). These topologies suggest a potential directional HGT from methanogens to anammox.
One of the Amt paralogs of *Geobacter M21* is sister to *A. cellulolyticus* and both lie in Clade A; the other *Geobacter* Amt is sister to three methanogens. *C. papyrosolvens* and the second Amt from *A. cellulolyticus* lie in Clade B. Overall, these data lend support to potential HGT trajectories already hinted by the Rh50 phylogeny, namely those between anammox and methanogens (Clades A and B), *Geobacter* and Firmicutes (Clade A), Firmicutes and methanogens (Clade B).

Fig. S4. Topology testing for the RH50_prok_meth clade.
In the Rh50 phylogeny, two competing topologies for the RH50_prok_meth clade were found: either as sister to the anammox (Tree1; red-dot node) or as basal to the Rh50_prok clade (Tree2; blue-dot node). Topology tests were carried out on a subset of 20 amino acids sequences. Per-site log-likelihoods were calculated in RAxML under the LG+Γ4+F model; these were used to perform the ELW test (as implemented in RAxML) and were also analysed in CONSEL. All tests supported Tree1 topology.

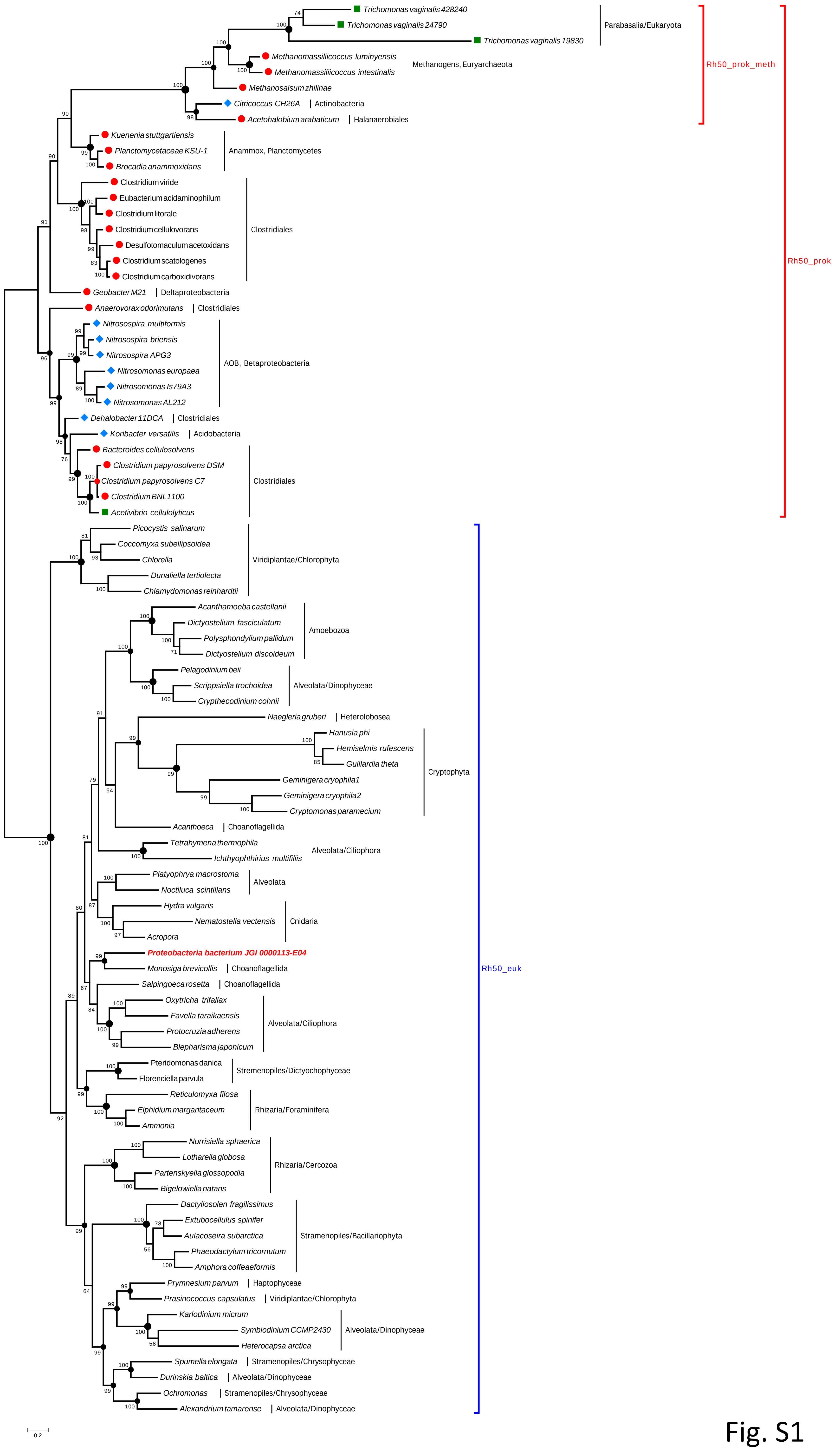
Trichomonas vaginalis 428240
Trichomonas vaginalis 24790
Trichomonas vaginalis 19830
Parabasalia/Eukaryota
Rh50_prok_meth
Methanomassiliicoccus luminyensis
Methanomassiliicoccus intestinalis
Methanogens, Euryarchaeota
Methanosalsum zhilinae
Citricoccus CH26A
Actinobacteria
Acetohalobium arabaticum
Halanaerobiales
Kuenenia stuttgartiensis
Planctomycetaceae KSU-1
Brocadia anammoxidans
Anammox, Planctomycetes
Clostridium viride
Eubacterium acidaminophilum
Clostridium litorale
Clostridium cellulovorans
Desulfotomaculum acetoxidans
Clostridium scatologenes
Clostridium carboxidivorans
Clostridiales
Rh50_prok
Geobacter M21
Deltaproteobacteria
Anaerovorax odorimutans
Clostridiales
Nitrosospira multiformis
Nitrosospira briensis
Nitrosospira APG3
Nitrosomonas europaea
Nitrosomonas Is79A3
Nitrosomonas AL212
AOB, Betaproteobacteria
Dehalobacter 11DCA
Clostridiales
Koribacter versatilis
Acidobacteria
Bacteroides cellulosolvens
Clostridium papyrosolvens DSM
Clostridium papyrosolvens C7
Clostridium BNL1100
Acetivibrio cellulolyticus
Clostridiales
Picocystis salinarum
Coccomyxa subellipsoidea
Chlorella
Dunaliella tertiolecta
Chlamydomonas reinhardtii
Viridiplantae/Chlorophyta
Acanthamoeba castellanii
Dictyostelium fasciculatum
Polysphondylium pallidum
Dictyostelium discoideum
Amoebozoa
Pelagodinium beii
Scrippsiella trochoidea
Crypthecodinium cohnii
Alveolata/Dinophyceae
Naegleria gruberi
Heterolobosea
Hanusia phi
Hemiselmis rufescens
Guillardia theta
Geminigera cryophila1
Geminigera cryophila2
Cryptomonas paramecium
Cryptophyta
Acanthoeca
Choanoflagellida
Tetrahymena thermophila
Ichthyophthirius multifiliis
Alveolata/Ciliophora
Platyophrya macrostoma
Noctiluca scintillans
Alveolata
Hydra vulgaris
Nematostella vectensis
Acropora
Cnidaria
Proteobacteria bacterium JGI 0000113-E04
Monosiga brevicollis
Choanoflagellida
Salpingoeca rosetta
Choanoflagellida
Rh50_euk
Oxytricha trifallax
Favella taraikaensis
Protocruzia adherens
Blepharisma japonicum
Alveolata/Ciliophora
Pteridomonas danica
Florenciella parvula
Stremenopiles/Dictyochophyceae
Reticulomyxa filosa
Elphidium margaritaceum
Ammonia
Rhizaria/Foraminifera
Norrisiella sphaerica
Lotharella globosa
Partenskyella glossopodia
Bigelowiella natans
Rhizaria/Cercozoa
Dactyliosolen fragilissimus
Extubocellulus spinifer
Aulacoseira subarctica
Phaeodactylum tricornutum
Amphora coffeaeformis
Stramenopiles/Bacillariophyta
Prymnesium parvum
Haptophyceae
Prasinococcus capsulatus
Viridiplantae/Chlorophyta
Karlodinium micrum
Symbiodinium CCMP2430
Heterocapsa arctica
Alveolata/Dinophyceae
Spumella elongata
Stramenopiles/Chrysophyceae
Durinskia baltica
Alveolata/Dinophyceae
Ochromonas
Stramenopiles/Chrysophyceae
Alexandrium tamarense
Alveolata/Dinophyceae
0.2

Fig. S1

Fig. S2

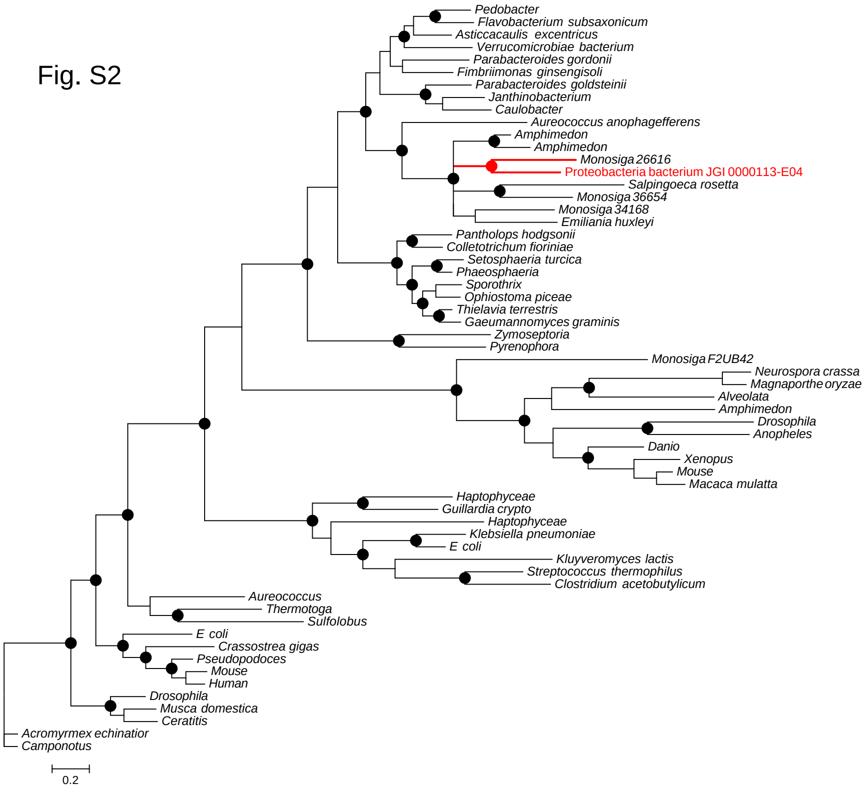
Pedobacter
Flavobacterium subsaxonicum
Asticcacaulis excentricus
Verrucomicrobiae bacterium
Parabacteroides gordonii
Fimbriimonas ginsengisoli
Parabacteroides goldsteinii
Janthinobacterium
Caulobacter
Aureococcus anophagefferens
Amphimedon
Amphimedon
Monosiga 26616
Proteobacteria bacterium JGI 0000113-E04
Salpingoeca rosetta
Monosiga 36654
Monosiga 34168
Emiliania huxleyi
Pantholops hodgsonii
Colletotrichum fioriniae
Setosphaeria turcica
Phaeosphaeria
Sporothrix
Ophiostoma piceae
Thielavia terrestris
Gaeumannomyces graminis
Zymoseptoria
Pyrenophora
Monosiga F2UB42
Neurospora crassa
Magnaporthe oryzae
Alveolata
Amphimedon
Drosophila
Anopheles
Danio
Xenopus
Mouse
Macaca mulatta
Haptophyceae
Guillardia crypto
Haptophyceae
Klebsiella pneumoniae
E coli
Kluyveromyces lactis
Streptococcus thermophilus
Clostridium acetobutylicum
Aureococcus
Thermotoga
Sulfolobus
E coli
Crassostrea gigas
Pseudopodoces
Mouse
Human
Drosophila
Musca domestica
Ceratitis
Acromyrmex echinatior
Camponotus
0.2

Fig S3

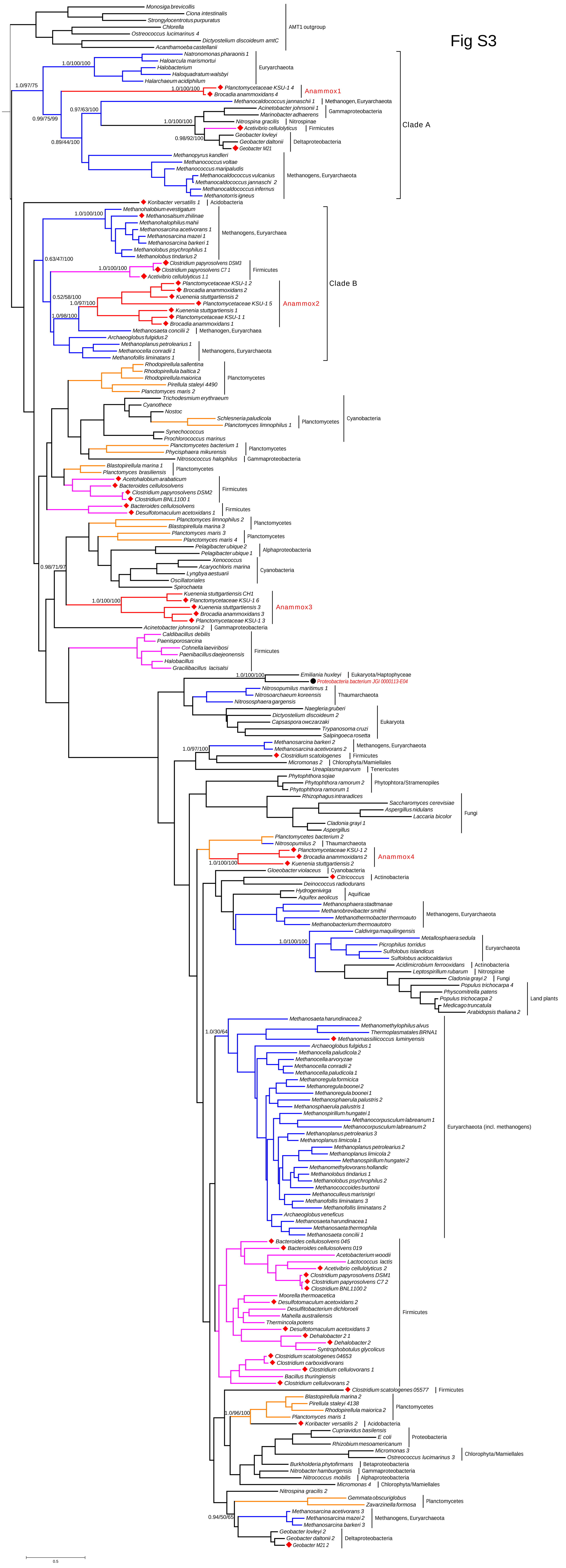
AMT1 outgroup
Clade A
Clade B
Anammox1
Anammox2
Anammox3
Anammox4
Euryarchaeota (incl. methanogens)
Firmicutes
Planctomycetes
Land plants

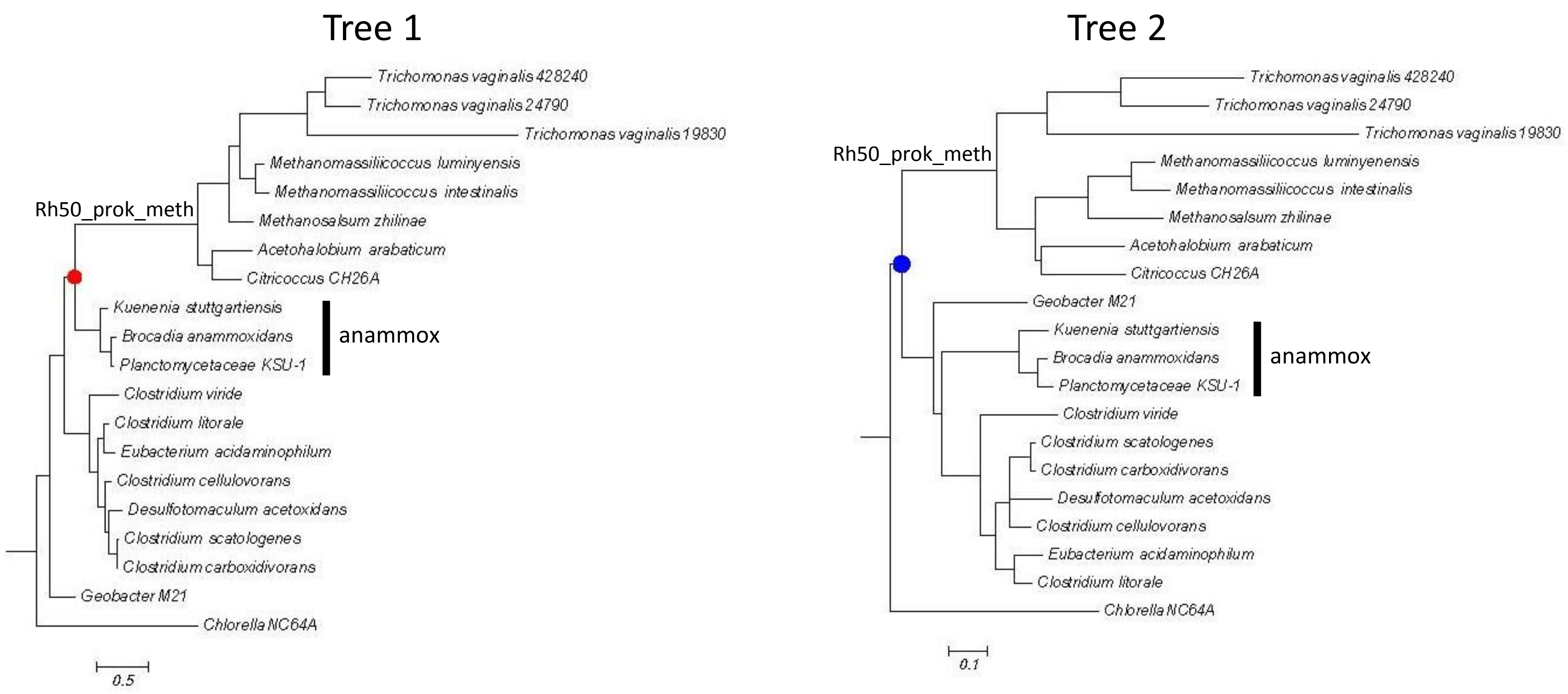

	rank	item	obs	au	np	|	bp	pp	kh	sh	wkh	wsh
**Tree1**	1	1	-34.6	**0.998**	**0.997**	**|**	**0.997**	**1.000**	**0.992**	**0.992**	**0.992**	**0.992**
Tree2	2	2	34.6	0.002	0.003	|	0.003	9e-16	0.008	0.008	0.008	0.008

CONSEL

	PP
**Tree1**	**0.997325**
Tree2	0.002675

ELW (RAxML)

Fig. S4
