# Supplementary material for: Horizontal gene transfer drives the evolution of Rh50 permeases in prokaryotes
Source: BMC Evol Biol. 2017 Jan 3;17:2. doi: 10.1186/s12862-016-0850-6 (PMC5209957; doi:10.1186/s12862-016-0850-6)
Supplement: Additional file 3: — Supplementary Trees. Phylogenetic evidence for HGT in chromosomal neighbours of Rh50. (PDF 3393 kb) [file 12862_2016_850_MOESM3_ESM.pdf]

Supplementary Trees. Phylogenetic evidence for HGT in chromosomal neighbours of RH50.

Hundred and twenty-one chromosomal neighbours of the 33 RH50 genes (30 bacterial plus 3 *T. vaginalis* paralogs) in 31 genomes were analysed (for complete list see Table S4). The chromosomal neighbourhoods of the RH50 homologs in *Clostridium papyrosolvens* C7 and *C. papyrosolvens* DSM2782 differed (not shown); therefore both isolates were retained in the dataset. To detect potential HGT events, out of 121 neighbours, 91 protein datasets could be analysed by ML inference in RAxML and PhyML (see Methods). The phylogeny of 14 chromosomal neighbours of RH50 (in 12 datasets) was analysed by BI and ML methods (Table 2). This file contains 12 Bayesian majority-rule consensus trees (T01-T12; two neighbours are included in T06 and T07). Bayesian inference was carried out in PhyloBayes (under LG+Γ4), ML inference (under the Prottest best-fitting model) in RAxML (using "–f a" option and 1,000 rapid bootstrap replicates) and PhyML (SPR moves starting from 5 random trees and BioNJ tree). Only branch support values relevant for the discussion are given and displayed in the following order: Bayesian posterior probabilities PP (PhyloBayes)/SH-aLRT (PhyML)/RBS (RAxML) (see Methods). In each phylogenetic tree information is given about gene IMG product name, number of taxa and sites, and PhyloBayes maxdiff convergence diagnostic parameter. BI was always run to convergence (maxdiff <0.1; effective size >100) discarding 20% of cycles as "burn-in" and sampling each 10th cycle. Leaf label identifies Genus (followed by IMG gene ID or NCBI accession). RH50 neighbours are indicated by a red dot at the branch tip. Black dots at nodes indicate that the corresponding branch was supported by PP ≥ 0.95. Scale bar indicates the estimated number of substitutions per site.

Each tree can be viewed by clicking the corresponding hyperlink in the table below.

Trees list

T01	T02	T03	T04	T05	T06	T07	T08	T09	T10
T11	T12								

T01 [Back to trees-list]

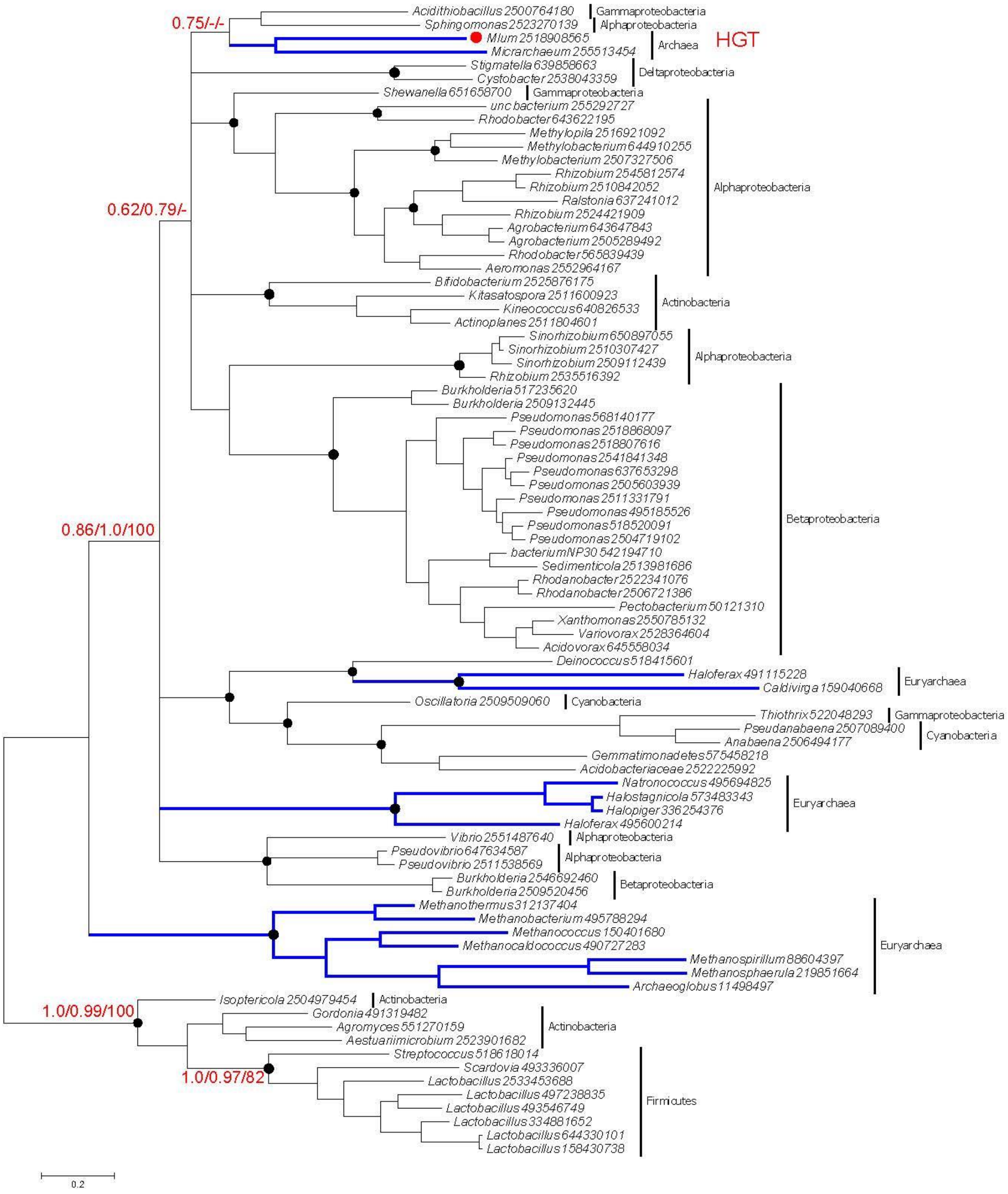

Species: Methanomassiliicoccus luminyensis B10

IMG product name: NADP oxidoreductase, coenzyme F420-dependent

taxa: 85

sites: 146

PhyloBayes BI maxdiff = 0.0995885

HGT: Gammaproteobacteria, Alphaproteobacteria <-> Archaea

T02 Back to trees-list

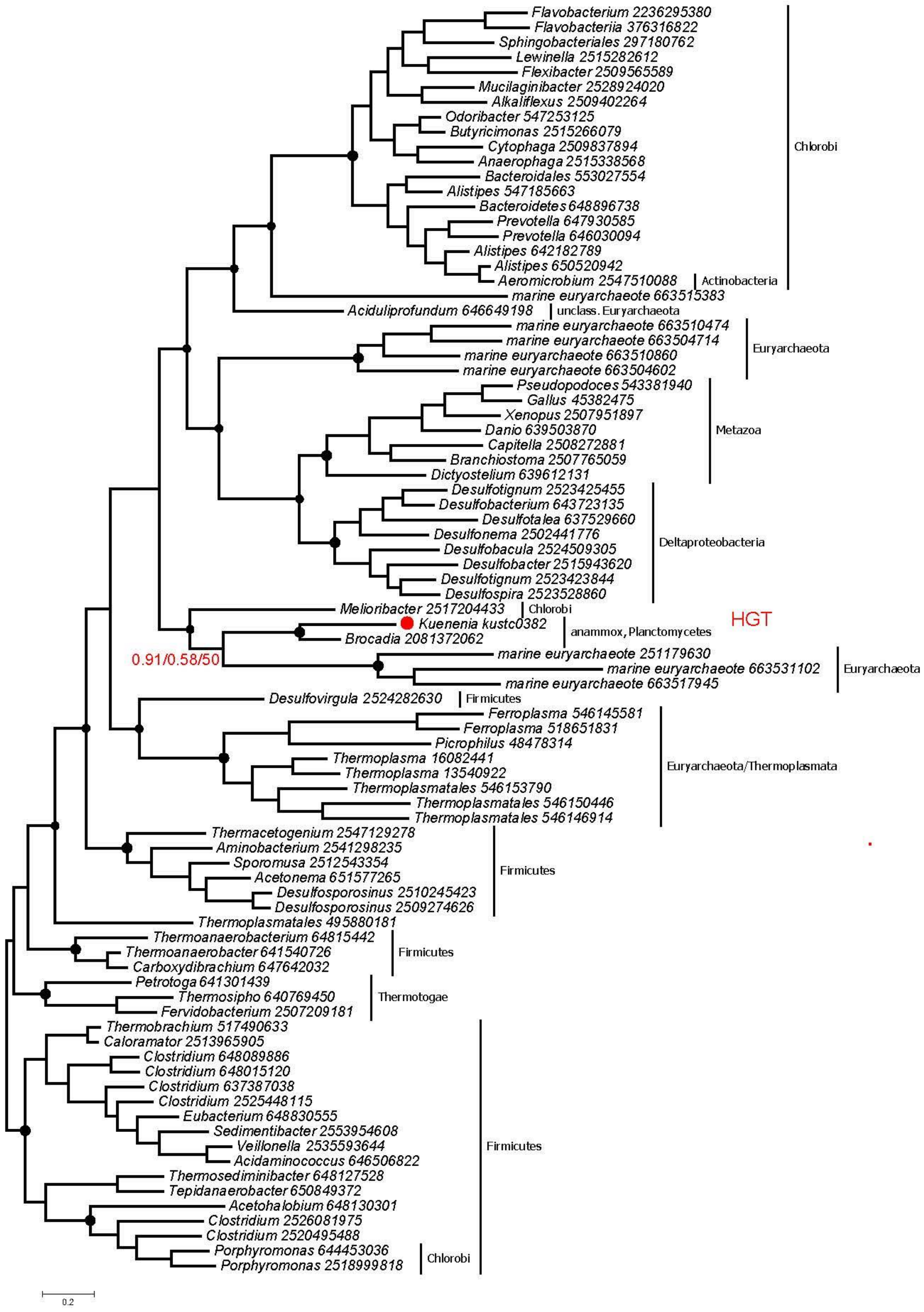

Species: Ca. Kuenenia stuttgartiensis

IMG product name: Glutamate formimidoyltransferase

taxa: 85

sites: 484

PhyloBayes BI maxdiff = 0.0634545

HGT: Anammox <-> marine euryarchaeota

T03 Back to trees-list

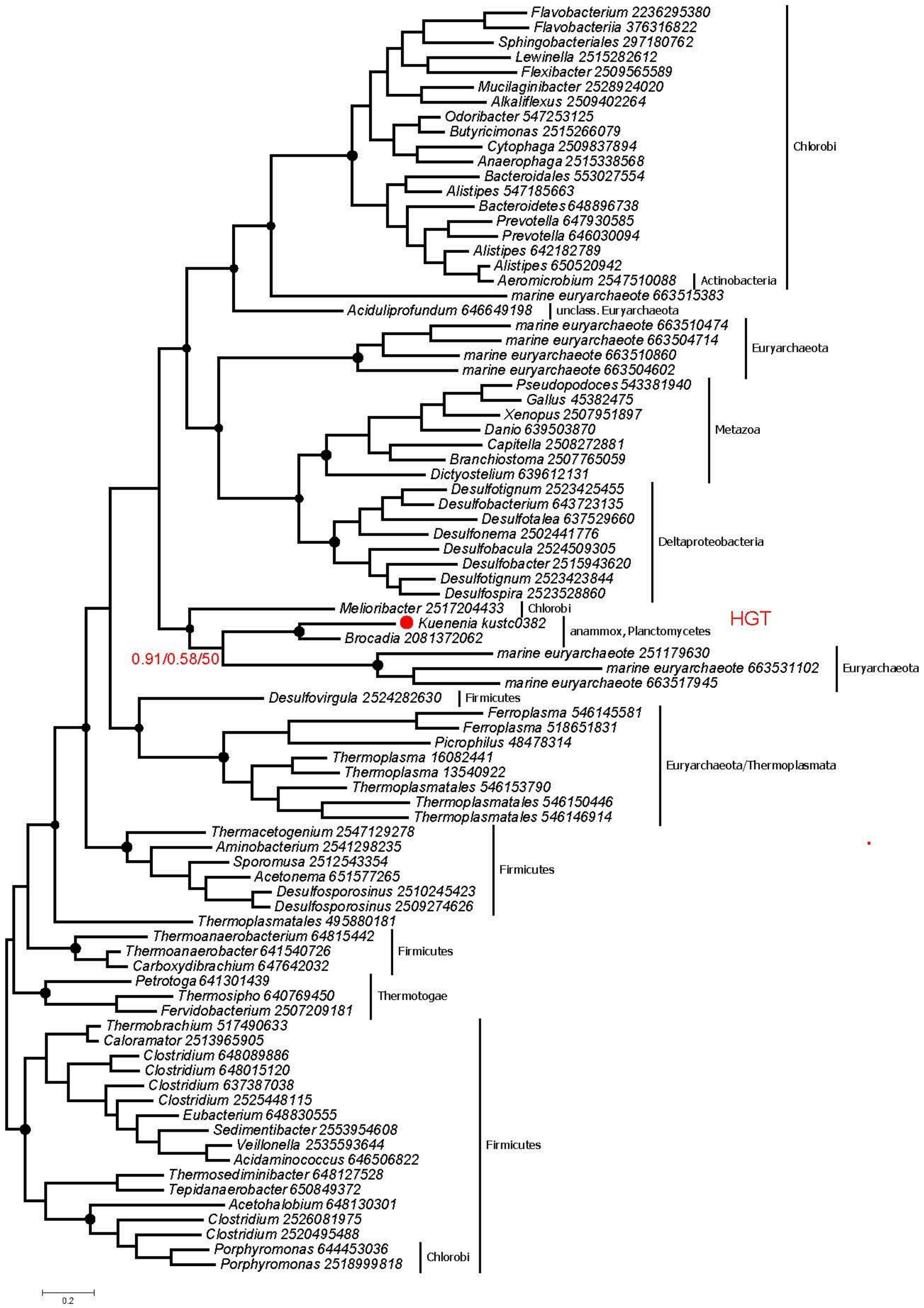

Species: Ca. Kuenenia stuttgartiensis

IMG product name: Glutamate formimidoyltransferase

taxa: 85

sites: 484

PhyloBayes BI maxdiff = 0.0634545

HGT: Anammox <-> marine euryarchaeota

T04 Back to trees-list

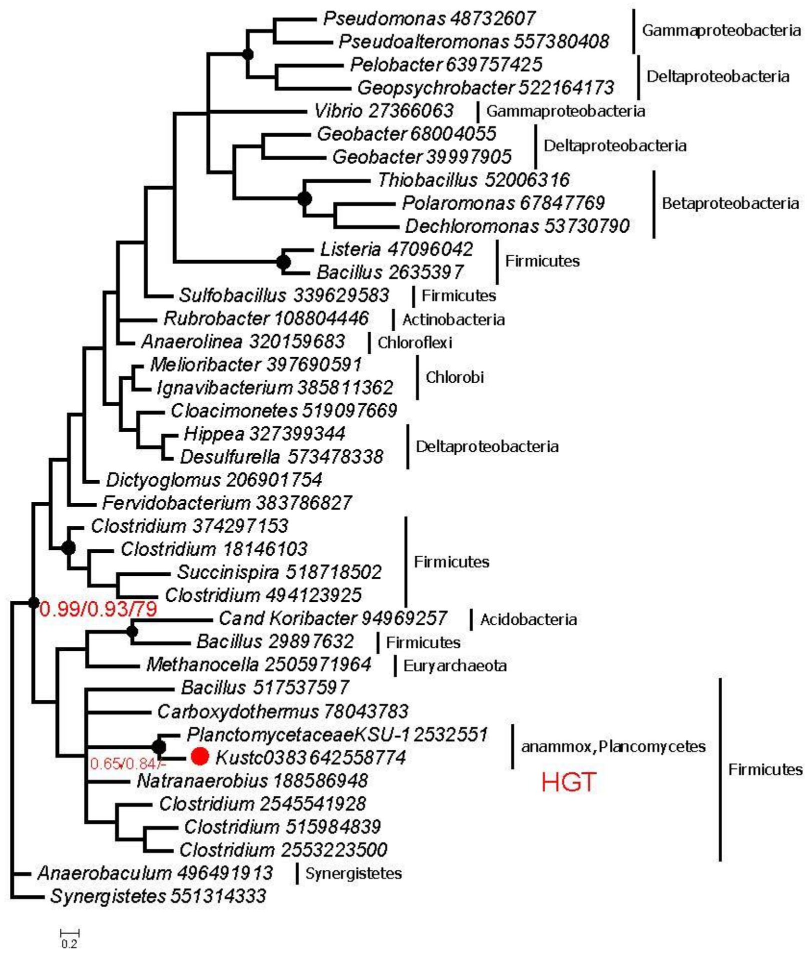

Species: Ca. Kuenenia stuttgartiensis

IMG product name: Glutaredoxin-like protein

taxa: 39

sites: 74

PhyloBayes BI maxdiff = 0.0871134

HGT: Firmicutes <-> Anammox

T05 Back to trees-list

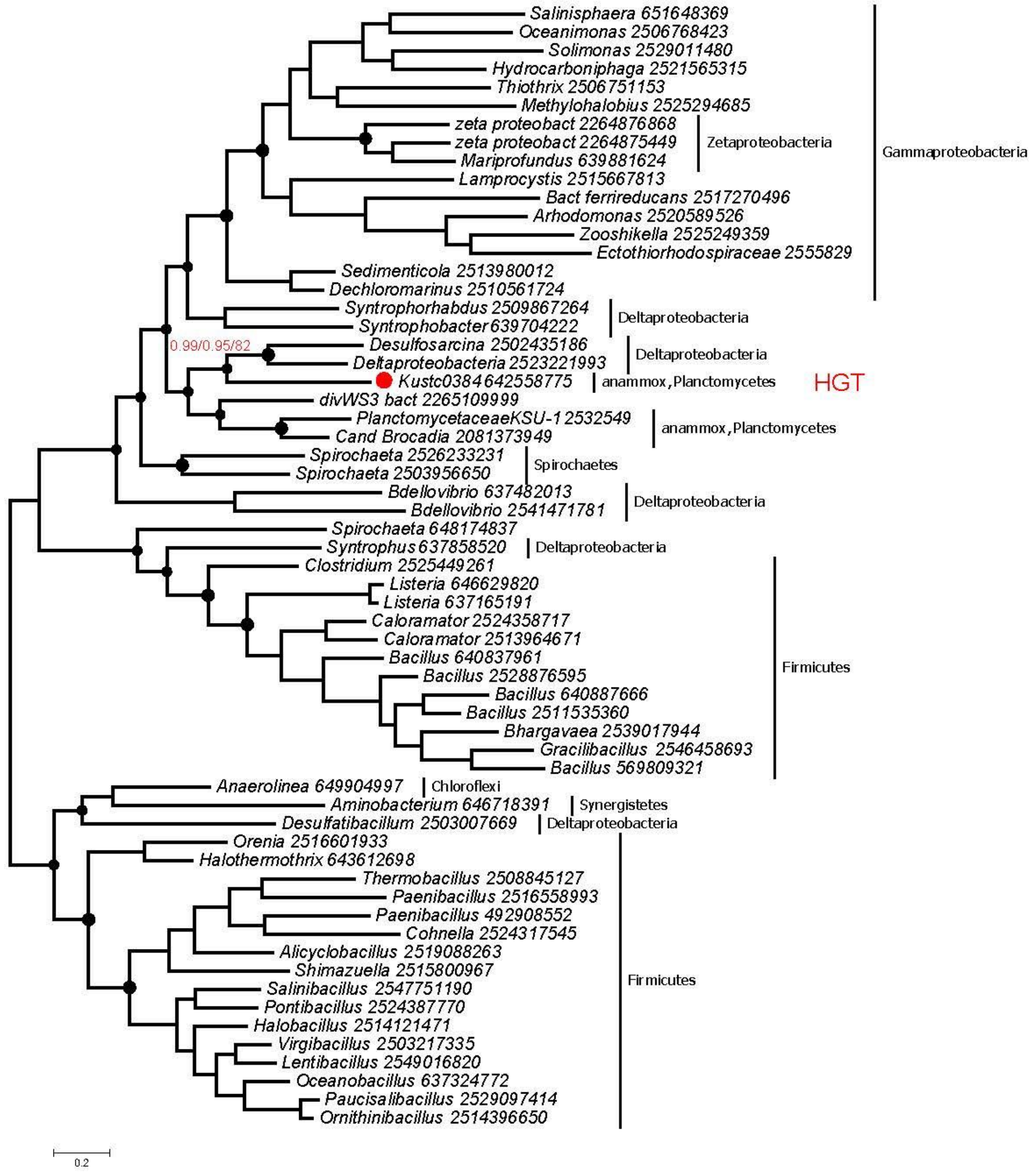

Species: Ca. Kuenenia stuttgartiensis

IMG product name: Permease of the major facilitator superfamily

taxa: 61

sites: 389

PhyloBayes BI maxdiff = 0.0926899

HGT: Anammox <-> Deltaproteobacteria

T06 Back to trees-list

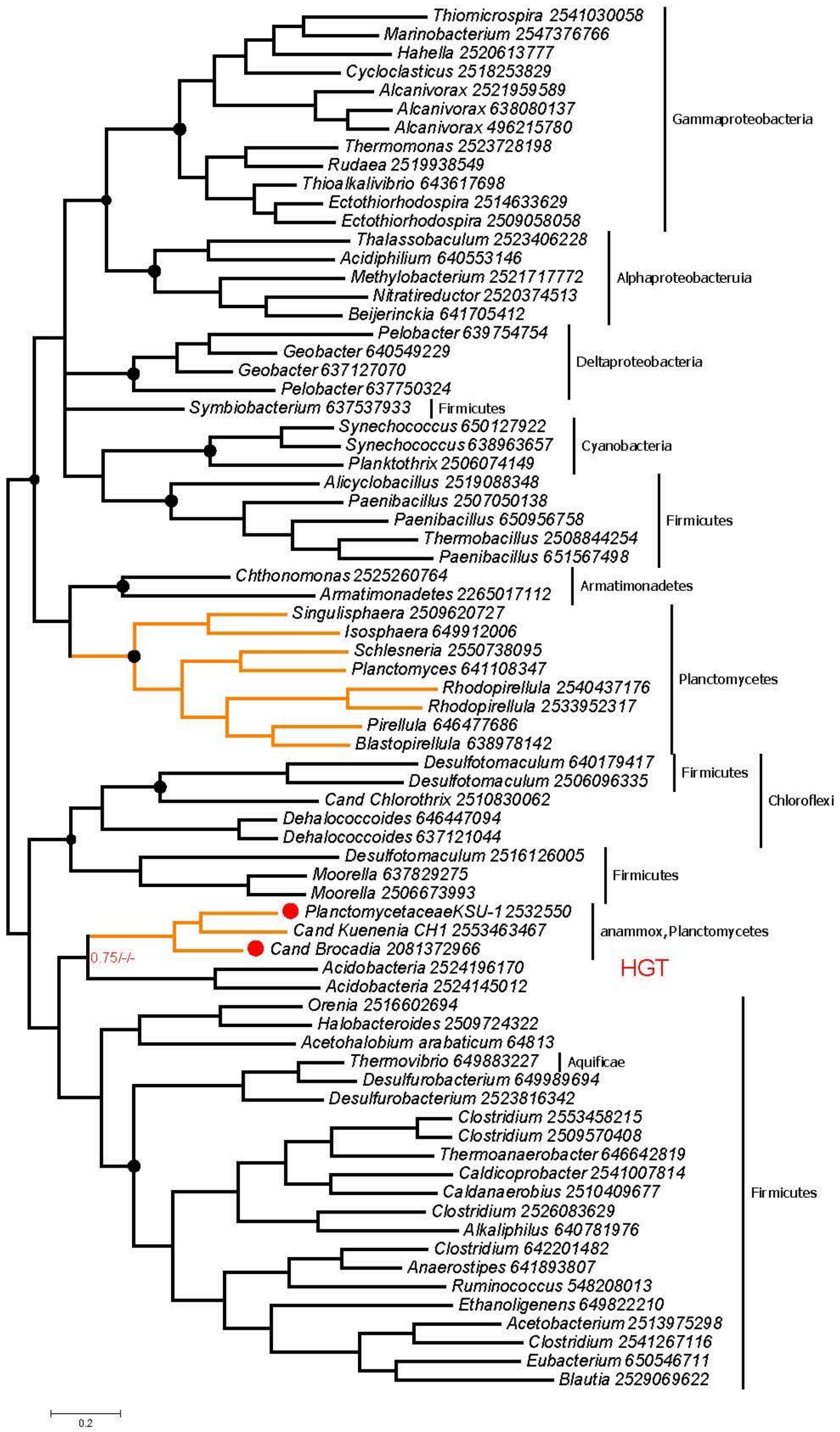

Species: Planctomycetaceae bacterium KSU-1

IMG product name: Indole-3-glycerol phosphate synthase

\# taxa: 74

\# sites: 238

PhyloBayes BI maxdiff = 0.0589449

HGT: Anammox <-> Acidobacteria

T07 [Back to trees-list]

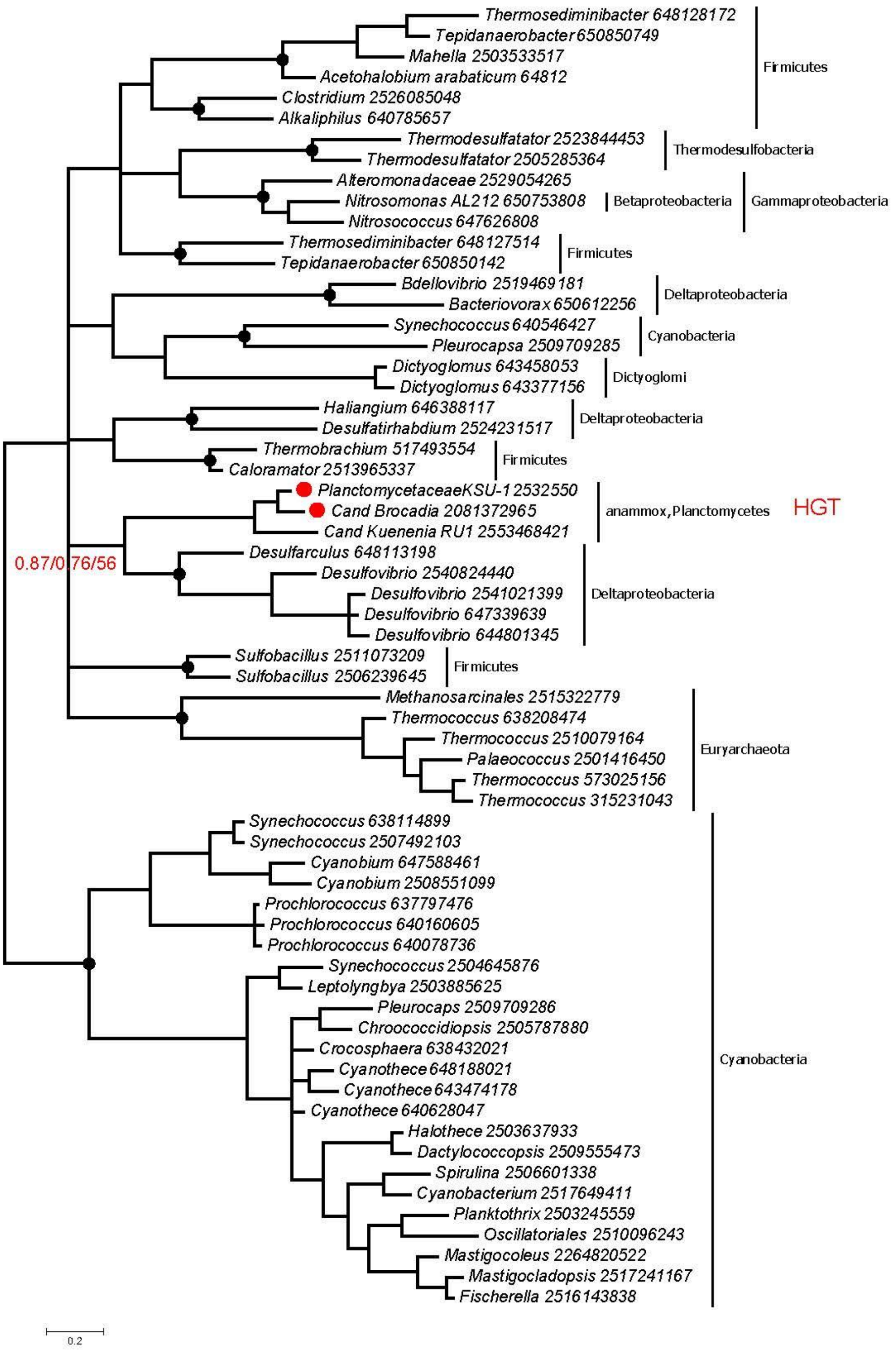

Species: Planctomycetaceae bacterium KSU-1

IMG product name: Conserved hypothetical protein

taxa: 63

sites: 88

PhyloBayes BI maxdiff = 0.0818097

HGT: Anammox <-> Deltaproteobacteria

T08 [Back to trees-list]

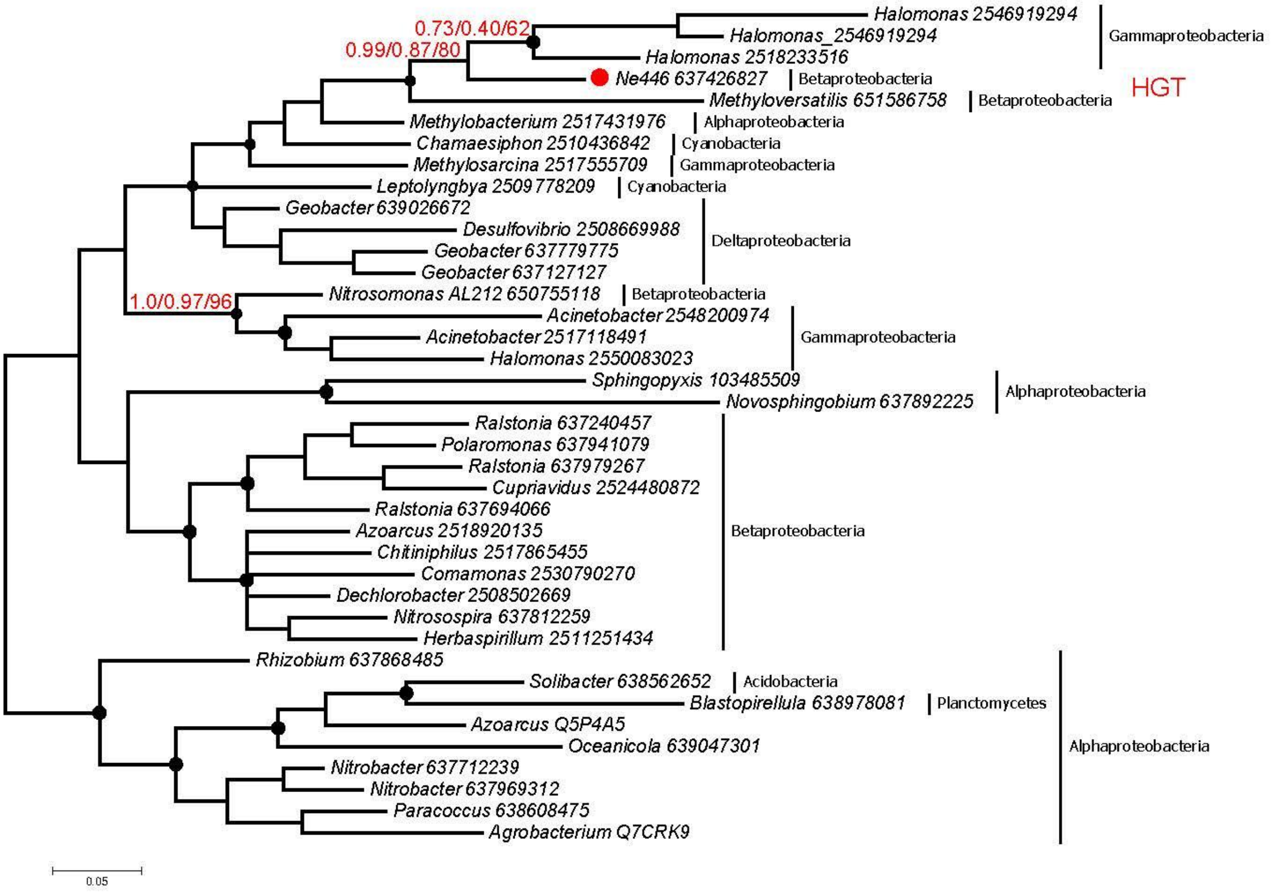

Species: Nitrosomonas europaea ATCC 19718

IMG product name: 3-demethylubiquinone-9-3-methyltransferase

taxa: 39

sites: 156

PhyloBayes BI maxdiff = 0.0556968

HGT: Betaproteobacteria <-> Gammaproteobacteria

T09 Back to trees-list

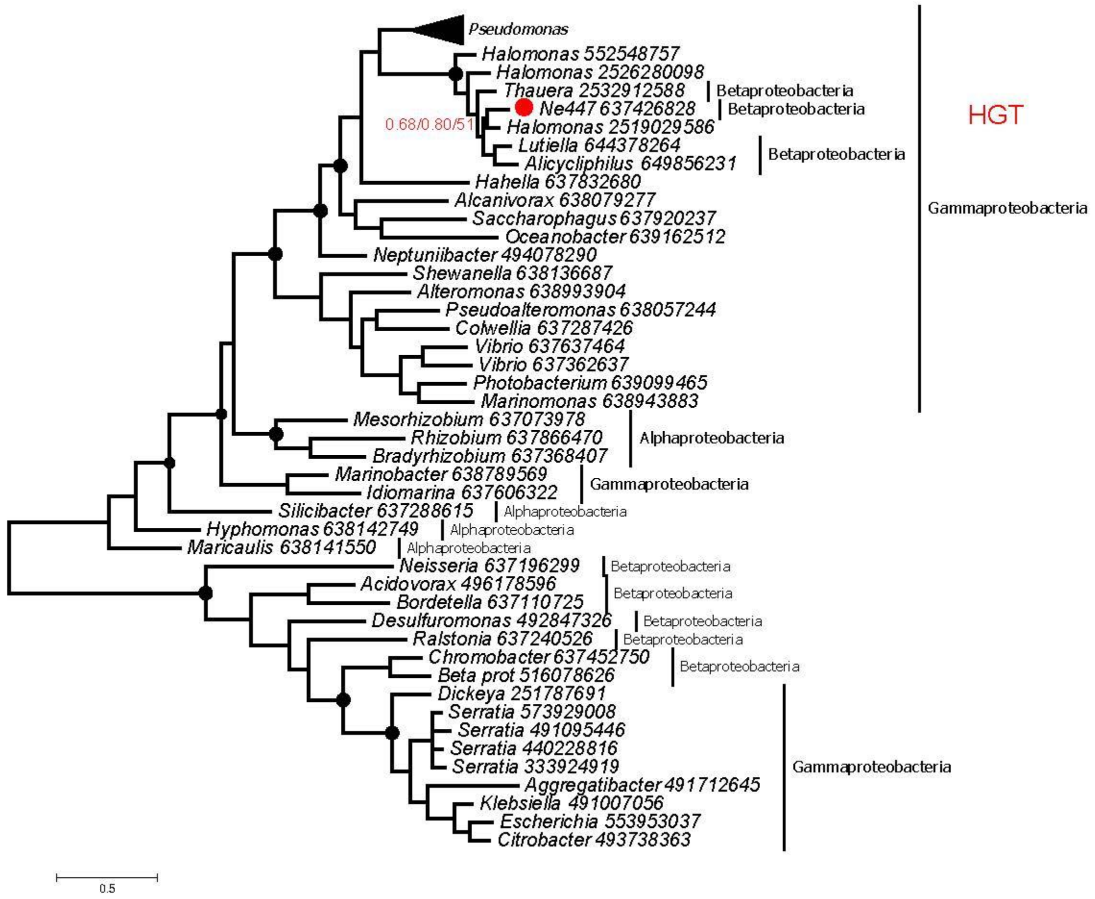

Species: Nitrosomonas europaea ATCC 19718

IMG product name: 3-methyladenine DNA glycosylase I

taxa: 59

sites: 189

PhyloBayes BI maxdiff = 0.0789351

HGT: Gammaproteobacteria <-> Betaproteobacteria

T10 Back to trees-list

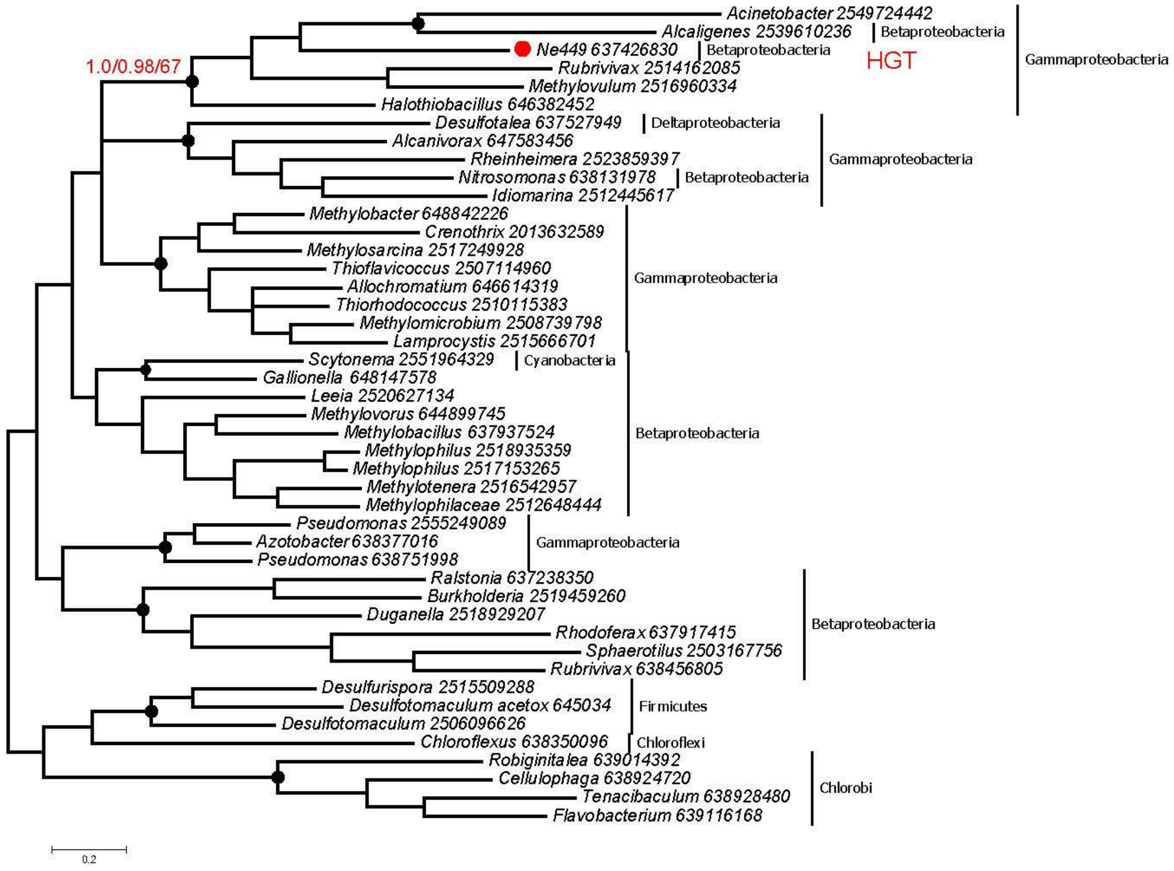

Species: Nitrosomonas europaea ATCC 19718

IMG product name: Aspartate and glutamate racemase

taxa: 45

sites: 238

PhyloBayes BI maxdiff = 0.0977078

HGT: Betaproteobacteria <-> Gammaproteobacteria

T11 [Back to trees-list]

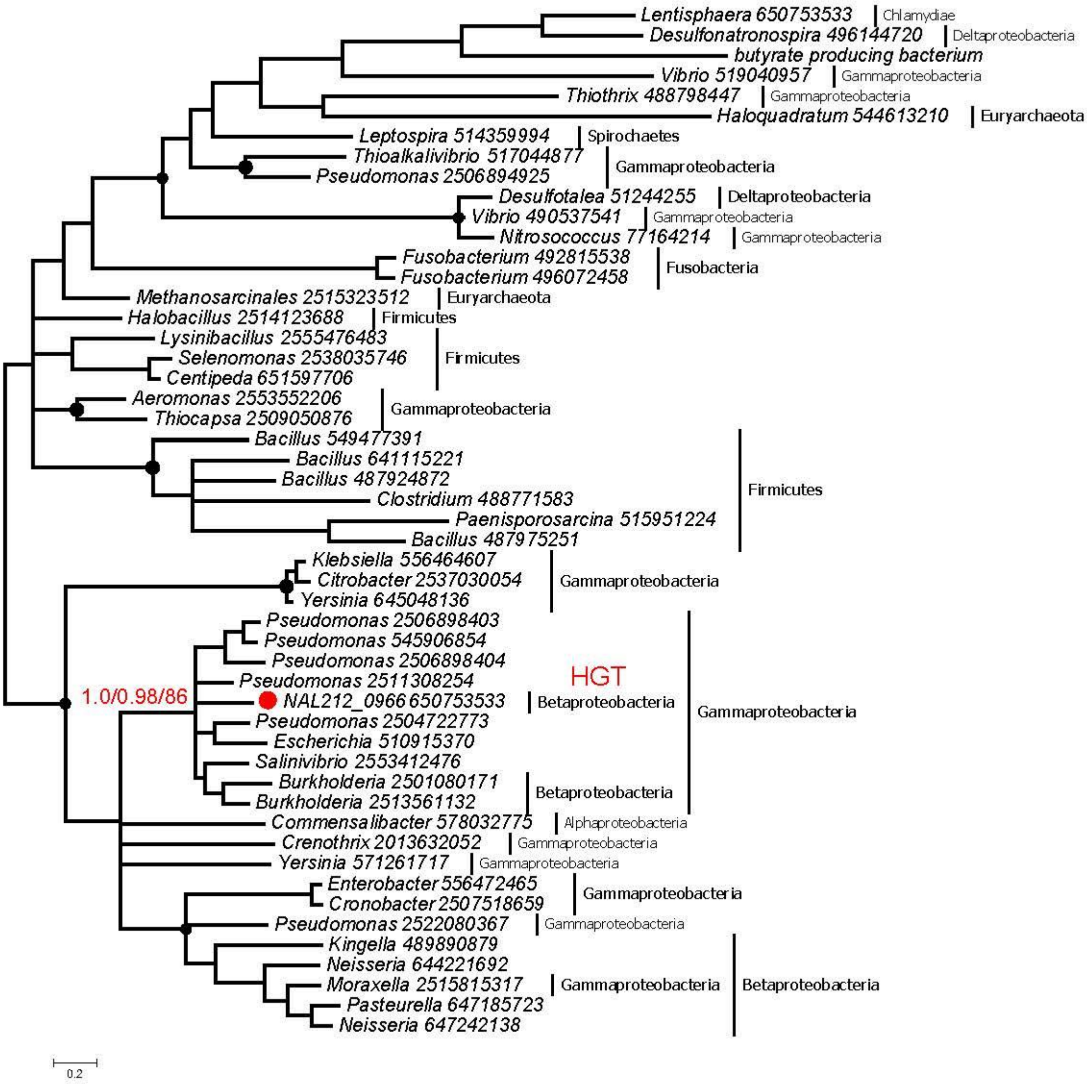

Species: Nitrosomonas sp. AL212

IMG product name: Hypothetical protein

taxa: 51

sites: 141

PhyloBayes BI maxdiff = 0.0651095

HGT: Betaproteobacteria <-> Gammaproteobacteria

T12 Back to trees-list

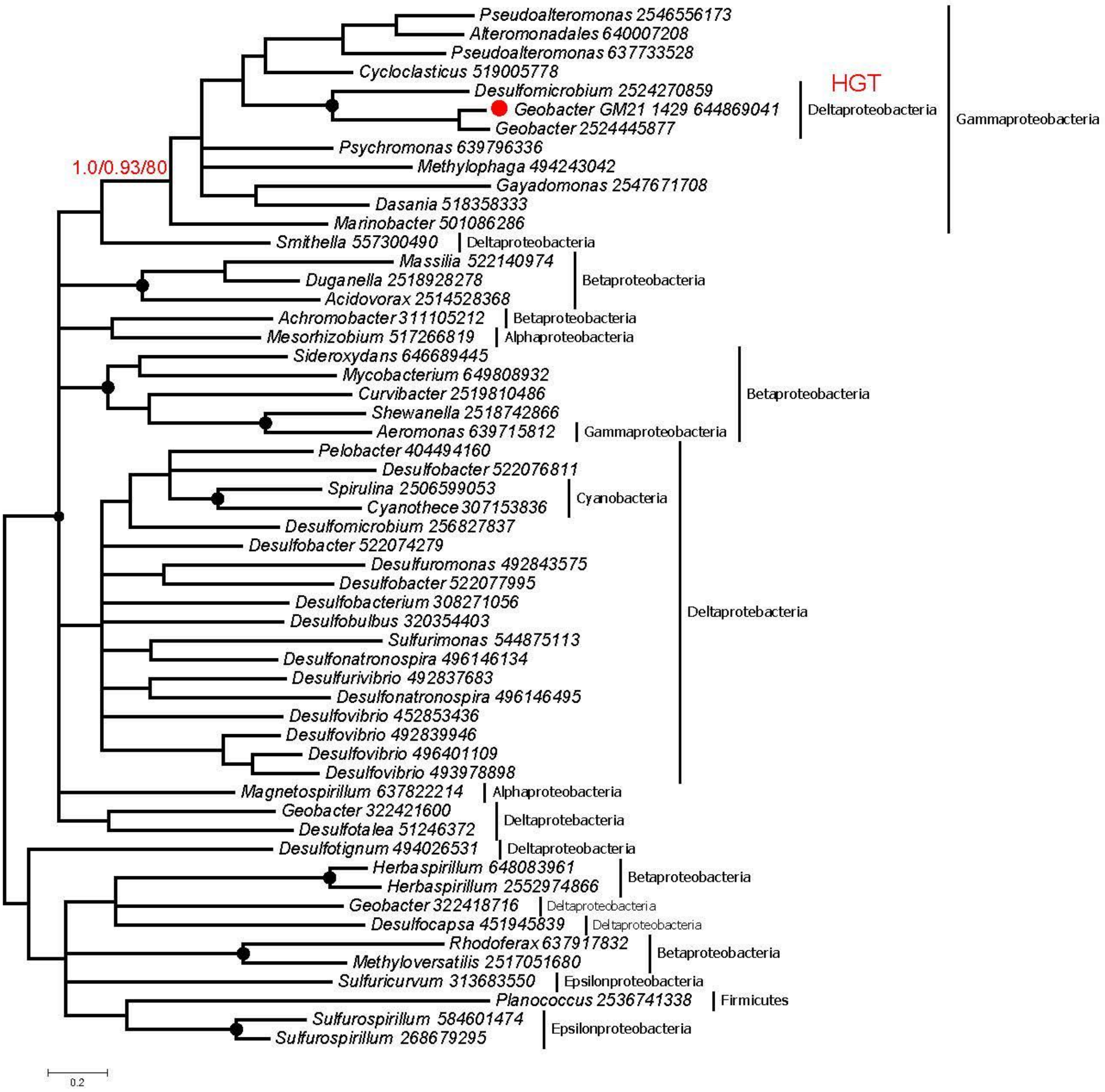

Species: Geobacter sp. M21

IMG product name: Diguanylate cyclase

taxa: 55

sites: 156

PhyloBayes BI maxdiff = 0.0870452

HGT: Gammaproteobacteria <-> Deltaproteobacteria
