# Supplementary material for: Horizontal gene transfer drives the evolution of Rh50 permeases in prokaryotes
Source: BMC Evol Biol. 2017 Jan 3;17:2. doi: 10.1186/s12862-016-0850-6 (PMC5209957; doi:10.1186/s12862-016-0850-6)
Supplement: Additional file 5: — Rh50 protein dataset (90 sequences). (DOCX 53 kb) [file 12862_2016_850_MOESM5_ESM.docx]

>Bacteroides_cellulosolvens Q332DRAFT_00340 2571048935 Ammonia permease [Bacteroides cellulosolvens DSM 2933 : Q332DRAFT_scaffold00001.1]firmicutes

LKKKFWLYVLSCCAAVLMPLQAFASENGSVVNDGLRQVEQYNFSINILAMLLIGFGFLMV

FVKKYGYSATTGTFLVVGAGLPLYLLLRSTGVISKEPISADSINALLLAEFACAAALIAM

GAVLGRLRVYQYAILAIIIVPVYMINEWLVLDGGLDITKGFFDSAGSIIIHAFGAYFGLG

LTIALTAANDRDKAIESDETSDRFSMLGSMVLWVFWPSFCSAIVPHEDMPKTVINTILSL

CGATLITYIASTIFRKGKTSIADIANAALAGGVAIGATCNVVSAPGAFGIGVLAGTLCVV

GYVFIQPKLQAILKGVDTCGVHNLHGMPGLLGGTLAIFIKPGIITAQVAGILITVVLAFV

SGSIAGFIIKATGKKQLVYEDKDEFIVE

>Clostridium_scatologenes Clostridium_scat EG59DRAFT_06439 2580384466 ammonium transporter (TC 1.A.11) [Clostridium scatologenes ATCC 25775 : EG59DRAFT_JIDX01000124_1.124]

MTQLTTKKIVENIPKGSEKKGLLIFLLLIFMCAALILIPSINHNVSVGISAINSQSFKVS

TNATQYGTIFDTLKYERSIHILAMLLVGFGFLMTFVRNHGYTSLTATFIVVSIAIPMYVV

IKSFGGESFVKSTISIDTLIYAEFAAASLLIAIGAPLGRLKMDQYIIMGLLFIPAYIFNE

WLLLDSGMFKGFLDTGGSIVIHAFGAYFGLGVVASTLSKFKNNPVCENTPNSNQFSLLGS

MILWIFWPSFTSAIVAPERVVLTAINTIFALCGATLATYIFTKLIRGKIEIEDIANAALA

GGVAIGSTCDMTTPGYAMLIGIAAGALSVIGYSIIAPKLEKVIRGTDTCGINNLHGMPGI

LGGLVAVFITGNVGVQLLAIFINVVIAFVCGRITGLFISLLGKKQETYNDKDEFFVEKLG

A

>Clostridium_litorale CLIT_00184 2561521460 ammonium transporter (TC 1.A.11) [Clostridium litorale W6, DSM 5388 (CLIT) : CLIT_NODE_31_.2]

MSQALKSKNKTTEHMGQKNAPSKGILISFLVFAVAVAILFAPSLNSNVAQGMEGFNANAA

SSSDTVSQYGTILDTLQYGRSIHILAMLLVGFGFLMAFIRKHGYSSITATFLVVSVAIPM

YMLIKSFGGEHFAMSTIDIKTFLFAEFAAASLLISIGAPLGRLRMDQYILMALLFIPAYI

FNEWLILESGYFHGFIDTGGSVVIHAFGAYFGLGVVANTFSSFKNDPACENDSTSNQFCL

LGSMILWLFWPSFTSAIVSPDRVVLTAINTIFALCGSTLATYIFSTLLRGKVEIEDIANA

ALAGGVAIGSLCDITSPGYAMLIGIGAGALSTIGYSIIAPKLESLIKGTDTCGINNLHGM

PGILGGLTAIFVSGNTTVQLGGIVVTLIVAFVCGRASGFIISMFGKKAVPYSDQDEFMLD

H

>Anaerovorax_odorimutans 2524774977 K292DRAFT_01330 ammonium transporter (TC 1.A.11) [Anaerovorax odorimutans DSM 5092 : K292DRAFT_scaffold00006.6]

MLSSNTNAVVNGTSEVIHQVQQYDFAIAILAMLLLGFGFLMVFIRNYGFSAITGTYMLVA

AALPVYLGLKSMGILSEEVIKADSVEAFIFAELAAAAGLIAMGAILGRIRVYQYAILGAL

IVPFYMLNEWLVLDGGLGITEGFVDAGGSIVIHAFGAYFGMGLILVLNTKNKNVKIESDA

TSDRFSMIGSMILWIFWPSFCSALVPEAQFGQTVVNVILALCGATLCTYITGSILRKKIA

IADIANATLAGGVAIGATCNLVSPIGAFIVGLIAGALCVIGYVCIQPRLQRIFKTIDTCG

VHNLHGMPGLFGGLFAIAVVPSAAKAQIIGIIFTIVLALVAGAISGVILKITGTKELAYE

DSEEFLEEE

>Clostridium_carboxidivorans gi|255525972|ref|ZP_05392897.1| Rh family protein/ammonium transporter [Clostridium carboxidivorans P7]

MLLIFIGAALILIPSINHNVSMGISAINDQSSKVNTNAAQYSTIFDTLKYERSIHILAML

LVGFGFLMTFVRNHGYTSLTATFLVVSIAIPMYVIIKSFGGESFAKSTISIDTLIYAEFA

AASLLIAIGAPLGRLKMDQYIIMGLLFVPAYIFNEWLLLDSGMFKGFLDTGGSIVIHAFG

AYFGLGVVVSTLSKFKKNPVCENTPNSNQFSLLGSMILWIFWPSFTSAIVAPERVVLTAI

NTIFALCGATLATYIFTKLIRGKIEIEDIANAALAGGVAIGSTCDMTTPGYAMLIGIAAG

ALSVIGYSIIAPKLENVIKGTDTCGINNLHGMPGILGGLVAVFITGNVGIQLLAIVINVV

IAFVCGRITGSFISLLGKKQETYNDKDEFFVEKLGA

>Geobacter_M21 gi|253700053|ref|YP_003021242.1| Rh family protein/ammonium transporter [Geobacter sp. M21]

MKKCICYLLVMVTVLLLAGVAFASGSVGTNLQELSDVQKYNRAIHIMAMLLVGFGFLMVF

VKKYGRSAITATYLLTSVAIPLYFLKDSLFPPLAEESVIDKLILAEFAAASLLICAGAVL

GRLKMNQYLLLGILFVPFYSLNEWLVLNGGLGLITGKVVDTGGSIVIHAFGAIFGLAVAA

TMTTQEEYATPIECDDTSDRYSLLGSMVLWVFWPSFCAALVAPADVPRTAVNVILALCGS

TLATYFATVRLRGKISAADIANATLAGGVAIGSTCDLATPAAAFIIGIMAGVISTFGFAV

IQGRLTDLLKKVDTCGVLYLHGMPGIFGGLAALFIVTGINSGAQLTAIALTAVLAATAGL

VSGKVIALFGHRAEPYTDAEEFEGEEVEEELFPVSAVPELDTE

>Clostridium_papyrosolvens_DSM CpapDSM_2914 645656863 ZP_05496744 Rh family protein/ammonium transporter [Clostridium papyrosolvens DSM 2782: NZ_ACXX01000029]

MKKGLFVLLSGFILTLFMPFSVFASTNEAVDGIRQVQQYNFSINILAMLLVGFGFLMVFV

KKYGYSATTGTYLVVGAGIPLYLLLRYTGVISLESVNPQTIKGLLLAEFAVAAALISMGA

VLGRLRLYQYALISVFLVPIYMINEWLVLDGGLGITKGFVDAAGSIVIHAFGAYFGLGLA

IALTKQKHMDHPIESDASSDRFSMLGSMVLWIFWPSFCSAIVPAEQFEQTVVNTVLALCG

ATLITYLFSALLRKGKPAIADIANASLAGGVAIGATCNLVSAHVAFLIGLLAGALCVVGY

TIIQPRLQRLLKIVDTCGVHNLHGMPGLLGGIIAIFVVPEAAKAQVAGIVFTVVLAFVGG

IISGNIIRLTGSKEAVYEDGDEFEEAVEEAA

>Dehalobacter_11DCA Dehalo_DCA 2520955849 Ammonium transporter [Dehalobacter sp. 11DCA : CP003869]

MKKKIGVFILGCLISLFIPISLFAAEPGSLSEGIRQIEQYNFSIHIMAMLLVGFGFLMVF

VKNYGYSATTGTFLVVSVGLPVYMLIRSTGVLSLEPISANNIHALLLAEFACASALIAMG

AVLGRLRVYQYAILALLLVPAYMLNEWMVLDAGLGITKGFVDSAGSIIIHAFGAYFGIGM

TLMVTQKADREKAIESDETSDRFSVLGSMVLWIFWPSFCSAIVSPEQMPQTVLNTILALC

GATVITYILSSILRKGKTSIADMANAALAGGVAIGATCNLVTASVAFLIGLLAGALSVVG

FVLIQPRLQSSLKIVDTCGVHNLHGMPGLLGGIIAIFVVPGIARAQITGILFTVVLALLT

GVVAGLIIRITGKKQLPYEDKDEFAA

>Monosiga_brevicollis Monosiga1_choa c1 gi|167527454|ref|XP_001748059.1| hypothetical protein [Monosiga brevicollis MX1]

MASRRFGVLALLSQIILLILFVVWVRYDGDLGDSSGMDQSCATRLTEIDCLAAGCTYNAA

ESTCSGTPTILDQRYPYYQDVHVMIFIGFGMLMTFLSRYGFSAVAYNMFLACIAFQWAQF

TNTFWHRIFEGEAASHKIRMTVENLITGDFAAASVLVAYGAVIGKTTPEQLLVMALLQLI

FYSLNESIGVIEFQAVDVGGSMYVHAFGAYFGLAVSFMLGNSRPTASGKQLHRNNSNNGS

SSTSDVFAMIGTLFLFMFWPSFNGALASGSAQHRVVINTVIAIAFSAMSTFAFSNLFRRG

RFNMVDIQNATLAGGVAVGSSADLLIEPYGAALIGFVAGLISVVGYVYISGLLDRYLRIH

DTAGVHNLHGMPGVVGGLGGVIAASLAKASDYGSALGEIYAARATGTTAGEQAGNQLLAL

VVTLGLAIGGGLATGFVLRLLPAPQSPFHDREFWDIEEHAGAEASDEPDTIPLGSVTKVS

PLM

>Acanthoeca Choanoflagellida CAMPEP_0182971650 /NCGR_PEP_ID=MMETSP0106_2-20121125|9206_1 /TAXON_ID=81532 ORGANISM="Acanthoeca-like sp., Strain 10tr" /NCGR_SAMPLE_ID=MMETSP0106_2 /ASSEMBLY_ACC=CAM_ASM_000206 /LENGTH=504 /DNA_ID= /DNA_START= /DNA_END= /DNA_ORIENTATION=

PTGSLAGGAKMSDDEHTPLVAPKSPTNGSAATAVTNLLSYQVVIIALLGVFTTYVPNGHV

PENYGMMLNISVMIFVGFGYLMTFLRKYGYGAVGFTFAISAVVIEWAIINIGLWGCVEGD

DNSFGVVCHIPNHLGVRRFGVGIDMIVQGLFAAAAVMISFGALIGKASFDQMIVLALVET

SVYSVNCWVYFNKIKAADTGGSVVIHTFGAYFGLAATAFLTPKSKRRDFSEMTSSYTSDI

FAFIGTVFLWIFWPSFNAVTADPSLQERAIVNTFLALLSSCIVSFFCSRRFRGEGLFDAV

DIQNATLAGGVAMGTACTLPLEPYGACILGTLAGVLSCAGYAFMLERLEKKGVHDTCGVH

NLHGMPGILGGLAGVLMMGVASTATAREQGFLPHRHRQWWLQLCGLAVTIFVAIVSGALT

GSIVNAAIPVSYKMFYTDESHWTVPTGELSFTKNDVKEDAPRRESMRQRRSQAVRNWTKV

GTAVRAASLLRRAASAGQNTSVNN

>Salpingoeca_rosetta Pterospo_ros_choa 2508464315 Ammonia permease [Proterospongia sp. ATCC 50818 : supercont1.16] Pterospongia rosetta Salpingoeca rosetta] Choanoflagellida

MSLFSDRQPFANNAAASLSSFSSGQTPSAYSHHDGENDHDMSRVEPMMMMEGEPVSSSKG

RGQRGGFTDRFSLGLFLAEAAIIVLYGVFVEYGDTADPRQPDSTAVTDYYGMFRDVHVMV

FIGFGFLMSFLNQYTLSSVGLTFLVGAFALQVSMLVVPFWHRVFAGGWVKIPLTLETLVT

GDFGAATVLISMGAVLGRTSPLQLLVMVVIELFAYGFNEALLVNEIKVADVGGSMIIHAF

GAYFGVACAWVLGPRNADQTNNASSRYSDTFAMIGTVFLWMYWPSFNGVLAGDTGNSRHR

TILNTLLSLSASCIVTFVVSRMLRGGKFSMVDIQNATLAGGVAIGASANLFTTPWGAMTV

GSIAGIISTVGFARLQDLLQRKFGLYDTCGVHNLHGMPGILGGLAGALFAGISTVDDYSA

TTLTHVIPSRADRTAGQQAGYQLLGLAVSIGMSLVSGALTGVVLRMPGLQQPSSLYDDEE

DWAKEEE

>Picocystis_salinarum Viridiplantae; Chlorophyta CAMPEP_0183834054 /NCGR_PEP_ID=MMETSP0807_2-20130328|6393_1 /TAXON_ID=88271 /ORGANISM="Picocystis salinarum, Strain CCMP1897" /LENGTH=589 /DNA_ID=CAMNT_0026080039 /DNA_START=1 /DNA_END=1768 /DNA_ORIENTATION=-

XFVGYEKGPHTSTTVVEYYMYYIHVVRERNERNVEMMVACEHEEDETSVERRKLYSVPGA

DASTTDSCAWGVRCTQATMVFVGFGFLMTFLRRYSYSAVGLNFYLSCMCMLEFFFPSRQP

IRRFRRCGTDGMAEPTSEPLLNTTADKWNLRDSFATSSAVVMAALLVLLSVFVGYEKGPH

TSTTVVEYYMYYIHVATMVFVGFGFLMTFLRRYSYSAVGLNFYLSCMCMLEFIFFGGAAQ

QGLFAGKISRIVLDLPLMIDSAFCAAAAMISFGAVIGKVAPAQILWLLAIEVPIYVFNVW

LAVDVLGCLDMGGSVTIHAFGAFYGLGAALVLSRPGAGSSHPKNGASYISDVTAMIGTVF

LWIFWPSFNGAMASSAGPGLEDQQFYCVVNTVVSLTGACLMAFTVSSFVENKLNMVHIQN

ATLAGGVAIGSSANFAMVPGVSLLIGLCAGALSTLGYCFIMPSLENAIKLQDTCGVHNLH

GMPGLFGGLVAALVSIFGAGANTALLPLGGATWWHQIIAVITTFVISLCAGALAGWIVGK

VQIPSTKQKLAVEDLYDDSLFWSEVEAEEESSHVFDLSPEVMHALFVMP

>Methanomassiliicoccus_luminyensis Methanomass_lum1 2518908567 Ammonium Transporter Family protein [Methanomassiliicoccus luminyensis B10 : CAJE01000004]

MRLKKRTEMMIALVVACLAIMIVATWNVSAAGEELTTPSGEPGSTSDSSLIQDENTKYSK

NMDIWFMLMLVAFLMIFIRKYEWGVALATLLVTAGSFLAYMATQQFYFDQAWDQTLMIRG

VICSITVVIAIGVFLGTIKMWQYLMVGALFGPIYAFVEWFLFQYLSGVVDPGGSILVHMC

AAYFGLGVALAIRDKRAFNEPQYTTTHSVSFVWLASMLLFILWPSFVTSLLPADQVTWGM

ITCYMAGIGSIITTYIVCQAVQKKVNPLIFTYAMLAGPVAIGSPLLSVNQWGALVIGLVA

GVVSALSFIYLQPWLTKKIGAMDVMGVHNLHGMGGWTGALAAALIVGSATNAIAAVSVAL

ITLVTGAIVGAVVRVTRGKISDELLFNDDLDFIKNEMPSSASALNEMGAEADREVSGATH

>Methanomassiliicoccus_intestinalis Methanomass_int_eury 2555937688 H729_04210 ammonium transporter [Candidatus Methanomassiliicoccus intestinalis Issoire-Mx1 : CP005934] Euryarchaeota; Methanomicrobia

MDAGRKKLAAAILVVACLALLCITLVPTNSSQEDIAANQDIALSSGEEMLTPSDEPGSSD

QSFTDDEMSRYSKNMDIWFMLMLVAFLMIFIRKFEWGVALATLLVTAGSFLSYMAIQQFY

FGADIWDQSLMIRGVICSITVVIGIGVFLGTIKMWQYLLVGVLFAPIYSLVEWFLTAAPY

LNDLGIGTVTDPGGSIMVHMCAAYFGLGVLLALREKRAFKEPMYTTTHSVTFVWLASMLL

FILWPSFVTSLLPADQVTWGLVTCYMSGIGSIITTYIILEITQKKVNPLIYTYAMLAGPV

AIGSPLLLVDQWGALVIGLVAGAVSALAFVYLQPWLCKKMGAVDVMGVHNLHGVGGWIGA

LSVVVITGELVNAVAAVCVAALVLVLGAVVGLIVRFTRGKMTDEMLFSDDADFIKTEDPS

QTYIQTKLDEDPTNTDAPGPGTA

>Methanosalsum_zhilinae Methanosalsum_Eury 2502871530 Mzhil_1521 ammonium transporter [Methanosalsum zhilinae WeN5, DSM 404017 : Sent_unknown] Euryarchaeota; Methanomicrobia

MGNSMFRGTLLLILVMCLVNLLVPAVSADLTTPTGQEGLTEDMTHLEDELHKYHKNMDVW

FMLMLVAFLMLFIKKFEWGICLATLLVLAGSFITYMAIQQFVFGEPWSQDLMILSIFCSI

TVVIAIGVFLGTVKMWQYFLAGILFAPAWIAIDWFMFGYLEGVVDPGGSMLVHMVAAYWG

WGVILALREKRAFNEPMDTSTHSISFVWLASMLLFVLWPSFVTALLPADLVNWGMFTAYM

AGLGSIIAAYFTCMLLQKRVDPLVYTYALLAGLVAIGSPLISVDPWTALGIGLVAGTVSV

FCFVKLHPWLCEKAGVLDVMGVHNLHGVPGIVGAIFGAIFAAGMVNIISLVGVLILSLVT

GAITGLILKATRGDMDESLMFSDNADFLGWKPEPVVTEDGKVVAPASRSGEKILNE

>Trichomonas_vaginalis_428240 2508158139 rna_TVAG_428240-1 Ammonia permease [Trichomonas vaginalis G3 : DS114300] Eukaryota; Parabasalia

MLIVLLLSLASCNGPSEIPNDIIPEQNYTDDTIYPKVVDVWFMTILVAFFMMYIKKFEWG

VMIAVLLSAATSHVTYAFVKNVCMKHDFDAKLATESVCCAITCTVTIGIFIGTIKTWQYC

IVGVMFGLSYLLVEYLVVSGKAIKGVIDPGCAISIHMMAAYYGLGVACVIREKRVIGVEF

KFSTHSVNWIWLGSSLLFILWPSFVSLFWKGKAAWTAAINCLMSGLGSIISAYFMEFTIK

RGKVDAFVYAVALLAGCVGTSSALFMLSPWSSLLVGAICGCFSVCSFNYIHNPFTKMLGI

NDVMGAHNLHGICSWLSVITCAITLFIKKFPPQWTVAGAVTSFAVSSICGVITGLVLRFT

KGKEIPDSDFMEDNAIFLFPHDTELDWPPKE

>Trichomonas_vag_24790 2508169651 rna_TVAG_024790-1 Ammonia permease [Trichomonas vaginalis G3 : DS113757] Eukaryota; Parabasalia

MFLFLLSLVSCNGPALDTRVVAAANFDTESIYPKVVDVWFMTILIAFLMMFIKKFEWGVM

LATLLSSATVFIFYAFLKAVAVGHDGHREFTERIAAEAVLAAIAYAISIGVFVGTIKYWQ

YIMVGIVFSCGFYLVDWLIISEKVIKGCVDPGGAIAVHMFACYFGIGVALAVQEKRVVGV

EYRFTTHSINWLWLAVTLLFVLWPSFTSIFWKGKEAWEDVITTYMAGLGSIISAYFAELA

CKKGGKIDPLIYAVALLGGCVGISTSLFIVGPWGGLLVGVISGIVSVCSFNYLHPVLQKK

LGIGDVMGVHNLHGVCSWVATFVGLIGAYCKKFPGIWTLVGALISFSVSLIFGLIGGALC

RLLKFGEIPNERFMLDRGDFIFPESEGSDNEEPKKREEQVNEL

>Trichomonas_vag_19830 gi|123497982|ref|XP_001327304.1| hypothetical protein [Trichomonas vaginalis G3] rna_TVAG_019830-1

MFSLINFYFKKFSLSLTINAILTTFSSFVFRYLLFSVAFKEGQNAITFADATLCALSCII

SLGCFNGVVKLFHYILYGILFAATYTLVHWLVIEGDVIKNVIDTGHAIEVHLFAASFGLG

TAIVVREKRIVGTTFENAVDSHHWVLFATLIIAFLWQKYTTIYLTITGVASSKASTAVVM

AVCGSSIVSLVFEHFVQKKIDIYRFANSIFVGCIGIGCSVLIVGPWGALLVGAICGLANT

FLMTPFSKFIEYKIGAADLLSVSGVNGISAWIAIFVGLISAYIKKQKGV

>Acetohalobium_arabaticum 648129640 Acear_1282 ammonium transporter [Acetohalobium arabaticum DSM 5501 chromosome: NC_014378]

MKSKIKNKLVSKSIIFCLLFLFVASTTTYAVEESTAELFQYHRGQDVFFMFMLVAFLMLF

IKKFEWGVCLVTLLTLSVAFPLYVIIQRNLFDFSLGIELIITGIFCAITLIIAAGVIFGH

VKIWHFIPLGILFVPGYILNEWFLFSYLEGVADSGGSILVHMFAAYWGWGVILALQRRDV

NQAEMNATTHSISFVWLASMLLWVLWPSFTTSLLPTDLIITGMTTTYLALMASTLTAFLV

LKWIKGELDPLIYTYAILAGGVAIGSTVDLVGPGTAWLIGAAGGIISVLCFLYLDDWLAN

KTNLTDTMGVNNLHGIPGIFGGLMGIPFAGSVQIYAIAGGIIIPLVTGLIAGIIVKIFEK

PQLLLDDAEIFDINIDLERTQQM

>Citricoccus_CH26A 2548606314 CITRIDRAFT_02198 ammonium transporter (TC 1.A.11) [Citricoccus sp. CH26A : CITRIDRAFT_AFXQ01000017_1.17]

MRSSAISSSHELIAAAAEVTEAGGQTAQLFQFHRGQDVFFMLMLVAFLMMFIRRFEWGVA

LTTLLVLAVSWPLYLIGYTQVLGNDLDIDAVILGVFASITLVIAIGVFLGHLATIHFILA

SVLFVPAYMFNEWFLFGLLDGVLDSGGSILVHMFAAYWGWGVILGLQNRRVSDVPQDTSV

HSVSFVWLASMLLFVLWPSFVTALLPPEAVIPGMINTYLALTASVLVTYVLLWALKRTID

PLVYTYAILAGGVAIGASVDLASPLQSWLIGLLGGAASTLCFVYLHDWLCRKTGVLDTMG

VHNLHGVPGILGGLVPLVLFAAPLDQLWAVLGTLVIGLVTGLVAGLILRLFPPPERMLDD

AEAFPLDEVRAAEAQ

>Kuenenia_stuttgartiensis _381 642558772 CAJ71126 similar to ammonium transport protein amtB [Kuenenia stuttgartiensis genome fragment KUST_C (3 of 5).: CT573073]

MKKLTLVLYIFFIGLISFLFIEGTKVHASALFEDEFDGKSLENSWIILRENQSDISLTES

PGHLRIISKAEDLWQINVNNKIKLLRRGPHGDFEIVARLTYDPKEKFQQAGIILYEDEEN

YVMLTRQKDDAQHVVMSRSVSKTEGAKSAATSLTTLYLKLTKSGESISGAFSTNGETWTT

VDHISGLHFKHPQVGLVGFNAQQKTTANADFDFFKISAIGGGVASAVEELLEVNKYNKSI

HVMAMLMVGFGFLMVYVKRYGWGAATATYIAVSFVIPYYMYLKSKGLFGEVAEFQIDRLI

LAEFCAASMLIAMGAYLGRLKMSQYIIAALLFVPSYMLNEWIMLDDGMGLIPKGKLIDTG

GSIVIHQFGAYFGLGVIIRMTTKEDFSKGIESDKISNQYSMLGSMVLWIFWPSFCAAAAE

ASQMVTAAINTILSLCAATISTYLATTLIRKKIVIEDMANAALAGGVAIGSSCAHTTPRA

ALMLGFIAGILSVIGFALIQPRLQKMIKGIDTCGVHNLHGMPGILGGLAAIFMAGSAVPG

LQIKGVVITFVVAIITGLASGTVVSLFGHRRDSYNDEEEFVVEAH

>Brocadia_anammoxidans 2081372964 WQC04.C15_CD000284_379145_380554 sll0537 COG0004 [P] Ammonia permease [Candidatus Brocadia anammoxidans (WQC04) : Canam_WQC04]

MAMKRFLKLYVVLCIGLLCIPLLWVPKVSGGTAALESEGKNQSIDHALPAMYDIQNDWHA

WSSIDHGSNVVSESHKILLASNSVESHTPAKIESVPSKVEHTGEITSAMEDFLELTKYGK

AIHVMAMLMVGFGFLMVFVKRYGYGAVTATYIAVSIVIPYYMFLKKMDIFGEPAELTMDR

LILAEFCAASILIATGAFLGRLKMSQYIIMALVFVPSYMLNEWIMLENGMGLIPKGQLID

TGGSIVIHQFGAYFGLGVIVRMTTSEDFNKKIESDKISNQFSMLGSMVLWIFWPSFCSAP

AEISKMPLAAVNTVLALCGATVSTYLTSTMIRKKIGIEDMANAALAGGVAIGSSCAHTTP

KASLILGFVAGILSVIGFALIQPRLQRAIKGIDTCGVHNLHGMPGMLGGLAAIFIAQNVV

PGLQIKGVFITFIIAWITGLAAGTIVSLFGYRKQSYEDAVEFIIEEEHH

>Planctomycetaceae_KSU-1 I3IPX5 |I3IPX5_9PLAN Ammonium transporter protein OS=planctomycete KSU-1 [Planctomycetaceae bacterium KSU-1 : BAFH01000004]

MAIRAIFKLYSVVCIILFCIPLLGIQKASCAAELYEDEFESQALDPSWLIIRENQSDWNL

LVHPGYLRITTKSENLWQINVNNKIKLLRGAPLGDFEVVTKVTYNPKQKFQQAGVIMYES

DDNYVMLTRQMDDVDNVEMSRELSRMSGSKSVPTTLTTLYLKLTKSGENVTGSFSGDGNS

WTTVDVVSGAKLEHPKVGLVGFNAQLNTTVDADFDFFKIGAGGEGMASVLEDFLELTKYG

KAIHVMAMLMVGFGFLMVFVKRYGYGAVTATYIAVSIVIPYYMFLKTQGIFGEPAELKMD

RLILAEFCAASILIATGAFLGRLKMSQYMIMAFMFVPSYMLNEWIMLDNGMGLIPKGLLI

DTGGSIVIHQFGAYFGLGVIVRMTTREDFNKKIESDKISNQFSMLGSMVLWIFWPSFCAA

PAEISKMPLAAVNTILSLCGATVSTYIASTMIRKKIAIEDMANAALAGGVAIGSSCAHTT

PKSSLILGFIAGILSVIGFALIQPRVQRIIKGIDTCGVHNLHGMPGVLGGLAAIFIAKNV

IPGLQIKAVIITFIIAWITGLAAGTVVSLFGYRRQSYEDAVEFIVEEEHH

>Acetivibrio_cellulolyticus 2510779405 Acece_1280 ammonium transporter [Acetivibrio cellulolyticus CD2, DSM 1870 (ORNL annotation) : Contig286]

MKKNVLAFLTGIIFTLLMPFSVLAEEVAANDVVGTVWQFQQYNFSINILAMLLVGFGFLM

VFVKKYGYSATTGTFLVVGTGIPLYLLLRYTGIISSESFDPQSIKALLFAEFAVAAALIS

MGAVLGRLRVYQYALLSVFIIPFYMINEWLVLDGKLGVTQGFVDAAGSIIIHAFGAYFGL

GLAIALTKKEHMNQPIECDATSDRFSMLGSMILWIFWPSFCSAIVPTGDFQKTVVNTILA

LCGATVITYLLSTFLRKGKPSIADIANASLAGGVSIGATCNRVGAPTAFLIGLLAGTICV

IGYVIIQPKLQKALKMVDTCGVHNLHGMPGLLGGIIAIFVVPGAAKPQIIGIVFTVVLAL

AGGFISGNIIKFTGSKLKVYEDSDEFAEAE

>Clostridium_cellulovorans gi|242261927|ref|ZP_04806621.1| Rh family protein/ammonium transporter [Clostridium cellulovorans 743B]

MSELSTKKGVKLTPKSNVNKGLLMFLLVLFIGGALVLIPSINHNVAAGISAINLDGESVQ

ANGAQFSTIFDTLKYERSIHILAMLLVGFGFLMVFIRKHGYSSITATFLVVSIAIPMYVI

IKSFGGESFSKETINIDTLLFAEFAAASLLIAIGAPLGRLKMDQYIIMGLLFVPTYIFNE

WLLLESGMFKGFLDTGGSIVIHAFGAYFGLGVVTNTLSKFKDDDVCEANSNSNQFCLLGS

MILWLFWPSFTSAIVAPDRVVLTAINTIFALCGATLATYIFTKMIRGKIQIEDIANAALA

GGVAIGSTCDVTTPGYAMLIGIAAGALSVIGYSIIAPKLQNLIKGTDTCGINNLHGMPGI

LGGIVAIFITGEASIQLLGIAVTLCVAFVGGKITGIVINLFGKKEIPYSDEDEFFVEQ

>Desulfotomaculum_acetoxidans gi|258514840|ref|YP_003191062.1| Rh family protein/ammonium transporter [Desulfotomaculum acetoxidans DSM 771]

MAKLSTIKETRARDVRYEAKTTVNKGLLILLMLLFIGAALVLIPSINHNVSTGISAINSQ

GGQVNQDSIQYSTIFDTLKYERSIHILAMLLVGFGFLMVFVRHHGYSSLTATLLVVSVAI

PMYTIIKSFGGEGFSKPTISIDTLLCAEFAAAGLLIAIGAPLGRLKMDQYIIMGILFVPA

YILNEWLILDSGMFKGFLDTGGSIIIHAFGAYFGLGVITNTLSKFKGDAVCESNINSNLF

CMLGSMVLWLFWPSFTSAIVAPERVVLTAINTIFALCGATLATYIFTKIIRGKIEIADIA

NAALAGGVAIGSTCDMTTPGYAMLIGIAAGALSVIGYTTIAPQLEKLIRGTDTCGINNLH

GMPGLLGGAAAIFITGKAGTQLSAIVVTVCIAFICGKITGTVIAFLSKKELPYNDKDEYI

VA

>Clostridium_BNL1100 Clo1100_245 stridium_pap 2509527030 Clo1100_0245 Ammonia permease [Clostridium sp. BNL1100 : Clo1100_Contig198.1]

MKKGLFVLLSGFILTLFMPFSAFASANEAVDGIRQVQQYNFSINILAMLLVGFGFLMVFV

KKYGYSATTGTYLVVGAGIPLYLLLRYTGALSLESVNPQTIKGLLLAEFAVAAALISMGA

VLGRLRLYQYALISVFLVPIYMINEWLVLDGGLGITKGFVDAAGSIVIHAFGAYFGLGLA

IALTKQKHMDHPIESDASSDRFSMLGSMVLWIFWPSFCSAIVPTEQFEQTVVNTVLALCG

ATVITYLFSALFRKGKPAIADIANASLAGGVAIGATCNLVSAPIAFLIGLLAGAICVVGY

TVIQPKLQKLLKMVDTCGVHNLHGMPGLLGGIIAIFIVPEAAKAQVAGIVFTVVLAFVGG

IISGNIIRLTGSKEAVYEDADEFEEAA

>Clostridium_papyrosolvens_C7 Cpap_C7_15225 2541788339 ammonium transporter [Clostridium papyrosolvens C7 : ATAY01000076]

MKKGLFVLLSGFLLTLVMPFSAFASTNEAVDGIRQVQQYNFSINILAMLLVGFGFLMVFV

KKYGYSATTGTYLVVGAGIPLYLLLRYTGALSLESVNPQTIKGLLLAEFAVAAALISMGA

VLGRLRLYQYALISVFLVPIYMINEWLVLDGGLGITKGFVDAAGSIVIHAFGAYFGLGLA

IALTKQKHMDHPIESDASSDRFSMLGSMVLWIFWPSFCSAIVPAEQFEQTVVNTVLALCG

ATLITYLFSALIRKGKPAIADIANASLAGGVAIGATCNLVSAPVAFLIGLLAGAICVVGY

TVIQPKLQKLLKMVDTCGVHNLHGMPGLLGGIIAIFVVPEAAKAQVAGIVFTVALAFVGG

IISGNIIRLTGTKEAVYEDADEFEEAA

>Koribacter_versatilis gi|94970623|ref|YP_592671.1| Rh-like protein/ammonium transporter [Candidatus Koribacter versatilis Ellin345](= [Acidobacteria bacterium Ellin345: NC_008009)

MQKKICVLGSACAWWLLGLPAWAQGTSHVETSLRQVEQYNYSIHILAMLLVGFGFLMVFV

KKYGYSATTGTYLVVGAGIPLYLLLRLTGAISAEAMAPNSIHVLLLAEFACASALIAMGA

VLGRMRLYQYAALVAVLVPAYMINEWLVLDGGLGITKGFVDSAGSIVIHAFGAYFGLGAS

IALMRTEHVSHAGQPIESDATSDRLSMIGSMVLWIFWPSFCSAVVPSEQMPQTVINTILA

LCGATLATYVLSALLHNGRTSFSDMANAALAGGVAIGATCNVVSAHGAFAIGVVAGAICV

LGYVYVQPWLLEKIKLTDTCGVHNLHGMPGLMGGLIAIVVIPGIAKAQLAGIATTVVLAL

SVGLFSGYLLRTIGEKKISYEDREEFAGAD

>Clostridium_viride Cl_vir3323 2558434538 ammonium transporter (TC 1.A.11) [Clostridium viride DSM 6836 : Q325DRAFT_scaffold00001.1]

MEPEKSSKNIGILLVFLALIAAMAIAFFPSINQNVAEGMHALGGHAMNTETLSPEVQTIF

DSVTYGKSIHILAMLMVGFGFLMVFIRKHEYSSLTATFLAVSIAVPFYLLIKSFEGEEFS

VISMNGLLFAEFAAASLLIAMGAVLGRLKMDQYFLLGLLFIPAYILNEWLLLESGLFVGF

LDTGGSVAIHAFGAYFGLGLIATTDKKFKGAPLCESDKTSNEFCLLGSMILWLFWPSFTS

SVVSPELGILTAMNTIFALSGSTIATYVFSKLIRGKIEIEDIANAALAGGVAIGSACSSV

NPGFAMVIGLVAGVVSTLGYTIIAPVVCKFIGGTDTCGVHNLHGMPGIVGGLSAILISGT

PGVQLSAILTTVVLALVLGRVAGLIIGLLGVKKTPYSDEDEFFVH

>Eubacterium_acidaminophilum Eubact_acid 2562762796 ammonium transporter [Eubacterium acidaminophilum al-2, DSM 3953 (EAL2) : plasmid] Firmicutes

MSQAAKSEPQYEAQQLASKQASAPSKALLAFFLFIIIAIAVAFLPSFNENLVQEAVDINA

AGANASDSPSEYGTILDTLQYGRSIHILAMLLVGFGFLMAFIRKHGYSSITATFLAVSVA

IPSYMLIKSFGGEGFAMPTVDIKTFLFAEFAAASLLIAMGAPLGRLRMDQYLLMGLLFIP

AYIFNEWLILESGYFKGFFDTGGSVVIHAFVAYFGIGVIANTIHKFENDPICENDSTSNQ

FCLLGSMILWLFWPSFTSAIVTPDKVVLTAINTVFALCGSTLATYIFSTIIRGKVEIEDI

ANAALAGGVAIGSLCDMTTPGYAMLIGIGAGTLSTVGYSILSVKLEKVIKGTDTCGINNL

HGMPGILGGLTAIFVAGTPGIQIIGIITTVAIAFVAGRVTGFVISLFGKKAVAYSDKDEF

VTEHSA

>Nitrosomonas_AL212 gi|255062069|ref|ZP_05314090.1| Rh family protein/ammonium transporter [Nitrosomonas sp. AL212]

MNKSFNPILSGAIGLLLLSLSGLVSAETPVLSEARVVAQYNYIIHILAMLLIGFGFLMVF

VRRYGFGAVTGTYLVVAVGLPLYILLRANGIFGHQLSPHTLDSLLFAELSVATTLIAMGA

VLGRLRVFQYALLALLVVPLYLLNEWIVLDDAIGYTTGFQDTAGSMVIHAFGAYFGLSMS

IVLTTEYQRSKLIESDHTSDRFAMLGSMVLWLFWPSFATALVPLENMPQTVANTLLALCG

ATIATYFLSSKLHHGKTSMVDMANAALAGGVAIGSVCDVVSPTGAFGIGLLAGILSVLGY

VFLQPLLESKFKIVDTCGVHNLHGMPGLLGGLSAFLVVPGIAIGQFNGIMITLIIAIVGG

LIAGAIIKATGTTREPYEDSVEFTHLAGPETEKLPEQLQTRVEALEIQSSTAKPHAPMES

PETKALIARLELRVMTLESNIAATQNRETGEKPESIA

>Nitrosospira_briensis Nitrososp_bri 2556925473 ammonium transporter (TC 1.A.11) [Nitrosospira briensis C-128 : F822DRAFT_scaffold00007.7]

MKKKLCLTLCGVANLFLLCFSAWASESGAGFELNEARQVAQYNYVIHILAMLLVGFGFLM

VFVRRYGFGATTGTYLVVAVGLPLYMLLRANGIFAHEIAPNTIKALLFAEFAVAAALIAM

GAVLGRLRVFQYALLALFLIPAYLMNEWLVLDNGMGLTKGYQDTAGSVIIHAFGAYFGLG

LSLALTTARQRSQPIESDATSDRFAMLGSMVLWIFWPSFATAIVPFEEMPQTVVNTVLAL

CGATLSTYFLSTYFHKGKTSMVDMANAALAGGVAIGSTCNIVSPTGAFAIGLLAGAVSVI

GYVFIQPRLEAHFKIIDTCGVHNLHGMPGLLGALTAIVVMPGIAAAQFIGIIFSVVFAFV

TGLIAGAAIKATGTTRLAYEDSEEFAHCETPDIENIELEVETRT

>Nitrosospira_multiformis Nitrososp_mult gi|82701650|ref|YP_411216.1| Rh-like protein/ammonium transporter [Nitrosospira multiformis ATCC 25196]

MKKSLCMTLCGTAGFFLLYLSAWASEGGTAPQLNEARQVAQYNYVIHILAMLLVGFGFLM

VFVKRYGFGATTGTYLVVAVGLPLYILLRANGVFAHEIAPNTVQALLFAEFAVASGLIAM

GAVLGRLRVFQYALLALLLIPAYLLNEWLVLDNGMGMTEGFQDTAGSIIIHAFGAYFGLG

LSLALTTARQRSQPIESDATSDRFAMLGSMVLWLFWPSFATAIVPFEQMPQTVVNTVLAL

CGATITTYFLSTYFHKGKTSMVDMANAALAGGVAIGSTCNIVSPTGAFAIGLLAGAVSVI

GFVFIQPALERRFKIVDTCGVHNLHGMPGLLGAFVAVLVVPGIAVAQFIGIAFSVVFALI

AGLAAGALIRATGTTRLAYEDSEEFTHTEGPEATEIEAIVLEGETST

>Nitrosospira_APG3 gi|490282294|ref|WP_004178187.1| Ammonium transporter [Nitrosospira sp. APG3]

MTLCSAVSLLLLCFSAWASESAGPELSEARQVAQYNYVIHILAMLLVGFGFLMVFVRRYGFGATTGTYLV

VAVGLPFYMLLRANGILGHEIAPNTVKALLFAEFAVATALIATGAVLGRLRVFQYALLAFFLVPAYLMNE

YLVLDNGMGLTEGYQDTAGSVIIHAFGAYFGLGLSLALTTPRQRSQPIESDATSDRFAMLGSMVLWLFWP

SFATAIVPFEQMPQTVVNTVLALCGATLSTYFLSTYFHKGKTSMVDMANAALAGGVAIGSTCNIVSPSGA

FAIGLLAGAVSVIGYVFIQPALEKRFKIVDTCGVHNLHGMPGLLGALIAIMVVPGIATAQLIGITFSVVF

AFVTGLVAGAAIKATGTTHLAYEDSEEFTHVAPPEIEVVEGGH

>Nitrosomonas_europaea gi|30248465|ref|NP_840535.1| ammonium transporter [Nitrosomonas europaea ATCC 19718]

MSKHLCFTAFSSIALFLLCFSSWASAVAPAEINEARLVAQYNYSINILAMLLVGFGFLMV

FVRRYGFSATTGTYLVVATGLPLYILLRANGIFGHALTPHSVDAVIYAEFAVATGLIAMG

AVLGRLRVFQYALLALFIVPVYLLNEWLVLDNASGLTEGFQDSAGSIAIHAFGAYFGLGV

SIALTTAAQRAQPIESDATSDRFSMLGSMVLWLFWPSFATAIVPFEQMPQTIVNTLLALC

GATLATYFLSALFHKGKASIVDMANAALAGGVAIGSVCNIVGPVGAFVIGLLGGAISVVG

FVFIQPMLESKAKTIDTCGVHNLHGLPGLLGGFSAILIVPGIAVAQLTGIGITLALALIG

GVIAGALIKLTGTTKQAYEDSHEFIHLAGPEDEHKAERLVLEAKTEIQGLKNRIDAAVLS

AKSEG

>Nitrosomonas_Is79A3_Rh 651003848 YP_004695661 ammonium transporter [Nitrosomonas sp. Is79A3 chromosome: NC_015731]

MNKGFCLTLLKVVGLFLLGFSSWVSASEVAAVVSEAQVVAKYNYIINILTMLLVGFGFLM

VFVRRYGFGAVTGTYLVVAVGLPLYILLRANGIFGHQLSPHTLDSLLFAELSVATALIAM

GAVLGRLRVFQYALLALLVVPLYLLNEWLVLDDAIGYTTGFKDSAGSIVIHAFGAYFGLS

MSIVLTTAYQRSKPIESDHTSDRFAMLGSMVLWIFWPSFATALVPLENMPQTVVNTLLAL

SGATIATYFLSSKLHNGKTSMVDMANAALAGGVAIGSLCDVVSPSGAFGIGLLAGTVSVL

GYVFLQPVLESRFKLVDTCGVHNLHGMPGLIGGLSAFFFVPDIAIAQFNGIMITLIIAIT

GGLIAGAIVKATGTTREPYEDSVEFTHLAGPESENLPEQLQMRVEVLETRTAKPQAPVES

PETKALVARLESRIMTLESRVAAVQAQNIQEKPESQT

>Chlamydomonas_reinhardtii Chlamy_Rh1 AAK14647 574aa Huang may 2002 Rh protein [Chlamydomonas reinhardtii]

MQALPPKIPASVSGHGTQSRRHSLDWSHIGLPSRETQLRAGFVPSAAVVIVIFVGLFFGL

TQYTELGTNAQEEVDRFYKYLVDVNIMVWIGFGFLMTFMRRYGYGAVALNYFASALMFLE

AILMIGATQQVFWNYHRTKIQIDIALLIDCAFCAASGMIAFGAIIGKATPTQLLWLLFWQ

VPLYALNQQLVIHTFKALDMGGTIVIHLFGAYYGLAASLMISRKQPLHGLDNPKNSGAYL

NDIFSMIGTIFLFIYWPSFNGALASVSAGHMEEATDAKKAAQFLSIVNTLLSLLGAGLSV

FATSALVGGRFNMVHIQNSTLAGGVAMGAACTLRLTPGGALAVGLGAGAISTLGFQYLMP

FLDRTIGLGDTCGVHNLHGIPAIVGTLVAGLAALGQHPDYLEHDTGRQQLGYQVLAGVVT

MGIAIAGGLLGGFVVSWFNPRGDDPLTVPELFDDGPWWEHQRVEPMPISTSIHLSNMSAH

GKSHHNQSVSVGQLNPIREGREIAVSGVPATGQRSVGEIAVTMQAAPVMASSAPVMGMHA

AAATPIDTPLFADGHAMENAARPVQPMVAGAGNV

>Chlorella_NC64A greenalg 2507981736 e_gw1.6.76.1.1 Ammonia permease [Chlorella sp. NC64A : scaffold_6] chlorophyta (green algae)

MDAEAGTPLLDQAHGHGTPWNLRGTFGASLAGLTALLLGLLAGFGRFAPDIDDTHVGQYY

SYLTDVYVMIFLGFGFLMTFLKRYSYSAVSLNYVTSCLVILEAVLACGWAQQGWGAVAVD

LPLLIDAAFAAGAAMISFGAVLGKATPAQLVWLLALEVPLYAANAQLVAGRWGALDVGGS

ITIHAFGAVYGVAAAAFLAPRGSGSAHPKSGASYVSDMTAMLGTIFLFIYWPSFNGALAS

APGEKAQPQVYCIMNTVVALLGAVLAAFAASAAATGKLDMVHIQNATLAGGVAIGSSANL

AMPPACALAVGITAGALSTAGYLVLSPFLEGKCGITDTCGVANLHGAPGIWGGLASALFS

WLFAAGANKKLIVHGASQPAVQLAALGCTLAAAAAGGALAGFLVSKADPAKQSLEEGDLY

EDAVFWHEVEGEEHKEE

>Coccomyxa_subellipsoidea gi|545369339|ref|XP_005649419.1| Rh-like protein/ammonium transporter [Coccomyxa subellipsoidea C-169]

MAPTSDETAVPLLSGHAHPTWNLRTTFSAPLAVILAALLGVFIAYVKYPELESTDAHVNQ

YYMYYIHVAIMIFVGFGYLMTFLRRYSYSAVGLNYLTSAVVMLEAIVFVGLVQQVIFGDL

SYIELNLPLLIDSAFAAGAAMISFGAVLGKVSPAQITLLLVLQVPIYAFNAHLATEVFGG

LDVGGSITIHAFGAYYGLASALVLAPVAAGSSHPKNGASYVSDMTAMIGTIFLFIFWPSF

NGALASGVPGTISPGPFQQFNCIINTVISLLGATIITFIASAFVGGKFDMVHIQNATLAG

GVAIGSAANLMVGPGGALGVGILAGLISTLGYAYLSPMLEAKIGLRDTCGVHNLHGMPGI

LGGLVAAVTAVLAPEANAPVMKYGGSTQALYQLAALGSTLVIAIVGGLLMGLLVAKLDVA

VAGQELSHEHLFEDCVYWHEVEPEEAEGHAAVNGTAAEE

>Proteobacteria_0000113-E04 _Rh50 D341DRAFT_01802 2518669765 Ammonia permease [Proteobacteria bacterium JGI 0000113-E04 : D341DRAFT_scaffold00071.71]

MAAANEEQMLSHRKQFVGYAAAFQFALALIFAVSTKYGDSANAATGIVVNGTLQNSVQSE

VDHYYPFYQDVHVMIFIGFGFLMTFLKKYTFSSVGLNFIIAAFAIQWSMIVNGVIHNIYE

GHASQKISLGITNLITSDFAAGAVLVSFGAVLGKTTPLQMLIVVIFELLVYACNELIGAV

ILGAVDMGGSIFVHTFGAYFGLALSRTISRSKLNKDGSVKDHPLNGSNYTSDTFAMVGTI

FLWMYWPSFNGALAEESQQHRVIINTVLALCSCCITAFFMDALLREGNKFDMVSIQNATL

AGGVAVGSSSDLVIEPYGAILIGIAAGVLSVIGYVYVQPKLARIWGIDDTCGVHNLHGMP

GILGAVMGGISAGQASENLYGQS

>Naegleria_gruberi Naegleria1corr_heterol gi|290983772|ref|XP_002674602.1| predicted protein [Naegleria gruberi]

MNSTNSTIAETLATNKNRYISGIVISSVFLIVYFIYLIGCFIYYGVKKIKGLTASMKKKE

GGGESTIQLESSSNFNFDLEKKPQEEKTVKEKVTEWIASREYDIEKFSFVFYLVLFELFY

VSMFWIFFEFPTSSTSSQIGDYKNLIDVAMMTLLGLGLSMSCIKNAQYHTIGMTILLAAL

SFQFSIIAQGFWSKIMEYATLKDAKNETVFNTVIYLDMKWLVHGFYGAFAVLISTSCVIG

KTSPVQLILMAFSEVFIYSVNYFCIILVLGAIDYGGSMFIHVFGACYGLAASWSLYKPSV

FQSKVFENHHSKASDMIAIIGTVLLWVLMPSFNAALISNAQYQYRAQMTTFIAMAASCVI

HFVVMRLLRGRFSMRDLQRGAIVGGISMASVQSVIVSPGGALTIGLVAGALTGLGLCVLQ

PLIKHLRIYDTSNVFSTHLLIGVYGGLVSIVATGNAIDNAAKVKIYGDDTKTMFGSKYTN

LWGFQICALCITVVTSIAGGFLHGFVYNKWLFVMSPEKTFEDSAYWIVPTEPKNKDVNNS

VVNSGDQELMVQCTVCSTIEDKLRYSHQELKKQITEKFSELEKKNLTDLSLAKDQNEKQV

NELKEKVKHLEDQIQNLINSLNNSQLELAK

>Dictyostelium_discoideum Dictyo_Rh1_amoeb lcl|Peptide_Contig1119.000001 Eukaryota/Mycetozoa/Dictyosteliida The Dictyostelium Genome Project, blue is uncertain

MTHNDDDHKWVTTKRKEPIFFTVILFIFQIFMIICFAALTGYDTNKNYTGSENPDEFKGG

EVQERVNNFYGYFRDINIMIFFGFGFLMTFLRRYGYSALGYTFIISALVSQWSVLLNGFF

EAWSHSNKHGEFPSTWEFSMDSLLQGFFCSGSVMISYGAILGRVTPLHMLIMGIIEPIFF

FLNVFIGEMNLEAIDVGGGMYIHLFGSVFGLTVAWFLTDRKSKECTDNAPSYSGDNFAMA

GTLFLWMMWPSFNAAIAPLGEPQFRAIANTFLSLTGSTVATFIVSRLFSHLGNKLDMVHV

QNSSLAGGVVQGCIAHMNINPGGAIAMGFIAGTISVCGYLFITPKVQRKLHIQDTCGILN

LHCIPGFLGSIAAIFAAIKGLNNPNMYSKVEFEQIFRAGDSQASANLIATMVSIGLGIVG

GLLVGVILLQLKKIKGLKSKEYYQDSAFWILPIDYPKDVATVVALNNAATSEDTAGGDDE

EEGVGKEHGAVEMGKHNRIVQPKQDNKYHKQLPSDDEEEDEFKQEPI

>Tetrahymena_thermophila gi|67043602|gb|AAY63894.1| Rh-like protein [Tetrahymena thermophila] Eukaryota; Alveolata; Ciliophora

MGHLAEAVTISEQKKFALAIVLIEIFCVIIHGCVAVYDNDPDLPDNAARYPMWQDINVMI

FVGFGFLMTFLRTYQFSAVGYTFVIGAISFQLYPIWEAIWKCAFDLDAPFKIHFNTLTLM

TSSFCAGAILITFGGMIGKVSGFQMAVCIFFETIGFTLNERISINMGVADIGGSMVIHTY

GAVFGLILAKMLSPRSSFGHKKAESNYISDLTSFIGTIFLYMYWPSFNAGPATPGSQAQN

RAYINTMLSLSASCMMTFLISLLTRTEDKKFNMIDIQNATLAGGVAMGTCANMNILPVWA

ILIGALAGIISTFGFSKLQSFLENKIGLHDTCGIMNLHCMPGILGGVWGAVAAAKASSRG

DNAAQLLEVYPKSSDADWSQSKQGGIQVAFLFITIGIAFGSALFTGFMMKLLGNTPTQLF

EDGETFHIPRIRFEDPEEIITKIPKMPHSQVLLVTEQNGSKNNIDGEKIGMIELQNGASN

TISYQTSSAVKN

>Ichthyophthirius_multifiliis Ichthy1_alveo gi|471231448|ref|XP_004036895.1| rhesus type c glycoprotein, putative [Ichthyophthirius multifiliis] Alveolata; Ciliophora

MGKHEINQTSLKEQQKFMTLLVIIEIFCIIFHSVCGQYKTNDFQSYKYPQWQDVNVMIFV

GFGFLMTFLRRYNFGAVAYTFLIGAISFQLYPIWELFFERIWDSDHEFKINMNVSLLILS

SFCSGSILIAYGAIIGKVTPFQLLIMTLVQTIGYTLNERIGLNMGIADIGGSMVIHTFGA

FFGLVVSKIVTPNKAFTHAKAESNYVSDITAFLGAIFLSKLLRHLLMERWLLRIQAIKTE

LSLILYYHQVVLLLRLFLLQQHLEGTCADMHILPIWAISIGFFSGLVSTFGFNKLQSFLE

EKIKLHDSCGVLNLHALPGFLGGIFGSIAASQAQNMGDSSKLISEVFSKSLDDDWNAQKQ

AKLQIAYLFITLILCIIFGFITGLLMKLLGHSPTLLYDDGEFFHIPNIQYEDLAYIMEKV

KETHTLETQQIQTKTSQNYQLQNLQCNQNPEQQPIDT

>Dictyostelium_fasciculatum Dictyo_fasc gi|470250267|ref|XP_004367365.1| Rh-like protein/ammonium transporter [Dictyostelium fasciculatum]

MANKDLIQGIKAKRTESFTFTIALLAFQIFMVVLYAVWVRYSNDDDRKNDPENPSSLENP

VQEDVDNVYGYFRDINIMIFFGFGFLMTFLRRYGYSALGYTFVISAMVCQWSILLYGFFE

TVHHADKNDEYFSSNFQFTLYSMLNGLFCAGAVMISYGAILGRVTPTQMLALGIIEPMFY

FLNLFIGDMVLKAVDVGGGMYIHTFGCYFGLTVAWFLTNRKTKECPDNTASYNGDLFAMA

GTLFLWMMWPSFNAAIAPEGDPQFRALANTFISLTSSTMATFIVSRLLGHYGYKLDMVHV

QNSSLAGGVIQGVLAHMNISPAIAITMGFIAGTISVLGYVFLTPLLQKRLNIQDTCGIHN

LHGMPGFLGSIAAIIACARGINNKYLYGDDYDAIFRAGDDQARANAAATFISVGLAIVGG

LITGFILKLIGRIGQLKTEEYYQDSAFWHVPSDYPKDIESGTAPVGDEENGAVELDIKKA

DLNELKKKGSSRAYRREIIRLLETLVLSEGLARKYDSSESDQDSDGSDSDDDESNSRKNR

RGHHHSKRSHHKNNNNNNRKDEVIPEKKEQEIKIPDSSN

>Polysphondylium_pallidum gi|281211661|gb|EFA85823.1| Rh-like protein/ammonium transporter [Polysphondylium pallidum PN500]

MSSDTPKKLSSKKVENIIFCVCLFLFQVVMIILYSVWVRYDKDSNHDPLPNGESPVQEEV

DNLYGYFRDVNIMIFFGFGFLMVFLRRYGYSSLGYTFLISAMVAQWSVLVWGFYDSMLEH

KELREYYYFTMESLLNGLFCAGSVMISYGALLGKVTPLQMLFMGIVEPVLFFLNMYIMER

LHAFDVGGGMTIHLFGAYYGLSVCWFLTNSKAKHSKENAASYSGDLFAMAGTLFLWMMWP

SFNAAIAPRGHPQMRALANTFLSLTGSTIATFIVSRLFSHYGYKLDMVHVQNSTLAGGVV

QGVMAHLNVNPGPAISMGFIIGSVSVLGYIFLTPFLARRFNIQDTCGIHNLHGMPGLVGS

IAAVIAAAHAINNPDLYSAAEFKVLFPAGDDQAKRNAAMTFIALAVGIIGGIISGFCMKQ

IGKIGKLTKEEYFSDHAFWVVASDYPKEFEGVDPHQYIHDEENGVEMDQAEKRDESMPTT

SDHQQ

>Guillardia_theta Guill1_crypto gi|551656781|ref|XP_005831163.1| hypothetical protein GUITHDRAFT_109968 [Guillardia theta CCMP2712]

MTRHDGYGTQQKIDPARQAQLDQAQALRPPTPAPQESEYGQPDMPFMFDLNMYTFLGMCL

TMTFLRKYAYTSLTMSFLMSCLAQEWCSLLLQWIDASYCESLKTKFALVGCPYSKNPSLT

QFENYQRTRACTCSGLSSINGLPPLALQISDFLKGQYGVVAVLVSYGALLGKVNPQQMML

IVVINVGAYCGNKFLSVDYRGGVDNTGSLYTTHIFGAAYGIGCAIVVSGKGLSKNPDNSS

RYQSNNYAILGSLFMFVTFPSFNSYWAPASLRVFVATNTFAAMMAGGMFNFIWANLVYPE

GPTVMHLQDGVLAAGVAASTPACMFIDPVFMMLVGAGGTLLATLSFHYLQPIIADQDTQG

IASLHLIPGLWGATVMIFVCIFGIDNNTVVRLMEQQELSLSLLNLLQTLRNADLIDTIMP

HYGDKWASTETQTIIVAFSIFVGGLSGAATGVVAKLTCSKSIFHYKSTDSCWFTAKISIG

GSYSDHVFWIVPDDFTHIGEDDAGEKVL

>Oxytricha_trifallax Oxytri1_alveo gi|403366186|gb|EJY82889.1| Rh type B glycoprotein [Oxytricha trifallax] Alveolata; Ciliophora

MGFGRVEITFLLCEAVCILFYGLFTEYKAGTDPKTPGNDEDANRMYMHDKYPLFQDVHVM

IFVGFGFLMVFLKNNSWTSIGFNYLIACWAIQITILFRGFWMQICEHYHHLDHDWHKIQL

NVQELILADFGAGAVLISFGAILGKCSLFQLWVMATIEICFYALNESICIDIFRVSDIGG

SMTIHTFGAYFGISVALFYQPKKAIADKHGVGVGNYLSDLVSMIGTLFLFAYWPSFNGAL

GSGSQQQRTVINTYLSISCSCIAAIISSKIIHHGKLEMEIVLNSSIAGGVAIGACADMIV

QPFGAMLVGFVAGTISSFGYGYLSKFLQEKITLHDTCGVHNLHGMPGIIGGLTSAIVASR

GGDNFGANYNNQFLEVGRSPSEQAGFQLASLALTLGIAITSGLFTGFVTSRQWFQPPPED

YLFDDRYQWADCEIEHEQMADLQKQMSMSGTHSKLANLGKSEADEDDDEDTQRKDNIQ

>Acanthamoeba_castellanii Acanthamoeba1 gi|470482632|ref|XP_004343628.1| ammonium transporter subfamily protein [Acanthamoeba castellanii str. Neff]

MGEDGHEPASKRVISDNVIFASVVVGLEAFFLIIYAIWFEFVTDPVDAAKEVVFYPFFRD

ICIMIFFGFGFLMAFLRRAGFTAIGYSFFLAAVVCQYSIVLDHFFFELGDNPTFSHRRHV

GVENMLNGLFCSGACLISFGAFIGKISPLQLLVLIIVEPFFYWLNFFIGYIKLEAVDIGG

GMFIHTFGCYFGLAACWWLTSKKTHGHPDNCSCYSSDVFSYAGTLFLWMMWPSFNGILGA

DAQEQNRAFFNTFISLCASTAATFVVSRLVSGHRFDCVHIQNSTLAGGVVMGVAAGLDMH

PSAPIGIGFVTGVISVLGYKYLTPFLSRFGIQDICGIHNLHGMPGVLGSMVAMWATLGLS

YDSNEYEELFPRGRGQAGIQAAATAISILLGLGSGFFCGFLMWLVGKLNPIKRADLFNDR

DNWHLPSDYEYVVVKDEDDNNVEMEDMNGVSGSHIRALKNKQVGKVVPAEDGKAPKYSRT

GTVMVDGRDSDSDSD

>Protocruzia_adherens Alveolata; Ciliophora; CAMPEP_0115009076 /NCGR_PEP_ID=MMETSP0216-20121206|22362_1 /TAXON_ID=223996 /ORGANISM="Protocruzia adherens, Strain Boccale" /LENGTH=438 /DNA_ID=CAMNT_0002376745 /DNA_START=64 /DNA_END=1380 /DNA_ORIENTATION=-

MGEKTAASXKSLPKFVKVLGALEXIIILIFITCTELSAEGKVGGGGASNMNYAXFQDVHV

MIFIGFGFLMTFLKSHSWTAVGFNFLVAAFSIQLGFIFQSWWHNILDDNHKKFVLNTLTL

TEADFSAAAVLISFGAVLGKLXALQLVVMAVFEXFFFSININLGLVEFXVADVGGSVFVX

AFGSFFGLAVTKVLSPPSTKDHPKCGDSYNSXLFAMIGTIFLWMFWPSFNGALTXGXTQQ

RVVVNTVLALTGSXIATFLVSAQMHGGKFVMEDILNASLAGGVAIGTSADILLYPGWALL

IGAVAGIISCWGFEKLGGILEAKYGIYDTCGVNNLHGIPGVIGGLVGXLITAFSSEDDFN

XLPGEFWSGMKADDDDRSFGTQAXFQFLALVVTLAIAIXSGYLTGKVITSKHFDAPDDLF

DDKVHWEXEDAEPIPNKA

>Favella_taraikaensis Alveolata; CiliophoraCAMPEP_0185606932 /NCGR_PEP_ID=MMETSP0436-20130131|5138_1 /TAXON_ID=626734 ORGANISM="Favella taraikaensis, Strain Fe Narragansett Bay" /NCGR_SAMPLE_ID=MMETSP0436 /ASSEMBLY_ACC=CAM_ASM_000390 /LENGTH=473 /DNA_ID=CAMNT_0028238679 /DNA_START=1 /DNA_END=1421 /

XTTPFSKTCAMIDSHRATELVYIIAEIIVIVLYLTCTEYGDGVHPGAISTASADDKAEAK

VAKYYPVFQDVHVMIFIGFGFLMVFLKTHSWTSVGFNFVIAAWSMQLSILVVGFWHQALE

KPTDEWQKISLDIPSLIVGDFGAGCVLITMGAVLGKTSLFQMFLLATMEIIFYGLNEAIC

AGHLGAVDMGGSMYVHTFGAYFGLAATYFFDNHKAIKDEFGRAEGGYNSQMIAMVGALFL

WMFWPSFNGALAADFQQQRVIVNTVMAISASCISACAVSRIFLQRLDMEVVLNATLAGGV

SVGSSSDLVVTAGTAMAIGALAGIISAIGFLKLSKFLQEKISLHDTCGVHNLHGMPGVLG

GVLGAVSASLADQPSKVXAALESTFPKLADGRTTSEQGWVQLAALGISLGISIGGGLISG

FIASRFAKVEYLFDDKEHFAHVDYTEVIGEPVTAKEMEAIPPESARGTENNVH

>Pteridomonas_danica Stramenopiles; DictyochophyceaeCAMPEP_0114329780 /NCGR_PEP_ID=MMETSP0101-20121206|1293_1 /TAXON_ID=38822 ORGANISM="Pteridomonas danica, Strain PT" /NCGR_SAMPLE_ID=MMETSP0101 /ASSEMBLY_ACC=CAM_ASM_000211 /LENGTH=370 /DNA_ID=CAMNT_0001459533 /DNA_START=1 /DNA_END=1113 /DNA_ORIENT

TFLRSYGFSALTLNLLVGVFAIQWGILTTGYFETLFSSGADKIPLHLTQLVQGDFAAGAA

LISLGAILGKSTPIQTLXMVLFEIVFYALNFQVGTTMLGAADMGGTIFIHCFGAYFGLAV

SAVISPKDLVPDHKENASVYHSDMFSMVGTLFLWIFWPSFVSVLADGNSLNRCVLXTTLS

IAASGLSACVMSAXXXPDHKMNMVDVQNATLAGGVSIGAVSDHFLGGGGALLIGSLAGVL

STVGYVVLMPILEERMNLFDTCGVHNLHGMPGVLGGLASVFTASYASSALYGDNIGDVFA

EMANGRSAGEQAIAQMSALAVTLFLAITSGIFTGNFINQECFHPPRKIFMDATSFEVPDD

GSYPADSADN

>Crypthecodinium_cohnii Alveolata; Dinophyceae CAMPEP_0194799816 /NCGR_PEP_ID=MMETSP0324_2-20120912|9662_1 /TAXON_ID=2866 ORGANISM="Crypthecodinium cohnii, Strain Seligo" /NCGR_SAMPLE_ID=MMETSP0324_2 /ASSEMBLY_ACC=CAM_ASM_000836 /LENGTH=454 /DNA_ID= /DNA_START= /DNA_END= /DNA_ORIENTATION=

MVSSNAAAIPTGVLPDSESALDHVKDAAKMNRKVDEALTAAGLLALQIFVVVVAGVADFH

YTPHIGAMEXGFXXXVSLMIFFGFGFLMTFLHSYGLSAIGYCLVISAVVVECSMAVEYLV

MDRTSEVSIETLLNGLFCAGAVMISYGAVLGKVTPFQLLSMAALEVVAFWLNFKWSVTDN

VAHDVGGGMVIHTFGAYFGLSVSWWVSQRGAFGHKAEASIYSSDLFSLAGTIILWVLWPS

FQSAVAGTEEKQMLAAANTFLSLCSSTLAFAIVSRFLNGGSRFNVVHLQNATLAGGVVMG

VAGDLDMGLPLTMSMGFVAGALSCVGYAVIQPLLEKLLIHDTCGVNNLHGMPGVLGSVLS

IILVASKPEAWAGEGVTATTQLSALLITLGVALAAGNLTGLLISMPXRRCGVFVPAVEYF

SDASFFEMAPMTPAEAGKEALTPSXDATDARALA

>Scrippsiella_trochoidea Alveolata; DinophyceaeCAMPEP_0115234898 /NCGR_PEP_ID=MMETSP0270-20121206|35028_1 /TAXON_ID=71861 /ORGANISM="Scrippsiella trochoidea, Strain CCMP3099" /LENGTH=564 /DNA_ID=CAMNT_0002649655 /DNA_START=61 /DNA_END=1751 /DNA_ORIENTATION=+

MGASGEAHSVKMDEVLTSVALLVIQIFVVVAACTADLRVSTSLDAKEYGFFRDVSLMIFF

GFGFLMTFLYRHGLSAIGYCLVISAVVVELSVVVEHLVSHGYTEVTIETLMNGLFCAGAV

MISYGAVIGKVTPFQLVVMSVVEVFAFWANFRLIITEVGAHDVGGGMVIHIFGAYFGLAA

TWWVTKKEAVGHGAEKSSYSSDIFSLAGTIILWVLWPSFQAAVAGAEQRQALAMTNTFVS

LCSSTIAFAVISRLLSGHKFNVVHMQNATLAGGVAMGVAGDMDMGLHGAMISGFLAGTLS

CVGYAKVQPMLTELGLHDTCGVNNLHGMPGILGAVIGMVVTIAHGNGGAASPGPLDTMGF

VGVSMQRQVYALLITLGVAMACGTVAGISMTAPLQKLGIFVPSTHYFSDSLFFEEAAPEE

GMCATPSSKSPTGNASTATSVATAGAGALVEPPDRCREVFGSRLRSRSLGADPDGATLAA

SHCHFTVFXSEVHVDHLVSSGGHGVNLVSNSWHDKCVPRPAIGGCRPWHANSSPMPWEGG

GGARGFLLGEVLGAVVARLEYLQX

>Pelagodinium_beii Alveolata; DinophyceaeCAMPEP_0197627444 /NCGR_PEP_ID=MMETSP1338-20131121|6057_1 /TAXON_ID=43686 ORGANISM="Pelagodinium beii, Strain RCC1491" /NCGR_SAMPLE_ID=MMETSP1338 /ASSEMBLY_ACC=CAM_ASM_000754 /LENGTH=618 /DNA_ID=CAMNT_0043198173 /DNA_START=1 /DNA_END=1855 /DNA_ORIENTATION=+

XIFFASQALRIESQRSPQTMAIEKMDAQALRRENIAFCSFLAFVQAAVLCLFLVLGFKHS

AELEANRYAYFRDVSIMIFFGFGFLMTFLRRYGYSAIAYTCIISSTVAELSMVLERLFAD

SSESWEIHIEQLMNGLFCAGAVMISFGALLGKVTPFQLLVMAVIESILFWVNIQVSVVIL

GAHDVGGGMTIHSFGAYFGLAVAASFSGKSSAHHPDNASSYTSDITSLAGTLLLWILWPS

FNAAVAGTEAEQSMAVINTFVSLCAATVATAIFSRLLNDGKFDVVHVQNATLAGGVAMGV

AGDMEAVGLYGAILGGFAAGALSCFGYAKISPLLEKLGIQDTCGVHNLHGMPGVLSAVVG

IVAAANTAGMDASPQIFALLATLGLGLVGGGIAGFVMRMLGSVVGSQLRHDHFNDMSFWT

TPSDYNSVVDEEQGEKTEHIFLYGTMKQGFHNAQHLLDLMARYNGTHAGEGTADLLLFVD

MYGCPYAAFNSAYGRGEKSSPKPVKGELVRFESGMPPELLAKLDEFEQVGQNRYQRRCMM

VRDSSGKSVEAIVYGTEAGRVPGLLQGAFELLSEYSLDFHTSKYVAREKRDITFKQSWGG

YVNARSEISPSPATQVEI

>Geminigera_cryophila1 Eukaryota; Cryptophyta CAMPEP_0179432754 /NCGR_PEP_ID=MMETSP0799-20121207|17297_1 /TAXON_ID=46947 /ORGANISM="Geminigera cryophila, Strain CCMP2564" /LENGTH=612 /DNA_ID=CAMNT_0021210307 /DNA_START=73 /DNA_END=1911 /DNA_ORIENTATION=+

MAMVKRNVNGNEDTIDGDYIPPAPLFADLKAAKVYHDRIHRPPGLKFTDTMILLAAQAFL

LILYGLFVIQDEVPKRPYPDPGVGDKPGMIDFVQPFKRYPPGFDNFFGSARNDEFKLQEH

YSSFMQISYFVFLSLPWSFSFLRKFSYSSATFALFTACVSIQFGIIMMQLVDRMHCIFLE

GLLVSPDFEISDGMLERLNMRDFQYRCQVQRPLDEVEDGFGLRQLRQACYCREWTMLANN

ATRREETPLVAAHALLVTGRRNFQTFSLSLSFMDIVDGLYSTVPTLISFGVLVGKMAPVQ

NVVLAFMNVMAYAFNYWVCVYVMGTFDGTGGAVTTHIFGAFFGMACTAVASPKGAAHDPD

CKGRYQSDIFSLFGSLMIWAYYPSYNSFYAPPAAQQAVAINTYLALLGSSIAGLTASAIF

SGHLKLNIFDAQRSSVAGGVAMGSVANLIAQPWQATLIGALGGVMCSFSGHFIRVFCVQR

LDVHDTVGVMSMHGWPGLVGWLAGIFFLLPLNNDFLSGDLQSKDLAYSLPWENVLQNRKG

TGDAAFVQLVSAPMTISIAMVTGLVAGLVAKKITVLDRKLLFKDSTFFDVPDDFHTNEDD

SEQSDDEKEAAV

>Cryptomonas_paramecium Eukaryota; CryptophytaCAMPEP_0113685994 /NCGR_PEP_ID=MMETSP0038_2-20120614|15019_1 /TAXON_ID=2898 /ORGANISM="Cryptomonas paramecium" /LENGTH=629 /DNA_ID=CAMNT_0000606219 /DNA_START=1 /DNA_END=1890 /DNA_ORIENTATION=- /assembly_acc=CAM_ASM_000170

WESRXLSLLKRPNSRAMGKLSSIKAKLANAXVRLMGGGGNKKKDLSEKVVVGLKPFHIAL

LISAQMAIIIAVSQGVVHETFPKLPAPPPYPAAGEVGGPIFEEAVPVIPFKRYPPGFDVQ

GPRADGTETDRQQERVLQREYKYQMEIVNYVYLGFGMQYAFLRKFGYSTIAFGLLGASIA

SQWGFYWMQLIDNLHCKWLITQYCGEDTTTCGLDPNCYTDFTADQITGTFKQIQXRQACV

CNTYRNFQRNXTEITERPNHARNALLVTGRLDFTTNLVMNSPAIMEALYATVPVQISIGV

LLGKVSPAQMSIMSILCVTAYGVNNWVCIYMLGCYDNIGGCCIIHLFGACFGTGATVFAS

QKGSANNPDNQPRYNADVMAMIGTILNWMTFPSFNAYFAPAVSQQGVVVNTYLSLFSSSV

WVMFFSSLYSGQFKLDPADVQRSSLAGGVAIASCASIFARPHEALIIGAFGGFVCSTAHR

FVRPFFERKLMVTDTVGAISLHALPGLVAWLAGIIYVYPLGQDYRGKWSGQSFITTQTLT

LPYGGEYSTTFMHSEGDGDTAIYQALMAPVTICIGTGTGIATGYVLRKISGPTVAKTFTD

SMYFAVPADFVATEEQDEEPGGSGEKLQV

>Geminigera_cryophila2 Eukaryota; CryptophytaCAMPEP_0179478718 /NCGR_PEP_ID=MMETSP0799-20121207|57127_1 /TAXON_ID=46947 /ORGANISM="Geminigera cryophila, Strain CCMP2564" /LENGTH=698 /DNA_ID=CAMNT_0021289967 /DNA_START=1 /DNA_END=2096 /DNA_ORIENTATION=-

EQCAGGGRPETGRFQSNSFGRACFILSACHTTLSAPSCRPDDLQRVHTGSADTACWPWPE

GSSLGPVSLVALVCVCGAALQLKKRQGTRPAAPSAATMIGSLIKYFRLKYKTYRANKDLK

AKSNNKVISGQTPAHIATNILLQVVLILVFVFGVEHATFPLPADVKDYPGLGEPGGPISI

EAEPVIPFKRYPPGFDVFGPQADGTKSARLKEKVLQYPFKYFIEISTYVYLGFAVQYSFL

RKFGYSTLSFGLLQSTVAAQWGIIWMQQIDNFHCSYLQSSFVNIDVDCSQRYLATEITGS

WEEKQLRQACTCERWASIASNRSMATETPHAAHHALLTVGKMDFTPAIRMTYQSMIEGLL

ATVPVQITYGMLLGKVGPSQLMLCAIMCVTSYGLNYWINMYILGAWDHVGGCCVIHSFGA

FFGIGCTLFASGKGAAQNPDNAPRYNADVLCMVGVILNWMTFPSFNAYFAPAAAQQAVVV

NTYLSQFSSCVAAMVFSSLYSGQFKLDPADVQRSSIAGGVAISSVVSIFAQPWEAMVIGF

IGGGACSTSHHFLRRFLEKKLNITDTVGAVSLHAVPSLVAWISGILFVRDLGTEEMGKWQ

GLQFGPQQTRTMPYDLEYGIIFAHNMGGGETALYQAIMLPTTIAVATVTGMMTGAAARQI

KGPSVARTFSDSIFWVVPEDFRKTEDTTSFGVNTEAAL

>Amphora_coffeaeformis Eukaryota; Stramenopiles; Bacillariophyta CAMPEP_0170708952 /NCGR_PEP_ID=MMETSP0318_2-20130129|5358_1 /TAXON_ID=265554 /ORGANISM="Amphora coffeaeformis, Strain CCMP127" /LENGTH=427 /DNA_ID=CAMNT_0011045217 /DNA_START=111 /DNA_END=1391 /DNA_ORIENTATION=-

MSPTETTSLLPSSGTYGKDEATPSVAASSLVSTLFILLQVALLAFFYIGTEYTTEEYEVK

EYIAFRDIMAMLLLGFGYLMTFLKHYGMGAVGLTMMLSILSIQLNIAVELGVRALYGDDS

KDTAWPLPISMATLIDGEFAAATLMISFGALIGRASPLQMLIIAVSQSLFYAVNKVMFVL

GAVGAEDVGGSMTIHMFGAYFGLACSAALGPAVDLGADEASLPDKVSDVLALIGTTILWV

FWPSFVGATETGVLLNEHHCIVNTILSLLASTTMTFYLTQSLNHGKFDPVHVANSTLAGG

VAIGSAGRLNIGPGCATLTGMLAGAASVYGYKFSSPYLADKWGIQDTCGVGNLHGYPSVV

GATLSIFFIALDPGAEFLSYEMGPQMFRQLMGILVTLGISIASGYATGIVAKSFKDPETT

SYMDKVW

>Hemiselmis_rufescens Eukaryota; Cryptophyta; Cryptomonadales CAMPEP_0173418498 /NCGR_PEP_ID=MMETSP1357-20121228|625_1 /TAXON_ID=77926 /ORGANISM="Hemiselmis rufescens, Strain PCC563" /LENGTH=514 /DNA_ID=CAMNT_0014380995 /DNA_START=1 /DNA_END=1544 /DNA_ORIENTATION=+

XEGEEGEEGSGGRKGGPPEGAMALRINVFIIVLTITQILIIVAYSQLTFNDGYGEQQKLD

EARQKLKDKEDALREPTPAPLESEYGQPDLPFMFDMNMYTYLGMCLTMTYLRKYAYTSLG

MSFLMGCLAQEWCCLLLQWIPLGYCSFLKSNFAEVGCPYGSNPDLTQFENFQRARACTCD

SLEDKIALNIADFIQGQFGVVAVLVSYGALLGKVNPLQMLIIVICNVAAYCGNKFLSVDY

RGGVDNTGSLYTTHIFGAAFGLGCSVVVSGKRPSENPDNAARYQSNNYAILGSLFMFVCY

PSFNSYWAPASLRVYVATNTFAALMCGGMFAFIWSNLIYPEGPSVMHLQDGVLAAGVAAS

TPACMFIPPIFMMLVGAGGTLISALSFRYLQPRIDDQDTQGITSLHLLPGLWGALVMEAV

TILGIDTETVTLNNREMLGEIMPHFGEQWSSAATQALILAFALFVGAIAGALTGLLANLT

GKVALAGSYSDHVFWIVPDDFTHIGEEGADIKVM

>Hanusia_phi Eukaryota; Cryptophyta; Pyrenomonadales CAMPEP_0169522964 /NCGR_PEP_ID=MMETSP1048-20121227|19868_1 /TAXON_ID=3032 /ORGANISM="Hanusia phi, Strain CCMP325" /LENGTH=560 /DNA_ID=CAMNT_0009641173 /DNA_START=67 /DNA_END=1744 /DNA_ORIENTATION=-

MAVRINAFIVTLATVQILLIVAYSILTYHEGYGDQQKLDTARQAQLTKEAALREPTPAPQ

EYQYGQPDLSFMFDMNMYSYLGMCLTMTYLRKYAYTSLGMSFLMGCLAQEWCGLLLQWIP

LAHCSYLKQTFQQVGCPYGDNPDISQFENNLRALACTCDSFSDKIALKISDFIKAQYGVV

AVLISYGALLGKVNPLQMMIIVIMNSAAYCGNKFLCVDYRGGVDGTGSLYTTHIFGAAFG

LGCSVLVSGHRPHENPDNAPRYQSNNYAILGSLFMFVTYPSFNCYWAPAELRMYVASNTF

ISLIAGCFFSFIWANFIYPEGPSVMHLQDGVLAAGVAASTPACMFIPPLLMMIVGAGGTL

IATLSFRFIQPLIEDQDTQGVTSLHLFPGLWGALVFEIVCIVGIDNSWVTLNNSNMLREI

MPHYGEQWTSSATQALVTGFSLLTGLLGGAATGIIAKFAGRIALAGSYSDHVFWIVPDDF

THIGEVDADIKXDVKDRGAPSFIGDGCGPDATAYQLASSGLLTVKHRLVVVTGCQESSGP

MDGGTRSLSTDRSKRSTDKX

>Aulacoseira_subarctica Stramenopiles; Bacillariophyta; CAMPEP_0172428160 /NCGR_PEP_ID=MMETSP1064-20121228|45248_1 /TAXON_ID=202472 /ORGANISM="Aulacoseira subarctica , Strain CCAP 1002/5" /LENGTH=435 /DNA_ID=CAMNT_0013172789 /DNA_START=11 /DNA_END=1318 /DNA_ORIENTATION=+

MASESTPLVSIGGHATPSPAKANAKTLRTLLGTTQLALLILFLTITSYDKFSGYSNLEYV

IYRDIMVMLLLGFGYLMTFLRKYGLGAVGLTMMLTVLAMQLNVFAESFARFLYGKADSLP

LALELPSLIDGEFSAATLLISYGAVIGRASPVQLVVMAICQAFFYAFNKVIIVFGLCEAE

DVGGTLTIHMFGAFFGLAVSHVLGVPKSSSASNASPNRVSDVLALVGTTLLWVYWPSFVA

ATETGVEANNKLCVMHTILGLLGSTGATFFMSQYCKHGLFDPVHVANSTLAGGVAVGASA

RLIMTPGGSLLVGIIAGAVSVLGYSYVTPYLESKFNLYDTCGVGNLHGWPSVFGGLASIV

FVHSNSDAEFLIHGGSGTQGLHQFMGVAGTLVASIVSGWVTGNVMKSYAVEDSEEYDDGI

WWEGEYFEAEEHKQV

>Bigelowiella_natans Eukaryota; Rhizaria; Cercozoa; CAMPEP_0169545056 /NCGR_PEP_ID=MMETSP1052-20121227|9929_1 /TAXON_ID=227086 /ORGANISM="Bigelowiella natans, Strain CCMP623" /LENGTH=484 /DNA_ID=CAMNT_0009666421 /DNA_START=1 /DNA_END=1454 /DNA_ORIENTATION=-

XLGKEPTPENRTYLNHAYLPTPKPMPDAVPLQDVTPATSPHKNADQKLVDSNGRTFDFGK

NTVSHVASANPFLAVLGIAQVGCLILFGLFCDYEDPPDDAATAAAEFVYYNNVAIMMLVG

FGFLMAFLKNYGLGSIGMTFLLTVIILQWTILLEGFWEGVYNNGKFHIIKIGMFDLISGH

FAAATILISFGCLIGKISPVQMCLLGLLELLFYTFNFGITLKWLGVVDIGGSIAVHMFGA

FFGLAAALVWGHTPTKEEESDESSTATSDTFSLIGTVFLWIFWPSFNGAPAAHGTAQQMR

VTINTVLSLAACCTATFFSSRFLSKSHKFGPPDIQNATLAGGVAVGAAANMIIKPWGALA

IGALGGILSTWGFRVAQPFLQNKIGLHDTCGVQNLHGWPSILGAIVSAVVAGFAKESDGY

KDFDSVFPEGNRQAGHQMAGMVLTLLTALASGALCAFIVKMIMPKEIGGGFRDVKYWEVA

DKVV

>Lotharella_globosa Eukaryota; Rhizaria; Cercozoa CAMPEP_0114060902 /NCGR_PEP_ID=MMETSP0041_2-20121206|2320_1 /TAXON_ID=91324 /ORGANISM="Lotharella globosa, Strain LEX01" /LENGTH=459 /DNA_ID=CAMNT_0001145191 /DNA_START=1 /DNA_END=1378 /DNA_ORIENTATION=+

XHNMGSCSDANAGLLNDPVEDAKQDGAAKLDVPHEKFDSFALVAGGAQLLALIFFAAFSD

YAKADETGTDDMLYYNSVAFMVLLGFAYLMTFLRRYALGSVGLTLFLTAMAIQWALLTQG

FWQGVYNGGKFETIKIGIFTLVEATFGAASLLITFGCLIGKMTPTPLAVVAVAQQLAYTF

NVEVTLAWLKPSDVGGTIAIHLFGASFGLGAAWVWGAPSHVGEGHEKSSRASDVFSLLGT

LILWVLWPSFNAAAVSVDDGDGRLRAIINTVMAMSGSCIATFIASRLFSPGRHFGQPEIQ

NSTLAGGVAIGSSANMIVKPAGALAIGIAAGVLSAWGFRRFQPTVAKTLGLHDTCGVMNL

HFMPALLGAVASTLVAAIAKPADGYDHTFNVVFAXGSMQWGYQLAATLMTAVIGGTSGAL

VASVLLTTAGKKEKLCKTDGTFCDQALWEMDEEMRKQHV

>Norrisiella_sphaerica Eukaryota; Rhizaria; Cercozoa CAMPEP_0184485126 /NCGR_PEP_ID=MMETSP0113_2-20130426|6767_1 /TAXON_ID=91329 /ORGANISM="Norrisiella sphaerica, Strain BC52" /LENGTH=464 /DNA_ID=CAMNT_0026866435 /DNA_START=170 /DNA_END=1564 /DNA_ORIENTATION=-

MRENEKGEELLYRHNIQSEGKEFPSISGAEEWFGIDAFGSVACVSQVACIVLFALCVDYD

SSALDASGIQTSNNTLIFFAGVAFMVLLGFAFLMTFLHRYALGSIGMTLLITATAIEWDI

LAEGFWKSAYDGKYQKIEVDVFDLVEGYFAAATLLISFGAIVGKIPPTRILLLSLLEVAI

YALNVEVVLRWLKPADVGGTISIHLFGAFFGLSASYIWGYPPGGAEEEERTSRASDVFSM

LGTLILWVLWPLFNAAAAPSGPGQTRAIINTVLSISSSCIISFAASRLSSPLRKFSPAEI

QNSTLAGGVAIGAVANMIVKPWGAMLIGTLAGLVSVWGFRFLQPRVRSWGLHDTCGILNL

HGIPALVGAAASIVIASVASENDGYKKSESDNFDTIFPHGTPKQGLYQLAATGLTLSVAV

LGGAISSSIVLRACGPSLSDCDGHVMFRDSAFWEMDSDTQLKHL

>Alexandrium_tamarense Eukaryota; Alveolata; Dinophyceae CAMPEP_0116349978 /NCGR_PEP_ID=MMETSP0384-20121206|3564_1 /TAXON_ID=2926 /ORGANISM="Alexandrium tamarense, Strain CCMP1771" /LENGTH=356 /DNA_ID=CAMNT_0003858215 /DNA_START=332 /DNA_END=1398 /DNA_ORIENTATION=+

MIITAMGLQWALFTESFFKQWYHTKEGDAWIDVDINIYSLLQALYAISSVLISFGACIGK

ITPAALILMTIIELACHSFHYVVLGDAVVGLADIGGTYYDHMFGAAFGLSVSYVLGMPRD

DAHDGGQISDVLSLIGTLFLWIYWPSFVGGAAEADSAGQHRALVHTILALSSSTVCAFAS

SMLLSNNNKFRPVDIQNATLAGGVAIGVIANLTLQPIDAILTGGAAGIVSTYGYNVLQPW

LEEKKILHDTCGVNNLHFLPAIVGAIASVIIAGWKSDNRDQDIYGTDAEHHWWMQLVGIL

MTVSFAVVSGLTTGGILKLFGLVDDEVKQFKDSTWWEMNASLHGLGGSTHGTTTAV

>Symbiodinium_CCMP2430 Alveolata; Dinophyceae CAMPEP_0181557540 /NCGR_PEP_ID=MMETSP1115-20121108|8140_1 /TAXON_ID=631057 /ORGANISM="Symbiodinium sp., Strain CCMP2430" /LENGTH=451 /DNA_ID=CAMNT_0023690229 /DNA_START=1 /DNA_END=1354 /DNA_ORIENTATION=-

XWTSGLMATQREVLRYETESPTAAPRPSQTAEELFAPQPGKSFGVLTAVVQLAFIAAFHK

CKLQEEADAALGTDTSLYLGVALMIFVGFGYLKTFLKAYGLGAVGFTLLISCVGIQWALI

LESCLRQADFTIDLPALLRANIDVVPVLVSFGALIGRVSPLQIILLVLIELPCFTVHKVC

LLRQGDARPLVHDGGGTFLHVFGAYFGLAAASSLGPAGKXEAEEQLVPVGYPCLDRHSLS

LDVMAELRRCWPKATRAAQLQALLNTVLALLSSTVMTFGLSQLQDGRLDPQTVQNATLAG

GVAIGATASVVGPFAAAVLGTIAGSLATVGFALSPLFRVVDTCGVHNLHGLPGILGGIYS

ALVPWWYPQSGYVSSHQWVGLLLTLAMAGISGSLCGCILKAVERLEGNEALKRAAEELDL

PASELVLEQFSDDLYWSCAEDVPRSLPLIQL

>Heterocapsa_arctica Alveolata; Dinophyceae CAMPEP_0198058846 /NCGR_PEP_ID=MMETSP1441-20131203|65689_1 /TAXON_ID=192219 /ORGANISM="Heterocapsa arctica, Strain CCMP445" /LENGTH=504 /DNA_ID=CAMNT_0043710995 /DNA_START=1 /DNA_END=1511 /DNA_ORIENTATION=-

LKEGSGSRLARRARRRPRWSLVLGPEEAAMGEAGSKCGGKGRKDDEGEKAAMLHQHHGKR

VAETIEDGDHEPLSRSTGRANTGYFGIAALVTQTILTSLFFFTGYDLTSLTVFNSSQAYD

FYVGIGVMLFVGFGYLNTFLKCYGLSSIGFTMFIGCLGIQWALLLQGWMGTGQFRLAFNV

VSFIRANQSVAAVLVSFGAVLGKTSPLQILVLVMIEMVCVTVNKIYIMDKLGVMDIGGTM

SVHVFGAFFGLFLSFCSAPRNSKNSKGDKVFAPNEDEFGSTYTSDILAMLGTVFMWLFWP

CFNGGDLTMGTAQQKMAIMNTVFALCGSTVTTFAMSTLCTKQQRLTTSPIQMATLAGGVA

VGAVANFAVLPGGAVLIGMGGGAISTCSFLWLHNVLEDKWDTTGVMSLHGIPGLFGAVVS

IFLPLFVKTVPIEAGKQALGLLCVTVNAIICGSVTGYVLAYSGKQPAVAYNDEAYWDVES

XRRAAGGTPPGMAAAAVDYTTTAA

>Florenciella_parvula Stramenopiles; Dictyochophyceae CAMPEP_0119543456 /NCGR_PEP_ID=MMETSP1344-20130328|54118_1 /TAXON_ID=236787 /ORGANISM="Florenciella parvula, Strain CCMP2471" /LENGTH=582 /DNA_ID=CAMNT_0007587741 /DNA_START=137 /DNA_END=1884 /DNA_ORIENTATION=-

MSAAEGGGPVQPHHGPKSFTLFFGMMQTIIIILYCLTTSYDQHTDPKATDNSQRSTYYGM

YQDIHVMIFIGFGFLMTFLRSYGFSSLTLNFLVGIYSIEWGILVVGFFAKAWESDWSYLG

LNIEMLIEGDFAAATALISLGAVLGKTSAMQTLIMVTFELIFYALNFQIGVNGLGAADIG

ATMFIHTFGAYFGLAVSWVMSPKTSKDHKENGSVYHSDMFSMVGTIFLWVFWPSFVSVLA

DGNNQDRAILHTVLAISSSCMCALLASSLLRPDHKFDMVDVQNATLAGGVTIGAVADHYL

GGGGALFVGAAAGILSTIGYVYVQPALEEKIGLFDTCGVHNLHGMPGVLGGLASVVSAAC

AGKSLYGDSLGSVFGAMAPEGNDDGAGEGRSASQQAVNQLLALLITLGIAIASGIVTGMI

MKLATFDSPGDELFNDVTAFGVPGDGPKPSIAPLDAASMTDASRKKGFGLSTAGWMSPKS

RMNQGQNFDPVKGTXDDAKGGRLKSLKCRNTTAWSRAMSTADLPPRTLVEPTLVLSTASA

PGAWRSVHIALAASQSSKQVACSPLVRAFVCMLCGKDSAWVL

>Noctiluca_scintillans Alveolata; Dinophyceae; CAMPEP_0194491752 /NCGR_PEP_ID=MMETSP0253-20130528|10526_1 /TAXON_ID=2966 /ORGANISM="Noctiluca scintillans" /LENGTH=435 /DNA_ID=CAMNT_0039332525 /DNA_START=255 /DNA_END=1562 /DNA_ORIENTATION=+

MAFAVANVCFLAFFGVLVILYGLFTEYATPAEVSRYAFFQDVHVMIFIGFGFLMTFLHKS

GFTAVSHSYLVAVLTVLWAMLVRGFFTIAFSEHAKWETLKLNLEHLVTADFAAGAVLISF

GAVLGRFSATQLLVMSILEVVVYSVNEAILVERLKIADIGGSMVIHAFGAYFGLSVSWAW

AVRDTKQQTKDRSNNRSNKQTDTMAMVGTLFLFCFWPSFNGVLAGTNSQDRAILNTYLSI

CSSCFVSFLVCSLTDDKSRFRMVEIQNSTIAGGVAIGTAADMLTTPCGAMLIGGVSGGLS

VLGYYYVTPVLRSKLGVEDTCGIHNLHGIPGIIGAIVGMIVSAVEQDGEYKNDTLAEVFA

GRFDEEGHLVRSASEQGSFQCAALFVTLGMAIAGGLATGVVMRILPDLDGFYHDAQEYEV

PETPAKVAEQEVGEA

>Platyophrya_macrostoma Alveolata; Ciliophora CAMPEP_0176404794 /NCGR_PEP_ID=MMETSP0127-20121128|2_1 /TAXON_ID=938130 /ORGANISM="Platyophrya macrostoma, Strain WH" /LENGTH=457 /DNA_ID=CAMNT_0017783825 /DNA_START=50 /DNA_END=1423 /DNA_ORIENTATION=+

MPPLSRIEINACIVVWFITILVFGYFSEYSTSVATGDYSMFQXVHVMIFIGFGFLMXXMA

XNSFTAVGHAMIAAAFAVVLSFFNYWLWHNIINHPRDEESWYRFDIGVDQLVNADFCAAA

ILISFGAVIGRVSLKQLLVMVYFEVVFYSINEAIGYGGIGVADVGGSMIIHTFGAYFGLA

VSFIMEKEDTRKKRNEEKGAPDVTPVSDTMAMIGVLFLWCFWPSFNAYFADSLDATKTGM

RQRAIVNTYLSLCASTVATFLTSFVIGKGKLSMVDVQNATLAGGVAMGTSADMLCQPYGA

LIIGTFAGVVSTLGYNFLTPMLDSRIGLKDTCGVHNLHGMPGVTGAIVGIFLTAAATKSD

YDDTGYSWINVWPNAGDTNATSKLVGYQLASLLLTLGIAILGGTLTGILLRKFXTLPTFY

DDHXEYFVHGDDASSGEEPIVPSEIATEMKPIEPSTM

>Blepharisma_japonicum Alveolata; Ciliophora; CAMPEP_0202941930 /NCGR_PEP_ID=MMETSP1395-20130829|2069_1 /ASSEMBLY_ACC=CAM_ASM_000871 /TAXON_ID=5961 /ORGANISM="Blepharisma japonicum, Strain Stock R1072" /LENGTH=473 /DNA_ID=CAMNT_0049637621 /DNA_START=1 /DNA_END=1420 /DNA_ORIENTATION=-

XDRPDGQQSESQKNPMFQIFFILLEVTIVILYGIFVEFHYDPVDPNLPWTEEPQTEFVTG

YPVLQDISVMMFVGFGFLMTFLRTHSWSAVGFNFILTVVVFQMYILYSGFWSRCVSDDHW

DFKIQVGIETLVTAFYCCASILISFGGVIGKLNLFQLLVMAFIEACFYSLNEAIVLEKIH

LEDIGGSITIHTFGAYFGLSVAFIISPKQTKGHPKNTSNYNSNLFAMIGTLFLWMYWPSF

NCFLAKNPEDRIRSVINTLLALTGSATAVFITSSFFKKGKLNMEDVLNATLAGGVCVGSS

ADQIVYPFAALFVGYFAGFISCVGFERLSGFLQRKIGLYDTCGINNLHGMPGVFGGIFSA

IFIGALTEDTLGFRVEDRFDGRTASEQGGLQMAGLGITLGIAIVTGIITGCILKLGCFKG

PIDLFEDQVFWEMEDHQISNGYLPATTREIITDGIELPEKRHKKHKHDKETAD

>Dunaliella_tertiolecta Viridiplantae; Chlorophyta CAMPEP_0202345868 /NCGR_PEP_ID=MMETSP1126-20121109|4914_1 /ASSEMBLY_ACC=CAM_ASM_000457 /TAXON_ID=3047 /ORGANISM="Dunaliella tertiolecta, Strain CCMP1320" /LENGTH=628 /DNA_ID=CAMNT_0048937217 /DNA_START=1 /DNA_END=1885 /DNA_ORIENTATION=+

XWNHSGFPTISWIKSIEVNSCLESLHGQTLDTHVRGSPTGEQRAVMTVVRVMPARACAEA

VTTHVQQQSQVEKPLVEKSNGSLLSEANSEGPLHMVVTEETRGQQLRRNFLPSMGFFVIC

LIPLMFGLTRYVELGVNGQDQVTQYYMWFIHVEIMVFLGFGFLMTFLRRYTLGAIMLNFL

GSCLMFLVAILIVGAAHQTLGTDQDKIKVDLPLLIDCTFCAATGMVAYGAVIGKATPTQL

MWIMVGLVPAFALNQYIVIEHFHILDMGGSNVIHQFGAYYGLAVSFVLSRQRSAHGLQHP

KKSSTYLNDAFSLVGTLFLWIFWPSFNGALASTSSAATTVPSAEGHAASEQFLCVINTVI

SLSGACVATFMTSVLVGGRMKAEHIQNATLAGGVAMGAACSIPITPAGAMVVGMAAGALS

TLGFEYLTPFLDTRLGVRDSCGVHNLHGLPGILGSFVAGLASLGMTTKDYFTRDDCADNT

PRECGGLQLGYQLAGVCVALCISIAWGLIVGIIVTKVNPLKEPELSLDELFDDGPWWHEQ

AVEPMEDAIHPYPHPNQAATLQSNKVSPHANGKHDTSKQLGDVELALDASLPSGASEPPL

IKVSEPASETSPSEEPKPPNLQHKSAWA

>Prasinococcus_capsulatus Viridiplantae; Chlorophyta CAMPEP_0196277150 /NCGR_PEP_ID=MMETSP0941-20130531|4235_1 /TAXON_ID=156131 /ORGANISM="Prasinococcus capsulatus, Strain CCMP1194" /LENGTH=469 /DNA_ID=CAMNT_0041577269 /DNA_START=1 /DNA_END=1410 /DNA_ORIENTATION=-

LVSHPATLKLLASRAMGTKEDVAVTEPLLAAALSEEPQAQAAASSEEIAAPQGPSSFSKS

FAVLLVTVHCVALVGFGLVARADFNEDSATYDAEDAVAVAQRIQVYNYYVGVALMMLIGF

GYLMTFLASYGLGAVGLTMLITALGVEVSLLIEPFFDNWWDTKVTIDTMALFKANLAAAA

FLISFGGNIGKLSPTQLVVMVVFESIFYSVNNQRILGHWLKVKDIGGTISIHMFGAYYGL

AMAWFLGLPPDGRRTKEKASLVSDVIAFIGTLFLWLYWPSFVAGLAEPGTDEAERAITNT

VLGLLGSTVVTFAVSAYCFGVFRPCDIQNATLAGGVAIGATANLTLHPLGALSIGSIAGL

ISAVGFAKVTPCLEATIKLHDTCGIHNLHGMPSIAGALASVVLADVLDDSANDGIIGKWG

AQLAGIGMTLVVSISSGLFTAFILKFLREDVQVADDSPYWEVAEEAKED

>Prymnesium_parvum Eukaryota; Haptophyceae CAMPEP_0195573608 /NCGR_PEP_ID=MMETSP0814-20130614|5443_1 /TAXON_ID=97485 /ORGANISM="Prymnesium parvum, Strain Texoma1" /LENGTH=429 /DNA_ID=CAMNT_0040709507 /DNA_START=1 /DNA_END=1288 /DNA_ORIENTATION=-

XRLVPPVPASPQCSMADTTTRSEPLLSEASQSFWEKHMFSLLLGVVHIALLFFYAFFTTT

NPEEDYLERYGYLVGVTLMMFVGFGYLMTFLRWYGLGAVGLTMIITCLGLELAVLFEGIF

FTTDGSKLINIDLHALLNGDFAVAAFLISFGGLIGKVGPSQLVVLVVCETFAYVANKQLI

LIRWLDIRDAGGTITIHMFGAYFGLAVALVIGKPAKMEMEKASISSDLTSLIGTTFLWLY

WPSFVAGDLKASEGAPLALVNTIISLIGSTVATFIVSPLLSNGKIRPVDIQNAALAGGVA

IGAVANLDIKPFGALIIGSLAGVVSTVGFCKVQERLFRMGLHDTCGIHNLHGMPSLLGGL

ASVFTAVAGIQRAGTAGHQLAGIGLTLLTSICSGLLTGGLLLPFRAPSMNMADDSVHWEV

ADDFEACAP

>Karlodinium_micrum Alveolata; Dinophyceae; CAMPEP_0169337360 /NCGR_PEP_ID=MMETSP1017-20121227|17367_1 /TAXON_ID=342587 /ORGANISM="Karlodinium micrum, Strain CCMP2283" /LENGTH=479 /DNA_ID=CAMNT_0009432875 /DNA_START=1 /DNA_END=1438 /DNA_ORIENTATION=-

QGLARGTAFCYLDLSSSALRYQTKSISGHMADSTPLLTPQEVSGTGKFGVLALVAEVGIL

GLYYQTSYDVSGLKSLDASSIYNFYVGVALMMFVGFGYLMTFLKSYGLGAVGFTMLITCI

GVQWAVVVEQAMVEKTLAFKLDFMDVLNGNFAVAAVLISFGGLIGKVGPTQVLILTLVEL

LFYCANKVYFLTNMLAIADCGGTIIIHVFGAYFGLAACKTLGAATNEKFNGSSYNSDLFS

LIGTVFLWLFWPSFVAGGLPAGTDGHGIALINTVIALLASTVTTFGIMPYLSGKRLTTVP

VQNATLAGGVSIGATANIAMGPFGATLVGIIAGAISCVGFCKPLIASDTDTCGINNLHGM

PGIFGGVVSAVIPFLVKDTGVIAANQAIGLAGTLVVAVITGGLTGFILKCVGGPSRPFHD

SSYWDAADDIKEDRRTYKRERYGATLNPILDAASRLGAFLHPFEEGAKSLIRSFGANPN

>Spumella_elongata Stramenopiles; Chrysophyceae CAMPEP_0184971600 /NCGR_PEP_ID=MMETSP1098-20130426|3811_1 /TAXON_ID=89044 /ORGANISM="Spumella elongata, Strain CCAP 955/1" /LENGTH=501 /DNA_ID=CAMNT_0027493761 /DNA_START=38 /DNA_END=1543 /DNA_ORIENTATION=-

MSVVPTSAILGIDEKNVVSMGSLHAVKFLLTIWQALMIVLLGTCVKVAYYDNGSDFNNHY

QYFTGVEIMMFIGFGYLMTFLKRYGMGALGLTMLITILGLEWGIWIEWFMAKLMYHDNFD

LLELNIGIIHIGLILVTSLLISYGAIIGKVNPLQLVIMTILEALFYSINKAILIETIALV

DPGGSIQTHLFGAYFGLAVSFALGKPSTTTDNETNHVSDIFSFVGTLFLFIYWPSFNGGE

LPSNSHAQQRAVINTILSLLAGSVGTFVMSSLLNSSAKFRPVDIQNATLAGGVAIGTACS

LNLRPSDSMIIGLVAGMLSTFGFARLTPLLEARIGLHDTCGVHNLHGMPGIIAGVSSIVL

IAIKAPLGHDMPEVFIYENQALRQLYALLLTLGISIGSGLLTGFVLKLVGPPKGTDNYSD

FPYWEVHPWERDEVEHHVKDVKDEKNEKNEKNEKNGAHASKDKHGDAVYNKIENGRYSPD

DDEVQEFEYEPKNVSVKNKPK

>Durinskia_baltica Alveolata; Dinophyceae CAMPEP_0170399992 /NCGR_PEP_ID=MMETSP0117_2-20130122|24258_1 /TAXON_ID=400756 /ORGANISM="Durinskia baltica, Strain CSIRO CS-38" /LENGTH=502 /DNA_ID=CAMNT_0010656707 /DNA_START=34 /DNA_END=1542 /DNA_ORIENTATION=+

MNKRIPLSTQGVQSPMHGSSGAVQNSSANVDDVVPGITXEQNFQYATYLLFGMQILMIIL

LGTCAKDNFIGNNNFNNYYQYFTGVEIMMFFGFGYLMTFXKRYGMGAVGLTMLVTVLAME

WGIFTEWFFSMWYESTWTLIPLNILTFTTALDQVAALLISFGGLIGKISPLQLVIMTIVE

CIFYSINKSIFLVGALGTIDAGGTIVIHMFGAYFGLAASYILGKPTKGTENEVNIVSDIT

SLIGTLFLWIYWPSFNGGQQAANSDQQQRAVVHTILALCAATMGTFAASSYFXPSHKFRP

VDIQNATLAGGVAIGVTCNLTMNPIDPLIIGLVAGVVSTYGFNVIQPILESYGVHDTCGV

NNLHGMPSLVGGLASVFITAYKGPRGSDMPDVFNYPGQAGVQIGAMCITXIIAVSSGIFT

GLIMRKYGCGTAAENFSDFPYWEVEAFEGEEHKPEGAHTATDGDVEMGQGSAVGGNKKYD

SVPRSSDEANLEDDVEVQHVKL

>Ochromonas_BG-1 Stramenopiles; ChrysophyceaeCAMPEP_0173139106 /NCGR_PEP_ID=MMETSP1105-20130129|4069_1 /TAXON_ID=2985 /ORGANISM="Ochromonas sp., Strain BG-1" /LENGTH=535 /DNA_ID=CAMNT_0014051791 /DNA_START=46 /DNA_END=1653 /DNA_ORIENTATION=+

MTTDNHVIISQVDPDKEKIEAWTQSSLRNASILLGCFQIMMIILFASCSTFVETNADSTG

TITQGYTYFIGVEIMMFIGFGYLMTFMKCYGLGAVGFTMLITAIGIQWAIFTESFFAQMM

EKNGTREWHNVEIDIYVLMDTLFAISAVLISFGALIGKIKPFQLVIMALLEIAFHSFNYE

CILTGAMEVMDIGGTYADHMFGAYFGLAVAWSLRKQKNHVEPAVGYVPDIFSFIGTLFLW

IYWPSFVGGAAQADSVEQQYAVMNTILALSASTVMTFFMSSIMAKDIKFRPVDIQNATLA

GGVAIGCSANFNMNPVNAIFIGAAAGLWSTFGYNVIQPYLFEKIGLHDTCGVHNLHAMPS

VIGAIASIILVAYKQTGGRRHDADIFADKDGQSWRQLVSILLVITFALVFGYLTGLFLQW

IEPNSPEDFDDSPYWEVAHDFTYVNSETMDSEGTDLEFNKLVDPKKEEKERLIEMIPQGS

NHSKLSRSNHSNRLNASNHSNRLNASNHSNRLNASNHSRLSASAHAKNSAVETQL

>Extubocellulus_spinifer Stramenopiles; Bacillariophyta CAMPEP_0178647928 /NCGR_PEP_ID=MMETSP0698-20121128|20187_1 /TAXON_ID=265572 /ORGANISM="Extubocellulus spinifer, Strain CCMP396" /LENGTH=437 /DNA_ID=CAMNT_0020289219 /DNA_START=94 /DNA_END=1407 /DNA_ORIENTATION=-

MPTETSPLVIDSGASDLSSDFALANSKVLTRLLGTTQAALILLFLFGTTYDSRDYSPSEY

VXFRDIMVMLLLGFGFLMTFLAKYGLGAVGFTMMVSAIAVQLNIFVELFCRFIYGXGGSD

ETMFPLPLKVPTFIDGEFSAATLLISYGAVIGRASPVQLVIMALCQSLFYAFNKVVIVLG

LLAAEDVGGSMTIHMFGAYFGLAVSYVLGPPKATSASNADPNSVSDVLALIGTTLLWVYW

PSFVGATETGVPLNENVCVMHTVLALLGSTGASFYMSQKLCHGKXDPVXIANSTLAGGVA

VGSSARLAMTPGGALLLGVVAGVVSVYGYVYSTPMLEKKLGVFDTCGVGXLHGWPSVLGG

LASILFVALDSDAEFLKXSGFWQCLRQFVAVVCTIAFAVGSGSFTGTVMKKADDDTPDQY

DDGVWWEGEYFEQDEEK

>Dactyliosolen_fragilissimus Stramenopiles; Bacillariophyta CAMPEP_0184862784 /NCGR_PEP_ID=MMETSP0580-20130426|7728_1 /TAXON_ID=1118495 /ORGANISM="Dactyliosolen fragilissimus" /LENGTH=507 /DNA_ID=CAMNT_0027360767 /DNA_START=39 /DNA_END=1562 /DNA_ORIENTATION=+

MSVSSIGSYSVSMKKKRNAKIGVSLAKDETNGDIRVTRVAESGLAAKSNLRAGDKIMSIN

GKSVEGLSPEAVAAMLRASAGHIDIEAEYADDEEMPTEDDNLLSKEVNTDYAVGLSGWLG

FLIFLIQVAFVVYAGVFMTIDTENSTKAEYMIFRDIMVMLLLGFGYLMTFLSKYGLSSVG

ITMLATVLNMECNLIFESLFLNKYALSMSSLINAEFSAATLLISFGALIGRVSPLQVTLI

AVFQSLFYVINKVIVLNYLQVEDVGGTVTIHMFGAFFGLAASYALGPQLKVSASNNAATR

VSDVFALIGTTLLWVYWPSFVGATETGNAVYEPRCTIHTVLALIGSTASAFYFSQKQNKG

KFDPVHIQNSTLAGGVAIGATARLALSPACAVITGLIAGAVSVWGYDYSSPALESSMGIY

DTCGVGNLHGFPSVVGGLLSVIFVVIYSDDSIMAHSSSQFVFQIIGIFTTLIGSIVTGFI

TGKIVLNNSPAYDDVEDYEDHVFWTSE

>Partenskyella_glossopodia Eukaryota; Rhizaria; Cercozoa CAMPEP_0197525094 /NCGR_PEP_ID=MMETSP1318-20131121|10617_1 /TAXON_ID=552666 /ORGANISM="Partenskyella glossopodia, Strain RCC365" /LENGTH=540 /DNA_ID=CAMNT_0043078261 /DNA_START=1 /DNA_END=1625 /DNA_ORIENTATION=-

XRVKERAATSMQEVELQAKDNNNHSPISKNRGSVSSNRYNGNGDDFSPSGRERSSMNNQI

FIVDDRERKFGGKPLAAGSTAELMEVDRHGKIVPKKKKAADSKENGGKQGGDEDGGVGLT

DPFLVIFTTIQCLSLLFFGLFTDYASTPEDGATAASEFVYYNNIAFMMLIGFGFLMTFLK

RYTLGSIGMTFLLTVMMIQLTILLEGFWEGVYNKEFHTIKVSVFSLVSGNFGAATILITF

GCLIGKISLPQMAVLGVLELWAYTFNFGIVLRWLKVVDIGGSIAIHMFGAFFGMAAAWVW

GYKPGPDDEAYESSSRTSDVFSLIGTIFLWVFWPSFNGAPAPNGSAQQLRVTINTVLALC

ACCQATFIASRFLSKNKGKFSPADIQNATLAGGVAIGSSANMIVQPWGAIAIGALGGLIS

TFGFRVTQPWIQGKLGLHDTCGVQNLHGWPSIIGGLTSAVVAAFAEKSDGYEDFNAVFPE

GNRQGGVQVAGVVLTIAIAVASGALAALIVKFILPPKEDAKAFRDSGEWEVDEEEALVVH

>Reticulomyxa_filosa_corr gi|569360466|gb|ETO07715.1| ammonium transporter Rh type A, partial [Reticulomyxa filosa]

VGERRYENMGTVAILISMGALLGRVTPSQLLLMALIEVPLYAINFYIVHHYLRMTDAGGS

ATIHVFGCYFGLAASLIIGKPEHMTPSTSIYHSELLTMIGTLFLWLYFPSFNAYFAYAAP

IQESGYDGLRRQSDRTRAFVNTVIALCASTIAAFIVSKCNRKGKINMTYIQNATLAGGVA

MGACADLYTNPAGAVAIGFFAGVLSTIGFKLYETACPSVGLYDTRGVNNLHGMPGILGCV

ASAITVGSFNDKSRYRRDMHHQGAYQIAGLCVTFGIAVVGGLITGLLMKVLPSPKVAYND

SEHFIVAETDYYYGTIRGKQKGASD

>Phaeodactylum_tricornutum Phaeodact_1_stram (Eukaryota_Stramenopiles_ Bacillariophyta(Chromalveolata/Diatoms) PHATR_12_PE252 tr|B7FSF9|B7FSF9_PHATC (Fragment) OS=Phaeodactylum tricornutum (strain CCAP 1055/1) GN=cc1 PE=4 SV=1 Eukaryota_Stramenopiles

YIAFRDIMAMLLLGFGYLMTFLKNYGIGAVGFTMMLSILAMEANIPMELIMRTLKGDDGE

DTSWPMPLSMETLIDAEFSAATLMISFGALIGTATPLQMMLIALSQSFFYALNKVFFVFG

MVGAEDVGGSMTIHCFGAYFGLAASLAMGRGRSDGKDRIKEELGTPEPDKVSDLMALIGT

TILWVFWPSFVGATETATPEYEMRCVVHTITALLASTTVTFYLSHRLCHGKFDPVHVANS

TLAGGVAIGSAARLDLGGPGGAIIVGMCAGAVSVYGYVYSSPYLESSWHISDTCGVGNLH

GYPSVVGALLSIVFVA

>Acropora aug_v2a.12269.t1 cnidaria

MTFLKKYGYGAVGYNFFIASLVTQWGTIVSGCFNQLYSEGKKHIELSIQTLVTAEFAAAT

VLITYGAVLGKVSRLQLLVIGILEVVFYAINELVAIEFLKFSDAGGSILIHTFGAYFGLA

LSRVLHNKDAHGSHKEGSNYHSDLFAMIGTVFLWMYWPSFNAALVAPDYVAQHRSVINTY

FSLSAACVTTFAISPIFQRRAGKWRMSMVHVQNATLAGGVAIGTASNMSVSPWGALLIGC

CAGGLSTVGYAYLTPFLTKYTKTHDTCGVNNLHGMPGILGAIAGAIAAAHANVDKYGSEG

LKSLFPAMVGDGRTAKVQAGYQLAGLAVALGIAIIGGIITGFVVRWQIFDPPTREQMYDD

EDFWEVPTEDDVEAPNAGSSLMVGDDKV

>Hydra_vulgaris gi|449669287|ref|XP_002167946.2| PREDICTED: ammonium transporter Rh type A-like [Hydra vulgaris]

MKQVGISFPLALIAFQVLLLALFAGFVDYDYTKSGHNNKFYAQFQDIHVMIFIGFGFLMT

FLKKYGYGALSYNLLLAAATIQWATLINAWIKQRMQKNSNHFEGNPTKIKVGVTDMITAD

FTATAVLISYGAVIGKASRVQLFVMTIVECVIFAINENIIMEYLKISDVGGTIVLHVFGA

YFGLAVSFILKNHSDLKHKEGSEYHSDIFSMIGTLFLWVFWPSFNAGLLGDKSIQQSRAL

VNTYFSLAACVLTSFACSSFVNKKFKLNMVHIQNATLAGGVAVGTCADLMIKPWGAIMIG

MFAGCISVLGYNYLTPILNKHKIHDTCGVHNLHGMPGVFGALCGAVAAAAIKPKSYYDKD

E

>Nematostella_vectensis Nematost2b gi|156375207|ref|XP_001629973.1| predicted protein [Nematostella vectensis]

MSRPTFTILTILLQVLFVVIFAIFGEYGDDARPNHKRPNPGAAGINAVNIYYPMFQDVHV

MIFLGIGLLLAFLRKHAYSSISYCFFAAAILCEWSTIINGVFWHIIEGGNDKFKIDLFSA

INADFAAAVILISYCVVLGKISILQLLVMGVIELAVYVLNSWICFAKLGISDIGASITIH

MFAAYFGLGVTRVLHSRDSEGNGKECSSYHNDVFCLVGTIFLWLYWPSFNACLTTDDVMR

HRTVTNTYYSMLGACVMVFALSPMFRRDGKFNLSHVQNATLAGGVAIGTASNMIVQPWGS

MLIGSIGGAMCTLGYVYLSPFLKKHCKMHDVCGVHNLHALPAFLSGIASAIASSLAAADE

YGDATPGQQAGFQVAAMFVTMGISLLSGILTGKEVYIL

>Elphidium_margaritaceum Rhizaria lcl|CAMPEP_0202692502 /NCGR_PEP_ID=MMETSP1385-20130828|6863_1 /ASSEMBLY_ACC=CAM_ASM_000861 /TAXON_ID=933848 /ORGANISM="Elphidium margaritaceum" /LENGTH=484 /DNA_ID=CAMNT_0049348041 /DNA_START=66 /DNA_END=1520 /DNA_ORIENTATION=-

MAAGNARDEAYYTQEVNVNKRENDKHGGTVRQERQFMIVAISFQILLLILYAISTDYSDG

TVAAHSDDHEWGTEPFYFYGMYQDVHAMMFVGFGFLMTFLRRYGFGALTFNFLLCAFGIQ

WGMYIFHMIPWVFNEHIGVLHINTYKLVDGDIATAVVLISFGALLGRVNPAQLLVMCFFE

LFFWALNFHICVEMLHIVDVGGSIVVHTFGAYFGLAVSFVCGLPKHDDENKSMYHSDMFS

MIGTLFLWLYWPSFNGFFANREYYYMDRAFVNTVLSLCGSTVATFIMSRVAKKGKFDMVH

IQNATLAGGVAVGAAADLYLHPAGAIGIGFVAGVLSVCGYEYLSDLLKDRLNVEDVCGVH

NLHGMPGIFGGIVSAIALAIADGTGVYPTAEDTYPFAHPLQKQAAFQISGLCVTLGIAVL

SGLMVGMFVKCMEFPSNEFTDYGHFVVPKDEAQVDFFRTTIRKANFSAIRPVDDFDALKA

DDTE

>Ammonia_29189 Rhizaria_foraminifera lcl|CAMPEP_0197031730 /NCGR_PEP_ID=MMETSP1384-20130603|10640_1 /TAXON_ID=29189 /ORGANISM="Ammonia sp." /LENGTH=502 /DNA_ID=CAMNT_0042461299 /DNA_START=30 /DNA_END=1538 /DNA_ORIENTATION=-

MTTDAVELPLTKNRDHVISNGSKPAGHGGGGGLKRKPREEKVSYGPADIQFLSIAVAFQV

LLLVLYAISSDYADATVSVDPHDEEHGTEPFYFYAMFQDVHAMMFVGFGFLMTFLRRYGF

GALTFNFCICAFGIQWGMYMFHMIGWWFNEQPGIFHVTAYKLVDGDIATAVVLISFGAVL

GRTNPAQLLVMCFFELIFWALNFYICVEYLGIVDVGGSIVVHTFGAYFGLAVSFICGQPR

HDEENKSMYHSDIFSMIGTLFLWLYWPSFNGYFANREYYFMDRAFVNTVLGLCGSTVATF

TVSRLAKKGKFDMVHIQNATLAGGVAVGAAADLYLHPAGAIGIGLIAGSLSVCGYDYLSD

IMDDKLKIADTCGVHNLHGMPGILGGIVSAIALAIADGTGVYVPMSDPSYPFGEKTLQQQ

AGYQISAICVTLGIAILSGMGTALIIRTMKFPKNEFKDEDNFIVPSGNKDIFRGTIRQQH

FGGRELDDFAALDNDFDDDEDE
